# Micro-flow photosynthesis of new dienophiles for inverse-electron-demand Diels–Alder reactions. Potential applications for pretargeted *in vivo* PET imaging†
†Electronic supplementary information (ESI) available. See DOI: 10.1039/c6sc02933g



**DOI:** 10.1039/c6sc02933g

**Published:** 2016-10-07

**Authors:** Emilie M. F. Billaud, Elnaz Shahbazali, Muneer Ahamed, Frederik Cleeren, Timothy Noël, Michel Koole, Alfons Verbruggen, Volker Hessel, Guy Bormans

**Affiliations:** a Laboratory of Radiopharmacy , Department of Pharmaceutical and Pharmacological Sciences , KU Leuven , Campus Gasthuisberg, O&N2, Herestraat 49, Box 821 , 3000 Leuven , Belgium . Email: guy.bormans@kuleuven.be; b Micro Flow Chemistry & Process Technology , Chemical Engineering and Chemistry Department , TU Eindhoven , P. O. Box 513 , 5600 MB Eindhoven , The Netherlands; c Nuclear Medicine and Molecular Imaging , Department of Imaging and Pathology , University Hospital and KU Leuven , Herestraat 49, Box 7003 , 3000 Leuven , Belgium

## Abstract

Pretargeted PET imaging has emerged as an effective two-step *in vivo* approach that combines the superior affinity and selectivity of antibodies with the rapid pharmacokinetics and favorable dosimetry of smaller molecules radiolabeled with short-lived radionuclides. This approach can be based on the bioorthogonal inverse-electron-demand Diels–Alder (IEDDA) reaction between tetrazines and *trans*-cyclooctene (TCO) derivatives. We aimed to develop new [^18^F]TCO–dienophiles with high reactivity for IEDDA reactions, and favorable *in vivo* stability and pharmacokinetics. New dienophiles were synthesized using an innovative micro-flow photochemistry process, and their reaction kinetics with a tetrazine were determined. *In vivo* stability and biodistribution of the most promising ^18^F-radiolabeled-TCO-derivative (**[^18^F]3**) was investigated, and its potential for *in vivo* pretargeted PET imaging was assessed in tumor-bearing mice. We demonstrated that **[^18^F]3** is a suitable dienophile for IEDDA reactions and for pretargeting applications.

## Abstract 2

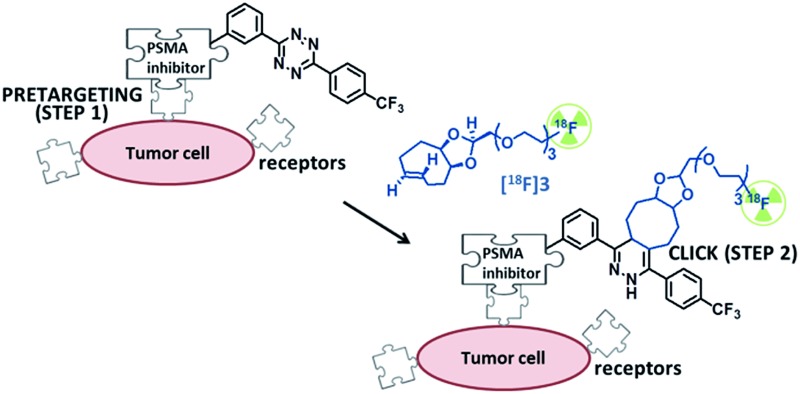
New dienophiles were prepared with an innovative microfluidic setup. **[^18^F]3** is suitable for inverse-electron-demand Diels–Alder reactions and pretargeting applications.

## Introduction

In the field of cancer research, radiolabeled monoclonal antibodies directed against tumor-associated antigens have emerged as promising vectors to visualize or treat cancer lesions, due to their high affinity and specificity.^1,2^ However, because of their high molecular weight (∼150 kDa), antibodies usually have long biological half-lives, thus requiring multiple days to clear from blood and non-target tissues, and to reach an optimal accumulation in tumor. Therefore, radionuclides with relatively long physical half-lives must be employed to radiolabel antibodies for direct *in vivo* imaging. For instance, zirconium-89 (*t*_1/2_ = 78.4 h) can be used for positron emission tomography (PET) imaging.^3^ This however leads to high radiation doses in healthy tissues for patients.

On the other hand, fluorine-18 (*t*_1/2_ = 110 min) is the PET radionuclide of choice given its favorable properties including its decay mode (97% β^+^ emission), low positron energy (634 keV maximum), and short β^+^ trajectory in tissues (<2.3 mm). Its half-life is long enough to allow multistep syntheses but is short enough to avoid extended irradiation of patients. Moreover, it can be produced in large quantities (>400 GBq per batch) with a cyclotron.^4^

Combination of antibodies with fluorine-18 is challenging, due to the incompatibility between the long plasma half-life of the antibody and the short physical half-life of the radionuclide. Pretargeting addresses this issue in two steps.^5^ First, a non-radiolabeled antibody modified with a tag is administered. Few days later, when the antibody has reached a maximum uptake in the tumor and a sufficient clearance from non-target tissues, a relatively small radiolabeled molecule is injected. The latter has the property to selectively bind to the antibody *via* the tag, while the non-bound radiotracer is rapidly cleared. Then, high contrast images can be acquired few hours after the injection of the radiolabeled molecule. The overall radiation dose for patients is thus reduced, compared to the use of an antibody directly labeled with ^89^Zr for instance.

**Fig. 1:**
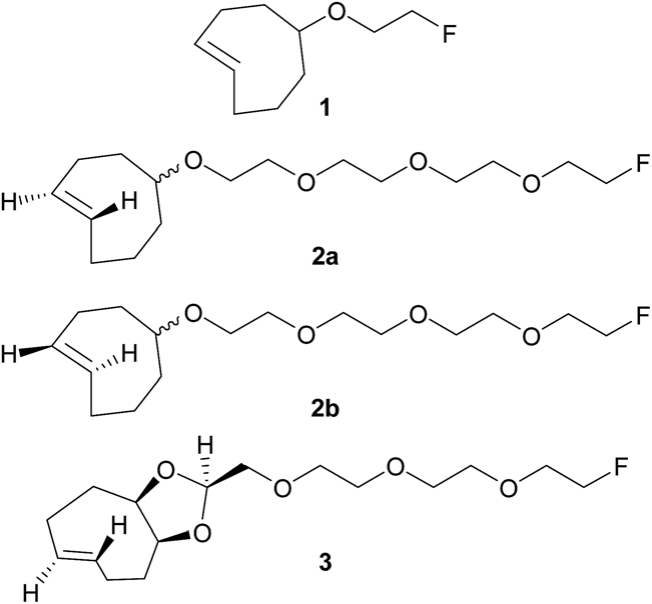
Chemical structures of previously reported TCO derivative **1** ^9^ and newly developed dienophiles **2a**, **2b**, and **3**.

Pretargeting approaches can be based on an inverse-electron-demand Diels–Alder (IEDDA) click reaction between 1,2,4,5-tetrazines and *trans*-cyclooctene (TCO) derivatives.^6^ Indeed, this fast, selective, high-yield, biocompatible, and bioorthogonal reaction has already proven to be suitable for this kind of applications, both *in vitro* and *in vivo*,^7^ even using ^18^F-labeled tetrazines.^8^ However, to the best of our knowledge, no *in vivo* pretargeting PET imaging results have been reported on using a ^18^F-labeled small TCO compound, although this approach may have specific advantages with regard to pharmacokinetics and stability. Some radiolabeling procedures were developed for TCO **[^18^F]1** (Fig. 1)^9^ but it was applied as a prosthetic group for subsequent ^18^F-labeling of biologically-active molecules.^10^

Wyffels *et al.* explored biodistribution of **[^18^F]1** in healthy mice, from 5 to 240 min p.i.^9*b*^ Results demonstrated renal and hepatobiliary clearance of radioactivity, slow blood clearance, as well as increasing bone uptake values (from 60 min p.i.). The bone uptake is due to defluorination, as [^18^F]F^–^ is known to have a high affinity for bone, and is an indication of tracer instability. Therefore, we aimed to develop new TCO derivatives, with improved *in vivo* stability, favorable pharmacokinetics, and high reactivity for IEDDA reactions.

We designed compounds **2a**, **2b** and **3** derivatised with polyethylene glycol (PEG) chains (Fig. 1) with the aim to increase their hydrophilicity and their stability towards enzymatic degradation.^7l,11^ We developed a conformationally-strained dioxolane-fused *trans*-cyclooctene (**3**), encouraged by the results reported by Darko *et al.* ^12^ Indeed, it was demonstrated that this strained *trans*-cyclooctenes react faster with 3,6-diphenyl-*s*-tetrazine than non strained analogs, and display excellent chemical stability in aqueous solutions and plasma. Moreover, dioxolane-fused *trans*-cyclooctenes can be prepared easily through diastereoselective synthesis.

Herein we report (i) the syntheses of new TCO derivatives, *via* a *trans*-for-*cis* photoisomerization step using an innovative micro-flow process; (ii) reaction kinetics of these new dienophiles with a tetrazine, as well as their stability in aqueous solution; (iii) ^18^F-radiolabeling of the most promising TCO derivative **3**; (iv) *in vitro* stability of **[^18^F]3** and *in vivo* biodistribution studies after injection of **[^18^F]3**; (v) proof of principle *in vitro* and *in vivo* pretargeting experiments using **[^18^F]3**.

## Results and discussion

### Syntheses

**Scheme 1:**
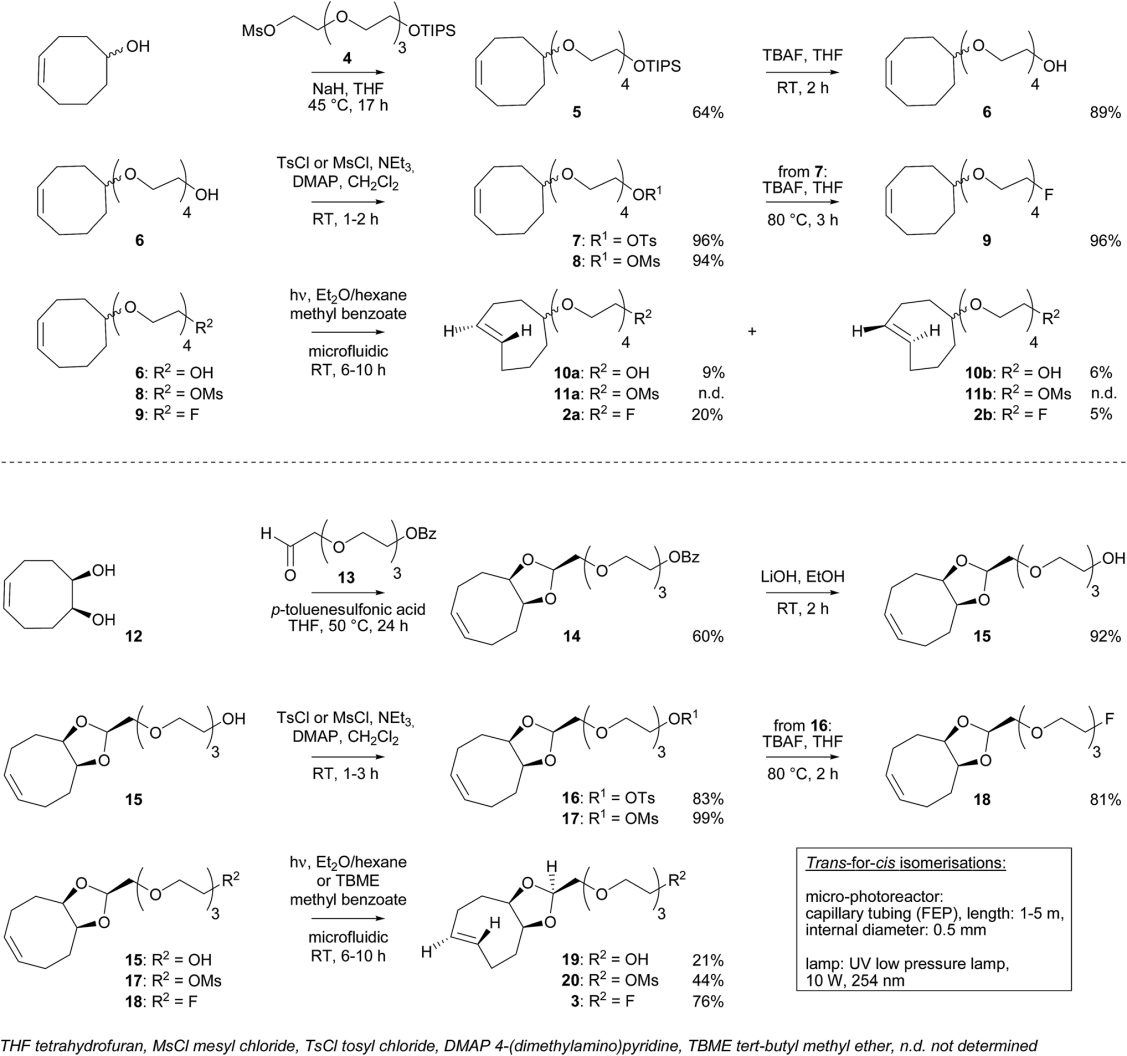
Syntheses of dienophiles **2a**, **2b** and **3**.

New dienophiles **2a**, **2b** and **3** were prepared as shown in Scheme 1. First, the corresponding *cis*-derivatives **9** and **18** were synthesized, in 6 and 7 steps respectively.

PEG synthon **4** was obtained in two steps starting from tetraethylene glycol: protection of one hydroxyl group using triisopropylsilyl (TIPS) chloride followed by mesylation of the other hydroxyl group. Then, synthon **4** was used for nucleophilic substitution with *cis*-cyclooctenol to yield derivative **5**. The choice of TIPS as the hydroxyl protecting group was important to obtain a good yield. After deprotection using tetrabutylammonium fluoride (TBAF), the hydroxyl group of compound **6** was replaced by fluorine (**9**) *via* a sulfonate intermediate.

For the synthesis of **18**, PEG synthon **13** was first prepared in two steps starting from tetraethylene glycol: after protection of one hydroxyl group using benzoyl chloride (BzCl), the other hydroxyl was oxidized to an aldehyde in the presence of Dess–Martin periodinane reagent. In parallel, oxidation of 1,5-cyclooctadiene into diol **12** was carried out using cetyltrimethylammonium permanganate. Then, PEG synthon **13** and diol **12** were involved in an acetalization, leading to dioxolane **14**. The stereochemistry of **14** was determined according to Darko *et al.* ^12^ After deprotection using LiOH, the hydroxyl group of compound **15** was replaced by fluorine (**18**) *via* a sulfonate intermediate.

**Scheme 2:**
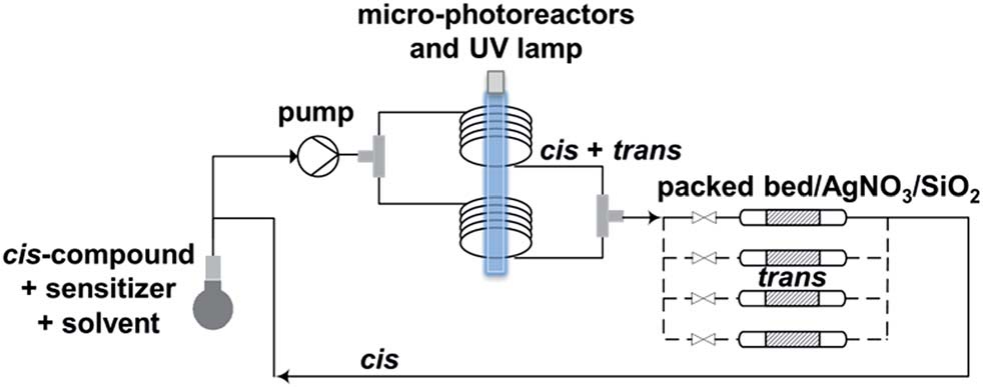
Microfluidic setup optimized for *trans*-for-*cis* isomerization of functionalized cyclooctene derivatives.

*Trans*-for-*cis* isomerization of hydroxy-derivatives **6**, **15**, sulfonate precursors for radiofluorination **7**, **8**, **16**, **17**, and fluoro-derivatives **9**, **18** was performed using an innovative micro-flow photochemistry process. Basic design of the setup was based on the work of Royzen *et al.* ^13^ This group devised an apparatus where the reaction mixture, containing a *cis*-cyclooctene derivative and methyl benzoate (a singlet sensitizer), is photoirradiated at 254 nm, and continuously circulated through a bed of AgNO_3_-impregnated silica gel. The *trans*-cyclooctene derivative forms a complex with Ag^+^ and is selectively retained on the bed, while the corresponding *cis*-cyclooctene binds weakly to Ag^+^ and elutes back to the reaction flask, where it is photoirradiated again. For *trans*-for-*cis* isomerization of our compounds, we used a micro-flow setup, since the short characteristic inner diameter of the microreactor allows a high overall absorption even at larger concentration which increases the gross conversion rate largely and reduces the reaction time from hours to minutes for typical photo-flow processes.^14^ In addition, process scale-up is facilitated by the numbering-up of several flow microcapillaries with almost identical performance.^15^ Two microreactors coiled around the UV lamp were used in parallel, and flow was adjusted to result in 2 to 3 min irradiation time (Scheme 2 and ESI†). Although Royzen *et al.* used 8 lamps of 35 W (light intensity: 12 800 μW cm^–2^),^13^ a single UV lamp of 10 W (light intensity: 21–24 μW cm^–2^) provided sufficient power for the isomerization reaction in our micro-flow setup. In-flow separation process based on Ag^+^ complexation was also optimized, by using several packed beds made of AgNO_3_-impregnated silica gel and glass beads. During experiment, flow was switched after 30–90 min from one packed bed to the next, in order to avoid saturation. After experiment, a NH_4_OH solution was used to liberate the *trans*-compound. With this optimized method using microreactors, 85% conversion can be achieved for *trans*-for-*cis* isomerization of cyclooctenol in 3 h (ESI†), compared to a reported 73% in 8 h or 70% in 3 h for non-microfluidic productions.^13,16^ For the new functionalized cyclooctene derivatives, photoisomerization yields reached 76% for a 6 h experiment, with fluoro-compound **3**. For sulfonate precursors, only mesylate **11b** and **20** could be isolated after isomerization, but **11b** was quite unstable (data not shown).

### Reactivity and stability of new dienophiles

Rate constants for reaction between new dienophiles and 3,6-di(pyridin-2-yl)-1,2,4,5-tetrazine in MeOH at 25 °C were determined by UV-spectrophotometry at 290 nm under pseudo-first order conditions (Fig. S2†). Compounds **10a** and **10b** react with the tetrazine with rate constants of 476 ± 33 M^–1^ s^–1^ and 1913 ± 196 M^–1^ s^–1^ respectively. Compared to *trans*-cyclooctenol rate constants (392 ± 6 M^–1^ s^–1^ for major isomer and 300 ± 22 M^–1^ s^–1^ for minor isomer), the presence of the PEG chain on **10a** and **10b** does not reduce the reactivity. Reaction rate of **19** with the tetrazine is also fast, with a rate constant of 1620 ± 149 M^–1^ s^–1^. These rate constants are comparable to the ones reported in literature for this type of dienophiles.^6a,12^ While the new dienophiles are reactive toward a tetrazine, they also display excellent stability. Indeed, fluoro-*trans*-derivatives **2a**, **2b** and **3** are stable in phosphate-buffered saline (PBS, pH 7.4) at 37 °C for 19 h at least, as determined by HPLC (Tables S3, S5 and S7†).

### Radiolabeling with fluorine-18

In view of the limited stability of precursors for radiofluorination **11a** and **11b**, we decided to continue the project with dioxolane-fused *trans*-cyclooctene compounds only. Semi-automated radiosynthesis of **[^18^F]3** was performed on a home-made module. Nucleophilic substitution on mesylate precursor **20** by reaction with dry K[^18^F]F, K_222_ complex was achieved in MeCN at 90 °C for 15 min. During radiosynthesis of **[^18^F]3**, less than 1% of *cis*-compound **[^18^F]18** was generated. After purification by HPLC, **[^18^F]3** was obtained in 60 min, with 12% radiochemical yield (decay-corrected), a radiochemical purity >99% (Fig. S4†), and a specific activity of 70–188 GBq μmol^–1^.

### *In vitro* stability of **[^18/19^F]3**

In PBS (pH 7.4), **[^18^F]3** was rather stable, with 94% of intact compound after 2 h incubation at 37 °C. In rat plasma, **[^18^F]3** slowly isomerized into the corresponding *cis*-derivative **[^18^F]18**, with 52% and 34% of intact *trans*-compound after 1 h and 2 h incubation at 37 °C respectively (Fig. S5†). For pretargeted PET imaging, this slow degradation is not an issue, as the IEDDA reaction takes place in a few seconds and plasma clearance is expected to be relatively fast (<30 min). Additional experiments were performed to investigate the cause(s) of the isomerization,^7b,12^ and the presence of a thiol (2-mercaptoethanol) or temperature were found to promote *cis*-for-*trans* isomerization of **3** (Table S8 and Fig. S3†).

### Biodistribution of radioactivity after injection of **[^18^F]3**

**Fig. 2:**
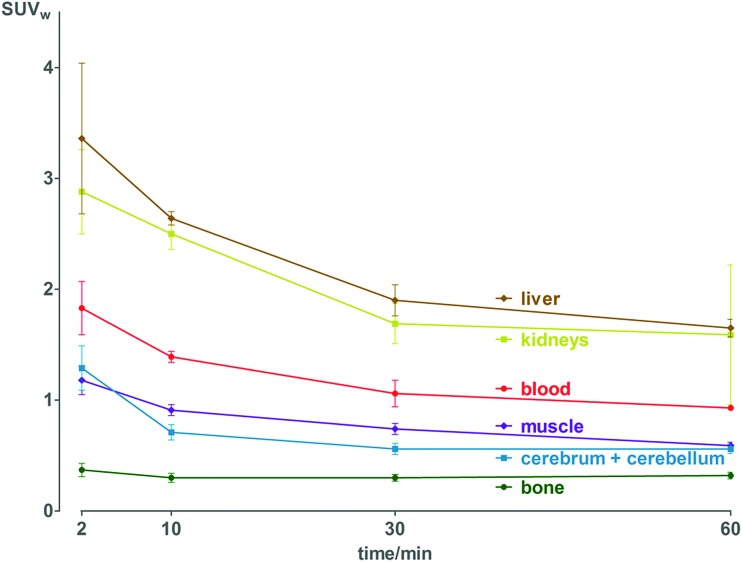
Biodistribution of radioactivity after injection of **[^18^F]3** in healthy NMRI mice in selected organs and fluids. Data are expressed as standardized uptake value (SUV_w_).

Pharmacokinetic profile of **[^18^F]3** was evaluated *in vivo*, in healthy NMRI mice, from 2 to 60 min p.i. (Fig. 2 and Table S11†). Results demonstrate absence of *in vivo* defluorination, as no significant bone uptake was observed at 60 min p.i. (0.3 ± 0.0 SUV_w_). Radioactivity was cleared *via* urinary and hepatobiliary systems. Interestingly, brain uptake was observed (1.3 ± 0.2 to 0.6 ± 0.0 SUV_w_ from 2 to 60 min p.i.). Analysis of brain and biofluids by HPLC after 15 min p.i. revealed 20.8 ± 1.1% of intact **[^18^F]3** in brain, 5.9 ± 0.6% in plasma and 0.1 ± 0.0% in urine (Fig. S6†), indicating fast metabolism.

### *In vitro* pretargeting experiments using **[^18^F]3**

**Scheme 3:**
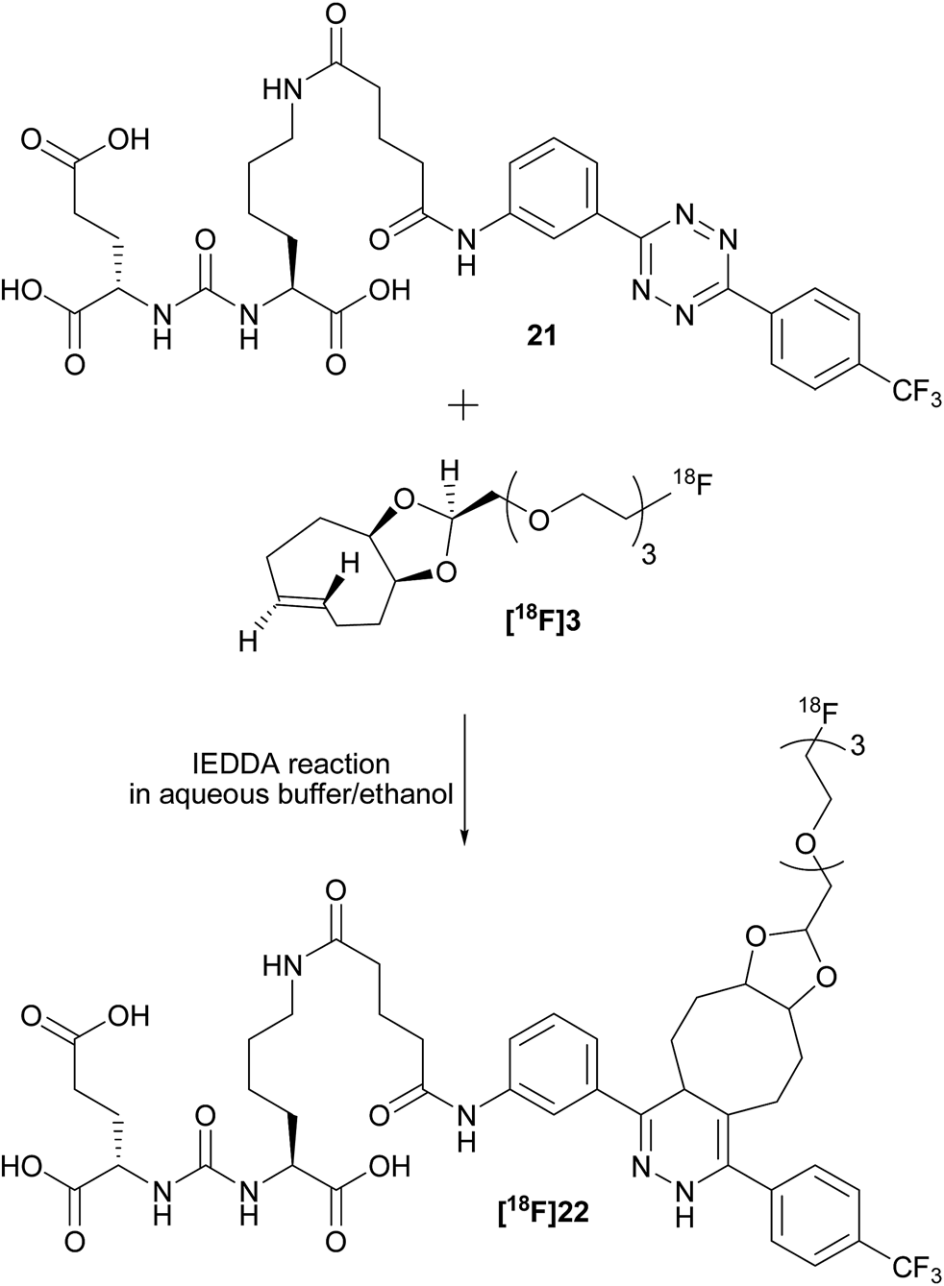
Reaction between dienophile **[^18^F]3** and tetrazine **21** leads to **[^18^F]22** (mixture of isomers) quantitatively in a few seconds.

To check the usefulness of **[^18^F]3** as a new dienophile for IEDDA reactions and for pretargeted PET imaging, *in vitro* and *in vivo* experiments were performed using a pseudo peptide with affinity for prostate-specific membrane antigen (PSMA)^17^ conjugated to 3-(4-(trifluoromethyl)phenyl)-6-phenyl-1,2,4,5-tetrazine^10*f*^ (**21**, Scheme 3, synthesis described in ESI†).

**Fig. 3:**
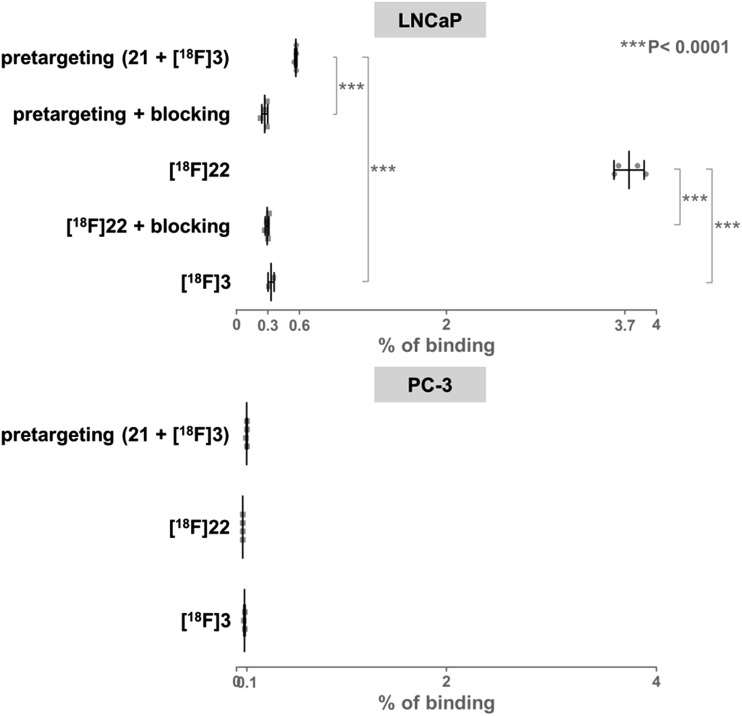
Percentage of binding of radioactivity to LNCaP tumor slices after a pretargeting experiment with successive addition of **21** and **[^18^F]3** or after incubation with **[^18^F]22** was significantly higher than in control experiments.

For *in vitro* experiments, prostate tumor slices (LNCaP and PC-3 cells) were incubated with **21**, washed, and **[^18^F]3** was added. Direct incubation with “preclicked”-compound **[^18^F]22** (Scheme 3, Fig. S7†) was also performed. To check the specificity of the approach, blocking experiments with the non-structural related inhibitor 2-(phosphonomethyl)pentane-1,5-dioic acid^18^ and incubation with **[^18^F]3** only were also carried out. The fraction of bound activity was determined after autoradiography (Fig. 3 and S9†). Significant PSMA-specific binding to LNCaP tumor slices (expressing PSMA receptors) was observed in the pretargeting experiment, but it was lower than the specific binding of “preclicked”-compound **[^18^F]22**. In PC-3 cells (negative control), no significant binding was observed.

### Proof of principle *in vivo* PET imaging

**Fig. 4:**
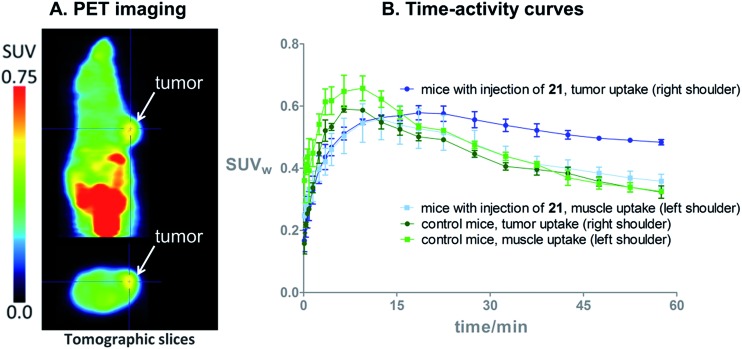
(A) *In vivo* PET images 2 h after i.v. injection of bioorthogonal **[^18^F]3** (11 MBq), in a mouse bearing an LNCaP tumor treated with tetrazine **21** (50 μg). (B) Time–activity curves from 0 to 60 min after i.v. injection of **[^18^F]3**, in LNCaP tumor-bearing mice with (*N* = 3) or without (=control, *N* = 3) intratumoral injection of **21**.

**Fig. 5:**
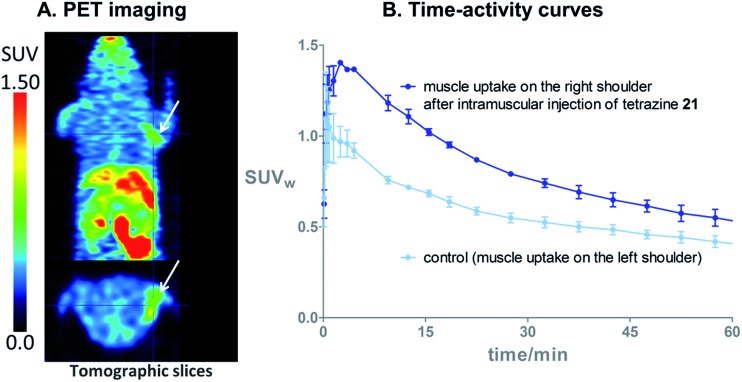
(A) *In vivo* PET images 60 min after i.v. injection of **[^18^F]3** (11 MBq) in an healthy NMRI mouse which received an intramuscular injection of tetrazine **21** (50 μg) in the muscle on the right shoulder (arrows), and saline with 10% dimethyl sulfoxide in the muscle on the left shoulder. (B) Time–activity curves from 0 to 60 min after i.v. injection of **[^18^F]3**, in healthy NMRI mice (*N* = 3). The uptake of radioactivity in the tetrazine-enriched tissue is significantly higher than in the tissue which received only the vehicle, from 3 min to 47 min p.i. of **[^18^F]3**.

On the basis of these promising results, proof of principle PET imaging experiments were conducted in LNCaP prostate tumor-bearing mice. Compound **21** (50 μg) was administered intratumorally 10 min before intravenous (i.v.) injection of **[^18^F]3**, following a similar protocol reported by Emmetiere *et al.*,^7*f*^ to avoid variability due to tetrazine concentration in target tissues. Dynamic (0–60 min p.i.) and static (120 min p.i.) microPET scans were acquired. PET imaging with **[^18^F]3** allowed visualization of the tumor, as shown in Fig. 4. Moreover, tumor uptake was significantly higher than muscle uptake, as early as 30 min p.i. and up to 2 h p.i. In order to ensure that the tumor accumulation was due to the ^18^F-labeled conjugate formed by the IEDDA reaction, control experiments were also performed in mice that received only i.v. injection of **[^18^F]3**. Uptake of radioactivity in the tumor was significantly lower than in tetrazine-**21**-preinjected tumors, from 30 min to 2 h p.i. (Fig. 4 and S10†). No significant difference was observed between muscle uptake and tumor uptake in control experiments. According to another PET imaging experiment (Fig. 5 and S11†), the accumulation of radioactivity in tetrazine-enriched tissues is not an effect due to the injection of the tetrazine, as the injection of the same volume of vehicle alone (saline with 10% dimethyl sulfoxide) does not lead to a significant uptake of radioactivity.

These proof of principle experiments demonstrate the usefulness of **[^18^F]3** for the *in vivo* IEDDA reaction. In order to successfully apply this new dienophile to pretargeted immunoPET, stability will be favored over reactivity for the choice of the tetrazine, as a tetrazine-derivatised antibody will be injected several days before the radiolabeled dienophile. A tetrazine such as reported by Selvaraj *et al.*,^10*f*^ or by Karver *et al.*,^7*c*^ possibly modified with a PEG linker, might be a good choice.

## Conclusions

In summary, we developed three new dienophiles for IEDDA reactions, and compound **3** was selected for pretargeting applications. *trans*-**3** has been prepared *via* diastereoselective synthesis, and the *trans*-for-*cis* isomerization step has been performed by micro-flow photochemistry with 76% yield. The new microfluidic setup reported here can be applied as continuous process which is promising for process scale-up. ^18^F-radiolabeling of **3** can be carried out by nucleophilic substitution at high specific activity, in 60 min, with 12% radiochemical yield, and >99% radiochemical purity. *In vivo*, **[^18^F]3** demonstrated a suitable pharmacokinetic profile and no defluorination was observed. Proof of principle PET imaging experiments with **[^18^F]3**, on a prostate tumor model injected with a tetrazine-coupled PSMA antagonist 10 min before radiotracer injection, allowed clear visualization of the tumor tissue, due to the ^18^F-labeled conjugate formed by the IEDDA reaction. In conclusion, new dienophile **[^18^F]3** seems suitable for pretargeted PET imaging, although further structural modification can still be done to favor urinary excretion. In the future, **[^18^F]3** will be investigated for pretargeted immunoPET, by using a tetrazine-derivatised antibody.
