# Supplementary material for: Micro-flow photosynthesis of new dienophiles for inverse-electron-demand Diels–Alder reactions. Potential applications for pretargeted in vivo PET imaging
Source: Chem Sci. 2016 Oct 7;8(2):1251–8. doi: 10.1039/c6sc02933g (PMC5369547; doi:10.1039/c6sc02933g)
Supplement: Supplementary file 1 [file SC-008-C6SC02933G-s001.pdf]

Micro-Flow Photosynthesis of New Dienophiles for Inverse-Electron-Demand Diels-Alder Reactions. Potential applications for pretargeted *in vivo* PET imaging.

Emilie M.F. Billaud, Elnaz Shahbazali, Muneer Ahamed, Frederik Cleeren, Timothy Noël, Michel Koole, Alfons Verbruggen, Volker Hessel, Guy Bormans.

SUPPORTING INFORMATION

<u>Table of contents:</u>

1. Material and general methods S2

2. Syntheses S5

3. Photoisomerizations S15

4. Kinetics S21

5. Stability studies S24

6. Radiosyntheses S28

7. Stability studies of **[^{18}F]3** in phosphate-buffered saline (PBS) and rat plasma S29

8. Biodistribution after injection of **[^{18}F]3** in healthy mice and radiometabolite analyses S30

9. *In vitro* pretargeting S32

10. *In vivo* microPET imaging S35

11. ^{1}H-NMR and ^{13}C-NMR spectra of representative compounds S36

12. References S53

1. Material and general methods

Chemistry. All reagents and solvents were purchased from Sigma-Aldrich, Fisher Scientific, TCI or Acros Organics. Distilled water and ultrapure water (18.2 MΩ.cm at 25°C, 0.22 µm filtration) were used. Unless otherwise noted, moisture-sensitive reactions were conducted under dry nitrogen atmosphere. Thin layer chromatography (TLC) was performed on silica gel-based plates (silica gel on TLC aluminium foils, 60 Å, Sigma-Aldrich) and visualized with UV light (254 nm) or developed with potassium permanganate dyeing agent. Column chromatography was carried out on silica gel (0.060-0.200 mm, 60 Å, Acros Organics). Nuclear magnetic resonance (NMR) spectra (400 MHz for ^{1}H, 101 MHz for ^{13}C and 376 MHz for ^{19}F) and 2D-NMR (^{1}H-^{1}H COSY, ^{1}H-^{13}C HSQC) were recorded for each compound on a Bruker AVANCE II 400 Ultrashield instrument (Bruker), with chemical shift values expressed in ppm relative to TMS (δ_H 0.00 and δ_C 0.0) or residual chloroform (δ_H 7.26 and δ_C 77.2) as standard. High-resolution mass spectrometry (HRMS) was achieved using a Dionex Ultimate 3000 LC System (Thermo Fisher Scientific) coupled in series to an ultra-high resolution time-of-flight mass spectrometry (TOF-HRMS) (MaXis impact, Bruker), equipped with orthogonal electrospray ionization (ESI) interface.

Photochemistry. Photoisomerizations were carried out in collaboration with the Technische Universiteit Eindhoven, The Netherlands. Micro-photoisomerization reactions were performed using the setup represented in Figure S1. The setup consists of HPLC pump (Smartline Pump 1050, Knauer), capillary tube made of fluorinated ethylene propylene (FEP tubing 1548L, ID 0.02”, OD 1/16”, IDEX), low pressure amalgam lamp (H10W-212, Dinies Technologies GmbH), safety oven (Carbolite) and tubular packed bed columns (stainless steel, ID 0.18”, OD 1/4”, L 25 or 52 mm, Swagelok). The solution was pumped from the inlet solution flask that was stirred with magnetic stirrer to micro-photoreactors (FEP capillary tube). Micro-photoreactors were wound around the UV lamp placed in the safety oven. The outlet from the photoreactors was then entered into the packed bed columns which were packed with silver nitrate impregnated-silica gel (~10 wt. % loading, +230 mesh, Sigma-Aldrich) mixed with 1 mm and 212-300 µm borosilicate glass beads (Sigma-Aldrich). The inert glass beads were used to avoid particle agglomeration which leads to channeling of the flow inside the packed bed column. The inlet and outlet of the packed bed attached frits with a pore size of 10 µm to maintain the packing material inside the column. The outlet of the packed bed columns was recycled back to the inlet solution flask through a FEP capillary tube (L 0.2 m). Semi-preparative HPLC purification was performed on a VWR-Hitachi LaChrom Elite system equipped with an L2130 pump and an L2400 UV detector.

Kinetics. Measurements were recorded on a UV-1800 spectrophotometer (Shimadzu), using a Quartz Suprasil high precision cell (Hellma Analytics (10 mm light path)).

Radiochemistry. No-carrier-added fluorine-18 (half-life: 109.8 min) was produced *via* an [^{18}O(p, n)^{18}F] nuclear reaction by irradiation of a H_2[^{18}O]O target on an IBA Cyclone 18/9 cyclotron (18 MeV proton beam, Ion Beam Applications). Radiosyntheses and semi-preparative HPLC were semi-automated on a home-made module. SepPak QMA and C18 cartridges were purchased from Waters. Analytical HPLC was performed either on a VWR-Hitachi LaChrom Elite system equipped with an L2130 pump, an L2400 UV detector and a Gabi Star detector (Raytest) or on a Waters system equipped with an Alliance 2695 separation module (autosampler + pump + degasser + oven), a 2996 Photodiode Array Detector and a Gabi Star radiometric detector (Raytest). Unless otherwise indicated, analyses were performed at 210 nm. A delay time of 0.15 min was observed between the first detector (UV) and the second detector (radiometric). Purity and specific activity were determined by analytical HPLC. For each biologically tested compound, radiochemical purity was over 98%. The identity of each radiofluorinated compound was confirmed by radio-HPLC by co-elution with its non-radioactive counterpart.

Octanol−Water Distribution Coefficient (log D). Approximately 100 kBq of radiotracer was added to a mixture of PBS (pH 7.4) saturated in *n*-octanol (1.0 mL) and *n*-octanol saturated in PBS (1.0 mL) in an Eppendorf microcentrifuge tube. The mixture was vortexed three times for 1 min, at RT. After

centrifugation (3000 rpm, 5 min), aliquots (100 µL) of both layers were sampled and the radioactivity was measured using an automatic gamma-counter (Perkin Elmer 2480 Wizard^2). Counts were corrected for background radiation, physical decay and counter dead time. The experiments were performed in triplicate.

Stability studies. Analytical HPLC was performed either on a VWR-Hitachi LaChrom Elite system equipped with an L2130 pump, an L2400 UV detector and a Gabi Star radiometric detector (Raytest) or on a Merck Hitachi LaChrom system equipped with an L7100 pump, an L4250 UV detector and a Gabi Star radiometric detector (Raytest). Rat plasma was obtained from healthy female Wistar rats RjHan:WI or Crl:WI(Han) (body mass: 200 and 300 g), ordered from Janvier or from Charles River. Rats were sacrificed by decapitation under anesthesia (2.5% isoflurane in O_2 at 1 L.min^{-1} flow rate), and blood was collected in EDTA-containing tubes (4 mL tubes; BD vacutainer, Beckton Dickinson). Next, the blood was centrifuged (3000 rpm, 10 min) to separate the plasma.

Biodistribution studies in healthy mice and radiometabolite analyses. All animal experiments were conducted according to the Belgian code of practice for the care and the use of animals, after approval from the University Ethical Committee for animals (Project P159-2015). Animals were housed in individually ventilated cages in a thermo-regulated, humidity-controlled facility under a 12 h-12 h light-dark cycle, with access to food and water *ad libitum*. Biodistribution studies were carried out in healthy male Naval Medical Research Institute (NMRI) mice (body mass: 40-60 g, 5-7 weeks old) obtained from Janvier or Envigo. Mice under anesthesia (2.5% isoflurane in O_2 at 1 L.min^{-1} flow rate) received an i.v. injection of **[^{18}F]3** (~1.1 MBq) *via* a tail vein. Radioactivity injected to each mouse was determined by measuring the activity in the syringe before and after injection (Capintec Radioisotope Dose Calibrator CRC-15PET). At selected time points (2, 10, 30 or 60 min postinjection (p.i.)), mice were sacrificed by decapitation (N = 3/time point). Blood and major organs were promptly collected and weighed, and their radioactivity was counted using an automatic gamma-counter. For calculation of total radioactivity in blood, bone and muscle, tissue mass was assumed to be, respectively, 7, 12 and 40% of the total body mass.

For radiometabolite analysis of plasma and urine, healthy NMRI mice under anesthesia (2.5% isoflurane in O_2 at 1 L.min^{-1} flow rate) received an i.v. injection of **[^{18}F]3** (~9 MBq) *via* a tail vein. After 15 min p.i., mice were sacrificed by decapitation (N = 3). Blood was collected in EDTA-containing tubes and centrifuged (3000 rpm, 10 min) to separate the plasma. A sample (100 µL) was taken from the plasma and added to acetonitrile (100 µL). After stirring and centrifugation (3000 rpm, 5 min), the supernatant was removed, filtered (0.22 µm), and analyzed by HPLC (Waters Xbridge C18, 5 µm, 4.6 mm x 150 mm; flow rate: 1.0 mL.min^{-1}; UV detection: 210 nm; mobile phase: A: Na_2HPO_4, 0.025 M, pH 8.5, B: EtOH; 10% to 80% B in 20 min (linear gradient) then isocratic elution of 80% B for 5 min). Urine was directly analyzed by HPLC using the same conditions.

For radiometabolite analysis of brain, healthy NMRI mice under anesthesia (2.5% isoflurane in O_2 at 1 L.min^{-1} flow rate) received an i.v. injection of **[^{18}F]3** (~9 MBq) *via* a tail vein. After 15 min p.i., mice were sacrificed by administering an overdose of Nembutal (CEVA Santé Animale, 200 mg.kg^{-1} intraperitoneal). When breathing has stopped, the mice were perfused with saline (5 mL). Brain was isolated and homogenized in acetonitrile (2 mL) for about 2 min, at 0 °C. After centrifugation (3000 rpm, 5 min), the supernatant was analyzed by HPLC (Waters Xbridge C18, 5 µm, 4.6 mm x 150 mm; flow rate: 1.0 mL.min^{-1}; UV detection: 210 nm; mobile phase: A: Na_2HPO_4, 0.025 M, pH 8.5, B: EtOH; 10% to 80% B in 20 min (linear gradient) then isocratic elution of 80% B for 5 min).

In vitro pretargeting. Prostate tumor (LNCaP and PC-3 cells) frontal, horizontal or sagital sections (20 µm) were obtained using a cryotome (Shandon Cryotome FSE, Thermo Fisher Scientific), and mounted on adhesive microscope slides (Superfrost Plus, Thermo Fisher Scientific). The slices were cut at a temperature between -15 °C and -18 °C. The slides were stored at -20 °C. Autoradiography was

performed using storage phosphor screens (super-resolution screen, Perkin Elmer). Screens were read in a Cyclone Plus system (Perkin Elmer) and analysed using Optiquant software (Perkin Elmer).

In vivo microPET imaging in tumor-bearing mice 10×10^6 LNCaP cells in 50% matrigel were subcutaneously inoculated into the right shoulder of male BALB/c nu/nu mice (body mass: 20-25 g, 7-8 weeks old) obtained from Janvier. The tumors were allowed to grow for 4 weeks (tumoral volume range of 100-700 mm^3). For PET imaging experiments, compound **21** (50 µg/69 nmol/50 µL in saline with 10% dimethyl sulfoxide) was administered intratumorally 10 min before intravenous injection (tail vein) of **[^{18}F]3** (8-12 MBq/mouse) in mice under anesthesia (2.5% isoflurane in O_2 at 1 $L.min^{-1}$ flow rate). Control mice received only intravenous injection of **[^{18}F]3** (8-12 MBq/mouse). For each experiment, 3-4 mice were used. Whole-body dynamic 60-min microPET scans, starting immediately after **[^{18}F]3** injection, and whole-body static scans 120 min after **[^{18}F]3** injection (duration: 10 min) were performed on a Focus™ 220 microPET scanner (Siemens/Concorde Microsystems) on the LNCaP-bearing mice, which were kept under gas anesthesia during the whole procedure (2.5% isoflurane in O_2 at 1 $L.min^{-1}$ flow rate). Data were acquired in list mode with an energy window of 350-650 keV and a coincidence timing window of 6 ns. Acquisition data from dynamic scans were Fourier rebinned in 21 time frames (4 x 15 s, 4 x 60 s, 5 x 180 s, 8 x 300 s). For both dynamic and static scans, images were reconstructed using maximum a posteriori iterative reconstruction (MAP) with a zoom of 2 and a 128x128x95 matrix resulting in a pixel size of 0.949 mm and a slice thickness of 0.796 mm. Data were corrected for attenuation, scanner normalization, radiation scatter and randoms. Tumors were manually delineated to comprise all tumor tissue while as reference muscle tissue from the contralateral frontal limb was used. Based on these delineations, time-activity curves were extracted from the dynamic PET data.

In vivo microPET imaging in healthy mice NMRI mice (body mass: 40-60 g, 5-7 weeks old, N = 3) under anesthesia (2.5% isoflurane in O_2 at 1 $L.min^{-1}$ flow rate) received an intramuscular injection of compound **21** (50 µg/69 nmol/50 µL in saline with 10% dimethyl sulfoxide) in the muscle on the right shoulder, and an intramuscular injection of saline with 10% dimethyl sulfoxide in the muscle on the left shoulder. 5 min after, **[^{18}F]3** (10-12 MBq/mouse) was administered by intravenous injection (tail vein). Whole-body dynamic 60-min microPET scans, starting immediately after **[^{18}F]3** injection, and whole-body static scans 120 min after **[^{18}F]3** injection (duration: 10 min) were performed on the healthy mice, which were kept under gas anesthesia during the whole procedure (2.5% isoflurane in O_2 at 1 $L.min^{-1}$ flow rate). Data were acquired in list mode with an energy window of 350-650 keV and a coincidence timing window of 6 ns. Acquisition data from dynamic scans were Fourier rebinned in 21 time frames (4 x 15 s, 4 x 60 s, 5 x 180 s, 8 x 300 s). For both dynamic and static scans, images were reconstructed using maximum a posteriori iterative reconstruction (MAP) with a zoom of 2 and a 128x128x95 matrix resulting in a pixel size of 0.949 mm and a slice thickness of 0.796 mm. Data were corrected for attenuation, scanner normalization, radiation scatter and randoms. Muscles on the right and left shoulders were manually delineated. Based on these delineations, time-activity curves (TACs) were extracted from the dynamic PET data.

Statistical analysis Quantitative data are expressed as mean ± standard error of the mean (SEM). Means were compared using unpaired one-tailed Student *t*-test. Values were considered statistically significant for $P < 0.05$.

2. Syntheses

2.1 Synthesis of (*Z*)-5-(2-(2-(2-(2-fluoroethoxy)ethoxy)ethoxy)ethoxy)cyclooct-1-ene (**9**) and 2-(2-(2-(2-((*Z*)-cyclooct-4-enyloxy)ethoxy)ethoxy)ethoxy)ethyl methanesulfonate (**8**)

3,3-diisopropyl-2-methyl-4,7,10,13-tetraoxa-3-silapentadecan-15-ol (23)

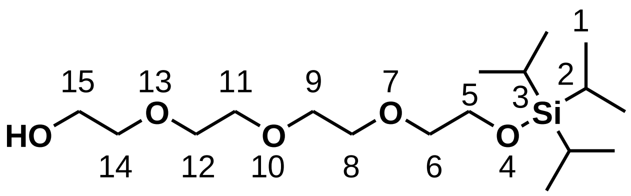

A solution of sodium hydride (60% in mineral oil; 386 mg; 9.65 mmol; 1.25 eq.) in anhydrous tetrahydrofuran (20 mL) was stirred at RT for 5 min under N_2 atmosphere. Tetraethylene glycol (2.67 mL; 15.45 mmol; 2 eq.) was added dropwise at 0 °C and the reaction mixture was stirred for 5 more min before addition of triisopropylsilyl chloride (1.65 mL; 7.72 mmol; 1 eq.). The resulting mixture was stirred at RT for 1 h and the reaction was stopped by addition of water (30 mL). The mixture was extracted with ethyl acetate (3 x 30 mL). The organic layers were combined, washed with brine, dried over magnesium sulfate, filtered and evaporated under reduced pressure. The crude product was purified by column chromatography (ethyl acetate/*n*-heptane, 75:25) to yield **23** (1.91 g; 5.45 mmol; 71%) as a colorless oil. Rf on TLC (ethyl acetate/*n*-heptane 75:25 v/v) 0.39; ^{1}H NMR ($CDCl_3$) 1.02-1.09 (21H, m, CH_3-1, H-2), 2.62 (1H, br s, OH), 3.54-3.58 (4H, m, CH_2-6, 14), 3.62-3.63 (8H, m, CH_2-8, 9, 11, 12), 3.67-3.69 (2H, m, CH_2-15), 3.81 (2H, t, J = 5.6 Hz, CH_2-5); ^{13}C NMR ($CDCl_3$) 12.1 (3C, C-2), 18.0 (6C, C-1), 61.8 (C-15), 63.1 (C-5), 70.5 (C-8 or 9 or 11 or 12), 70.8 (C-8 or 9 or 11 or 12), 70.8 (C-8 or 9 or 11 or 12), 70.9 (C-8 or 9 or 11 or 12), 72.6 (C-14 or 6), 72.9 (C-14 or 6); HRMS/ESI m/z $[M+H]^+$ = 351.2587 (calculated for $C_{17}H_{38}O_5Si$: 351.2561).

3,3-diisopropyl-2-methyl-4,7,10,13-tetraoxa-3-silapentadecan-15-yl methanesulfonate (4)

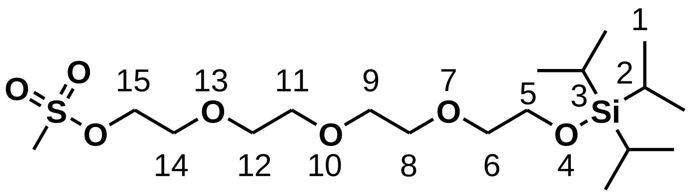

To a solution of compound **23** (1.45 g; 4.14 mmol), distilled *N*,*N*-diisopropylethylamine (865 µL; 4.96 mmol; 1.2 eq.), and 4-(dimethylamino)pyridine (51 mg; 0.41 mmol; 0.1 eq.) in dry dichloromethane (50 mL) was added mesyl chloride (384 µL; 4.96 mmol; 1.2 eq.) under N_2 atmosphere. The reaction mixture was stirred at RT for 1 h and then poured into a saturated aqueous solution of sodium hydrogencarbonate (50 mL). The organic layer was separated and the aqueous phase was extracted with dichloromethane (2 x 50 mL). The organic layers were combined, dried over magnesium sulfate, filtered and evaporated under reduced pressure. The crude product was purified by column chromatography (dichloromethane/ethyl acetate 90:10) to yield **4** (1.65 g; 3.85 mmol; 93%) as a pale yellow oil. Rf on TLC (dichloromethane/ethyl acetate 90:10 v/v) 0.40; ^{1}H NMR ($CDCl_3$) 1.02-1.11 (21H, m, CH_3-1, H-2), 3.04 (3H, s, CH_3-S), 3.56 (2H, t, J = 5.5 Hz, CH_2-6), 3.60-3.65 (8H, m, CH_2-8, 9, 11, 12), 3.74-3.76 (2H, m, CH_2-14), 3.82 (2H, t, J = 5.5 Hz, CH_2-5), 4.34-4.36 (2H, m, CH_2-15); ^{13}C NMR ($CDCl_3$) 12.1 (3C, C-2), 18.1 (6C, C-1), 37.8 (CH_3-S), 63.1 (C-5), 69.2 (C-14 or 15), 69.4 (C-14 or 15), 70.7 (C-8

or 9 or 11 or 12), 70.8 (2C, C-8 or 9 or 11 or 12), 70.9 (C-8 or 9 or 11 or 12), 72.9 (C-6); HRMS/ESI m/z $[M+H]^+$ = 429.2386 (calculated for $C_{18}H_{40}O_7SSi$: 429.2337).

(*Z*)-15-(cyclooct-4-en-1-yloxy)-3,3-diisopropyl-2-methyl-4,7,10,13-tetraoxa-3-silapentadecane (5)

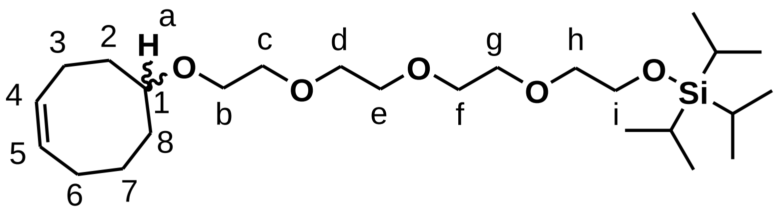

A dispersion of sodium hydride (60% in mineral oil; 396 mg; 9.91 mmol) in anhydrous tetrahydrofuran (10 mL) was stirred at RT for 5 min under N_2 atmosphere. 5-Hydroxy-1-cyclooctene (500 mg; 3.96 mmol) in anhydrous tetrahydrofuran (10 mL) was added dropwise and the reaction mixture was stirred for 45 min at 45 °C under N_2 atmosphere. Compound **4** (1.40 g; 3.27 mmol) in anhydrous tetrahydrofuran (10 mL) was then added and the resulting mixture was stirred at 45 °C overnight under N_2 atmosphere. Back to RT, the reaction was stopped by addition of water (50 mL). The mixture was extracted with ethyl acetate (3 x 50 mL). The organic layers were combined, dried over magnesium sulfate, filtered and evaporated under reduced pressure. The crude product was purified by column chromatography (dichloromethane/ethyl acetate, 100:0 → 80:20) to yield **5** (960 mg; 2.09 mmol; 64%) as a colorless oil. Rf on TLC (dichloromethane/ethyl acetate 90:10 v/v) 0.39; ^1H NMR ($CDCl_3$) 1.04-1.11 (21H, m, CH_3, CH-Si), 1.34-1.43 (1H, m, H-7), 1.45-1.54 (1H, m, H-2), 1.63-1.85 (3H, m, H-7, H-8, H-8), 1.89-1.97 (1H, m, H-2), 1.98-2.06 (1H, m, H-3), 2.08-2.16 (2H, m, H-6, H-6), 2.28-2.38 (1H, m, H-3), 3.33-3.38 (1H, m, H-a), 3.48-3.67 (14H, m, CH_2-b, c, d, e, f, g, h), 3.83 (2H, t, *J* = 5.6 Hz, CH_2-i), 5.53-5.68 (2H, m, H-4, H-5); ^{13}C NMR ($CDCl_3$) 12.2 (3C, C-Si), 18.1 (6C, CH_3), 22.9 (C-3), 25.7 (C-6 or 7), 26.0 (C-6 or 7), 33.5 (C-8), 34.3 (C-2), 63.1 (C-i), 67.9 (C-b), 70.8 (C-c, d, e, f or g), 70.8 (C-c, d, e, f or g), 70.9 (C-c, d, e, f or g), 71.0 (C-c, d, e, f or g), 71.1 (C-c, d, e, f or g), 72.9 (C-h), 81.1 (C-1), 129.5 (C-4 or 5), 130.2 (C-4 or 5); HRMS/ESI m/z $[M+H]^+$ = 459.3514 (calculated for $C_{25}H_{50}O_5Si$: 459.3500).

(*Z*)-2-(2-(2-(2-(cyclooct-4-en-1-yloxy)ethoxy)ethoxy)ethoxy)ethanol (6)

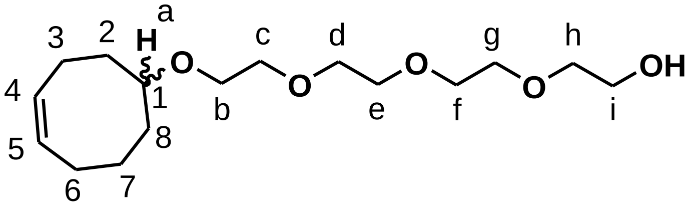

To a solution of compound **5** (800 mg; 1.74 mmol) in anhydrous tetrahydrofuran (20 mL) was added a solution of tetrabutylammonium fluoride 1 M in tetrahydrofuran (2.62 mL; 2.62 mmol; 1.5 eq.). The mixture was stirred at RT for 2 h. The reaction was quenched by addition of a saturated aqueous solution of sodium hydrogencarbonate (50 mL), followed by water (25 mL) and ethyl acetate (30 mL). The organic layer was separated and the aqueous phase was extracted with ethyl acetate (2 x 30 mL). The organic layers were combined, dried over magnesium sulfate, filtered and evaporated under reduced pressure. The crude product was purified by column chromatography (ethyl acetate/ethanol, 95:05) to yield **6** (465 mg; 1.54 mmol; 89%) as a colorless oil. Rf on TLC (ethyl acetate/ethanol, 95:05) 0.41; ^1H NMR ($CDCl_3$) 1.30-1.44 (1H, m, H-7), 1.45-1.55 (1H, m, H-2), 1.64-1.85 (3H, m, H-7, H-8, H-8), 1.89-1.97 (1H, m, H-2), 1.99-2.07 (1H, m, H-3), 2.08-2.17 (2H, m, H-6, H-6), 2.29-2.38 (1H, m, H-3), 2.66 (1H, br s, OH), 3.34-3.39 (1H, m, H-a), 3.50-3.66 (14H, m, CH_2-b, c, d, e, f, g, h), 3.71-3.72 (2H, m, CH_2-i), 5.54-5.69 (2H, m, H-4, H-5); ^{13}C NMR ($CDCl_3$) 22.9 (C-3), 25.7 (C-7), 26.0 (C-6), 33.5 (C-8), 34.3 (C-

2), 62.0 (C-i), 67.9 (C-b), 70.6 (C-c, d, e, f or g), 70.8 (3C, C-c, d, e, f or g), 71.1 (C-c, d, e, f or g), 72.7 (C-h), 81.2 (C-1), 129.6 (C-4 or 5), 130.2 (C-4 or 5); HRMS/ESI m/z $[M+H]^+$ = 303.2183 (calculated for $C_{16}H_{30}O_5$: 303.2166).

(*Z*)-2-(2-(2-(2-(cyclooct-4-en-1-yloxy)ethoxy)ethoxy)ethoxy)ethyl 4-methylbenzenesulfonate (7)

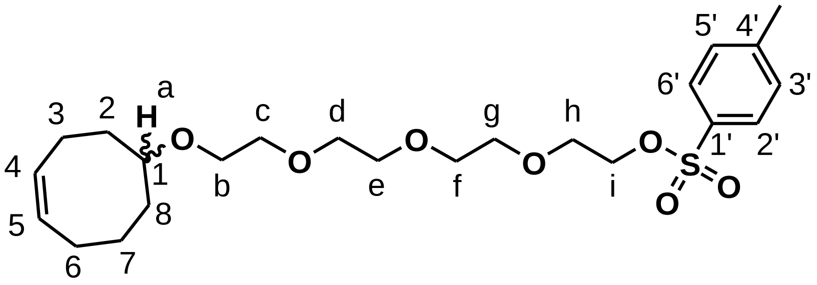

To a solution of compound **6** (270 mg; 0.89 mmol), distilled triethylamine (299 µL; 2.14 mmol; 2.4 eq.), and 4-(dimethylamino)pyridine (22 mg; 0.18 mmol; 0.2 eq.) in dry dichloromethane (10 mL) was added tosyl chloride (204 mg; 1.07 mmol; 1.2 eq.) under N_2 atmosphere. The reaction mixture was stirred at RT for 2 h. The mixture was poured into a saturated aqueous solution of sodium hydrogencarbonate (20 mL). After extraction with dichloromethane (3 x 20 mL), the organic layers were combined, dried over magnesium sulfate, filtered and evaporated under reduced pressure. The crude product was purified by column chromatography (ethyl acetate/*n*-heptane 5:5) to yield **7** (388 mg; 0.85 mmol; 96%) as a colorless oil. Rf on TLC (ethyl acetate/*n*-heptane 5:5) 0.33; ^{1}H NMR ($CDCl_3$) 1.33-1.43 (1H, m, H-7), 1.44-1.53 (1H, m, H-2), 1.63-1.84 (3H, m, H-7, H-8, H-8), 1.88-1.96 (1H, m, H-2), 1.98-2.07 (1H, m, H-3), 2.09-2.15 (2H, m, H-6, H-6), 2.28-2.37 (1H, m, H-3), 2.44 (3H, s, CH_3), 3.32-3.37 (1H, m, H-a), 3.50-3.63 (12H, m, CH_2-b, c, d, e, f, g), 3.68 (2H, t, *J* = 4.8 Hz, CH_2-h), 4.15 (2H, t, *J* = 4.8 Hz, CH_2-i), 5.54-5.68 (2H, m, H-4, H-5), 7.33 (2H, d, *J* = 8.3 Hz, H-3', H-5'), 7.79 (2H, d, *J* = 8.3 Hz, H-2', H-6'); ^{13}C NMR ($CDCl_3$) 21.7 (CH_3), 22.7 (C-3), 25.7 (C-7), 25.9 (C-6), 33.5 (C-8), 34.2 (C-2), 67.7 (C-b), 68.7 (C-h), 69.3 (C-i), 70.6 (C-c, d, e, f or g), 70.7 (C-c, d, e, f or g), 70.7 (C-c, d, e, f or g), 70.8 (C-c, d, e, f or g), 70.9 (C-c, d, e, f or g), 81.0 (C-1), 128.0 (2C, C-2', 6'), 129.5 (C-4 or 5), 129.9 (2C, C-3', 5'), 130.1 (C-4 or 5), 133.1 (C-1'), 144.9 (C-4'); HRMS/ESI m/z $[M+H]^+$ = 457.2283 (calculated for $C_{23}H_{36}O_7S$: 457.2255).

(*Z*)-5-(2-(2-(2-(2-fluoroethoxy)ethoxy)ethoxy)ethoxy)cyclooct-1-ene (9)

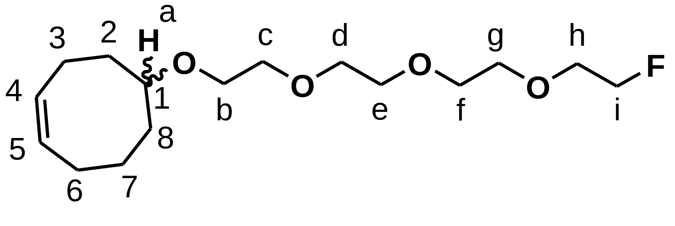

To a solution of compound **7** (115 mg; 0.25 mmol) in dry tetrahydrofuran (1.5 mL) was added a solution of tetrabutylammonium fluoride 1 M in tetrahydrofuran (1.26 mL; 1.26 mmol; 5 eq.) under N_2 atmosphere. The reaction mixture was heated at 80 °C for 3 h. Back to RT, the mixture was poured into a saturated aqueous solution of sodium hydrogencarbonate (20 mL). After extraction with dichloromethane (3 x 20 mL), the organic layers were combined, dried over magnesium sulfate, filtered and evaporated under reduced pressure. The crude product was purified by column chromatography (ethyl acetate/*n*-heptane 5:5 → 1:0) to yield **9** (72 mg; 0.24 mmol; 96%) as a colorless oil. Rf on TLC (ethyl acetate/*n*-heptane 5:5) 0.46; ^{1}H NMR ($CDCl_3$) 1.31-1.42 (1H, m, H-7), 1.43-1.51 (1H, m, H-2), 1.60-1.82 (3H, m, H-7, H-8, H-8), 1.87-1.95 (1H, m, H-2), 1.96-2.04 (1H, m, H-3), 2.07-2.13 (2H, m, H-6, H-6), 2.26-2.35 (1H, m, H-3), 3.30-3.36 (1H, m, H-a), 3.46-3.65 (12H, m, CH_2-b, c, d, e, f, g), 3.71 (2H, dt, *J* = 4.2 Hz, $^3J_{H-F}$ =

29.6 Hz, CH_2-h), 4.53 (2H, dt, J = 4.2 Hz, $^2J_{H-F}$ = 47.7 Hz, CH_2-i), 5.51-5.66 (2H, m, H-4, H-5); ^{13}C NMR ($CDCl_3$) 22.7 (C-3), 25.7 (C-7), 25.9 (C-6), 33.4 (C-8), 34.2 (C-2), 67.7 (C-b), 70.5 (d, $^2J_{C-F}$ = 20 Hz, C-h), 70.7 (2C, C-c, d, e, f or g), 70.7 (C-c, d, e, f or g), 70.9 (C-c, d, e, f or g), 70.9 (C-c, d, e, f or g), 81.0 (C-1), 83.2 (d, $^1J_{C-F}$ = 168 Hz, C-i), 129.5 (C-4 or 5), 130.1 (C-4 or 5); HRMS/ESI m/z $[M+H]^+$ = 305.2138 (calculated for $C_{16}H_{29}FO_4$: 305.2123).

2-(2-(2-(2-((*Z*)-cyclooct-4-enyloxy)ethoxy)ethoxy)ethoxy)ethyl methanesulfonate (8)

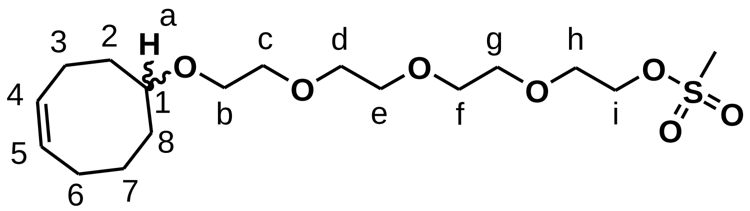

To a solution of compound **6** (100 mg; 0.33 mmol), distilled triethylamine (111 µL; 0.79 mmol; 2.4 eq.), and 4-(dimethylamino)pyridine (8 mg; 66 µmol; 0.2 eq.) in dry dichloromethane (10 mL) was added mesyl chloride (31 µL; 0.40 mmol; 1.2 eq.) under N_2 atmosphere. The reaction mixture was stirred at RT for 75 min. The mixture was poured into a saturated aqueous solution of sodium hydrogencarbonate (25 mL). After extraction with dichloromethane (3 x 20 mL), the organic layers were combined, dried over magnesium sulfate, filtered and evaporated under reduced pressure. The crude product was purified by column chromatography (ethyl acetate) to yield **8** (118 mg; 0.31 mmol; 94%) as a pale yellow oil. Rf on TLC (ethyl acetate) 0.57; ^{1}H NMR ($CDCl_3$) 1.34-1.45 (1H, m, H-7), 1.46-1.55 (1H, m, H-2), 1.64-1.85 (3H, m, H-7, H-8, H-8), 1.89-1.97 (1H, m, H-2), 1.99-2.08 (1H, m, H-3), 2.10-2.17 (2H, m, H-6, H-6), 2.29-2.38 (1H, m, H-3), 3.07 (3H, s, CH_3), 3.33-3.39 (1H, m, H-a), 3.49-3.68 (12H, m, CH_2-b, c, d, e, f, g), 3.75-3.77 (2H, m, CH_2-h), 4.36-4.38 (2H, m, CH_2-i), 5.54-5.69 (2H, m, H-4, H-5); ^{13}C NMR ($CDCl_3$) 22.7 (C-3), 25.7 (C-7), 25.9 (C-6), 33.4 (C-8), 34.2 (C-2), 37.8 (CH_3), 67.7 (C-b), 69.1 (C-h), 69.4 (C-i), 70.6 (C-c, d, e, f or g), 70.7 (2C, C-c, d, e, f or g), 70.7 (C-c, d, e, f or g), 71.0 (C-c, d, e, f or g), 81.0 (C-1), 129.5 (C-4 or 5), 130.1 (C-4 or 5); HRMS/ESI m/z $[M+H]^+$ = 381.1951 (calculated for $C_{17}H_{32}O_7S$: 381.1942).

2.2 Synthesis of (2*s*,3a*R*,9a*S*,*Z*)-2-((2-(2-(2-fluoroethoxy)ethoxy)ethoxy)methyl)-3a,4,5,8,9,9a-hexahydrocycloocta[*d*][1,3]dioxole (**18**) and 2-(2-(2-(((2*s*,3a*R*,9a*S*,*Z*)-3a,4,5,8,9,9a-hexahydrocycloocta[*d*][1,3]dioxol-2-yl)methoxy)ethoxy)ethoxy)ethyl methanesulfonate (**17**)

(*Z*,1*S*,2*R*)-cyclooct-5-ene-1,2-diol (12)

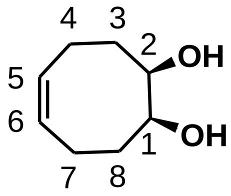

Synthesized according to a modified protocol from Gxoyiya *et al.*[1]

To a solution of 1,5-cyclooctadiene (6.22 mL; 50.7 mmol) in *tert*-butanol (15 mL) was added dropwise a solution of cetyltrimethylammonium permanganate[2] (20.45 g; 50.7 mmol) in water/*tert*-butanol (1:4; 75 mL). The mixture was stirred at RT for 4 h. The mixture was diluted with dichloromethane (100 mL) and with an aqueous solution of sodium hydroxide (5%, 100 mL), and then stirred for 15 min. The layers

were separated and the aqueous phase was extracted with dichloromethane (3 x 150 mL). The organic layers were combined, dried over magnesium sulfate, filtered and evaporated under reduced pressure. The crude product was purified by column chromatography (ethyl acetate) to yield **12** (1.22 g; 8.58 mmol; 17%) as a white solid. Rf on TLC (ethyl acetate) 0.37; ^{1}H NMR ($CDCl_3$) 1.76-1.85 (2H, m, H-3, H-8), 1.97-2.08 (4H, m, H-3, H-8, H-4, H-7), 2.14 (2H, s, OH), 2.47-2.54 (2H, m, H-4, H-7), 3.98-4.00 (2H, m, H-1, H-2), 5.65-5.67 (2H, m, H-5, H-6); ^{13}C NMR ($CDCl_3$) 23.1 (2C, C-4, 7), 32.1 (2C, C-3, 8), 75.2 (2C, C-1, 2), 130.1 (2C, C-5, 6).

2-(2-(2-(2-hydroxyethoxy)ethoxy)ethoxy)ethyl benzoate (24)

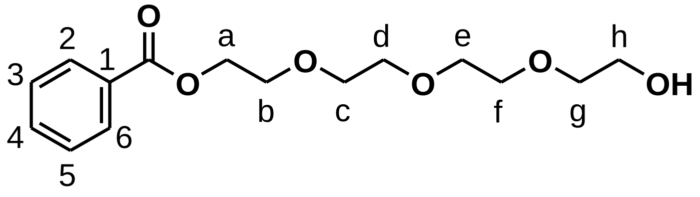

To a solution of tetraethylene glycol (3.93 mL; 22.77 mmol; 1.6 eq.) in anhydrous dichloromethane (120 mL) were successively added distilled *N*,*N*-diisopropylethylamine (3.72 mL; 21.34 mmol; 1.5 eq.) and 4-(dimethylamino)pyridine (174 mg; 1.42 mmol; 0.1 eq.) under N_2 atmosphere. Benzoyl chloride (2.00 g; 14.23 mmol) in anhydrous dichloromethane (15 mL) was then added dropwise and the mixture was stirred at RT overnight under N_2 atmosphere. The mixture was then poured into water (200 mL). The organic layer was separated and the aqueous phase was extracted with dichloromethane (3 x 100 mL). The organic layers were combined, dried over magnesium sulfate, filtered and evaporated under reduced pressure. The crude product was purified by column chromatography (ethyl acetate/dichloromethane/ethanol 67:29:4) to yield **24** (2.48 g; 8.31 mmol; 58%) as a pale yellow oil. Rf on TLC (ethyl acetate/dichloromethane/ethanol 67:29:4 v/v/v) 0.37; ^{1}H NMR ($CDCl_3$) 2.48 (1H, br s, OH), 3.57 (2H, m, CH_2-g), 3.64-3.71 (10H, m, CH_2-c, d, e, f, h), 3.83 (2H, t, J = 4.9 Hz, CH_2-b), 4.47 (2H, t, J = 4.9 Hz, CH_2-a), 7.40-7.44 (2H, m, H-3, 5), 7.52-7.56 (1H, m, H-4), 8.04-8.06 (2H, m, H-2, 6); ^{13}C NMR ($CDCl_3$) 61.9 (C-h), 64.2 (C-a), 69.4 (C-b), 70.5 (C-c or d or e or f), 70.8 (C-c or d or e or f), 70.8 (C-c or d or e or f), 70.9 (C-c or d or e or f), 72.6 (C-g), 128.4 (2C, C-3, 5), 129.8 (2C, C-2, 6), 130.3 (C-1), 133.1 (C-4), 166.7 (C=O); HRMS/ESI m/z $[M+H]^+$ = 299.1453 (calculated for $C_{15}H_{22}O_6$: 299.1489).

2-(2-(2-(2-oxoethoxy)ethoxy)ethoxy)ethyl benzoate (13)

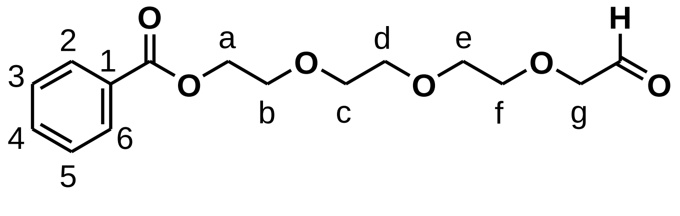

To a solution of compound **24** (300 mg; 1.01 mmol) in dichloromethane (15 mL) was added Dess-Martin periodinane (512 mg; 1.21 mmol; 1.2 eq.). The reaction mixture was stirred at RT for 2.5 h. The reaction was quenched with sodium thiosulfate (382 mg; 2.41 mmol; 2.4 eq.) in a saturated solution of sodium hydrogencarbonate (15 mL). The mixture was separated, and the aqueous phase was extracted with dichloromethane (2 x 25 mL). The organic layers were combined, dried over magnesium sulfate, filtered and evaporated under reduced pressure. The crude product was purified by column chromatography (ethyl acetate/dichloromethane 30:70 → 70:30) to yield **13** (196 mg; 0.66 mmol; 65%) as a colorless oil. Rf on TLC (ethyl acetate/dichloromethane 50:50 v/v) 0.33; ^{1}H NMR (acetone-d_6) 3.62-3.68 (8H, m, CH_2-c, d, e, f), 3.82-3.84 (2H, m, CH_2-b), 4.12 (2H, d, J = 0.8 Hz, CH_2-g), 4.44-4.46 (2H, m, CH_2-a), 7.49-7.53 (2H, m, H-3, 5), 7.63 (1H, tt, J = 7.5 Hz, J = 1.6 Hz, H-4), 8.04 (2H, dd, J = 8.3 Hz, J = 1.2 Hz, H-2, 6),

9.65 (1H, s, CHO); ^{13}C NMR (acetone-d_6) 65.0 (C-a), 69.8 (C-b), 71.3 (C-c or d or e or f), 71.3 (C-c or d or e or f), 71.4 (C-c or d or e or f), 71.8 (C-c or d or e or f), 77.4 (C-g), 129.4 (2C, C-3, 5), 130.3 (2C, C-2, 6), 131.4 (C-1), 133.9 (C-4), 166.8 (O-C=O), 201.7 (H-C=O); HRMS/ESI m/z $[M+H]^+$ = 297.1282 (calculated for $C_{15}H_{20}O_6$: 297.1333).

2-(2-(2-(((2*s*,3a*R*,9a*S*,*Z*)-3a,4,5,8,9,9a-hexahydrocycloocta[*d*][1,3]dioxol-2-yl)methoxy)ethoxy)ethoxy)ethyl benzoate (14)

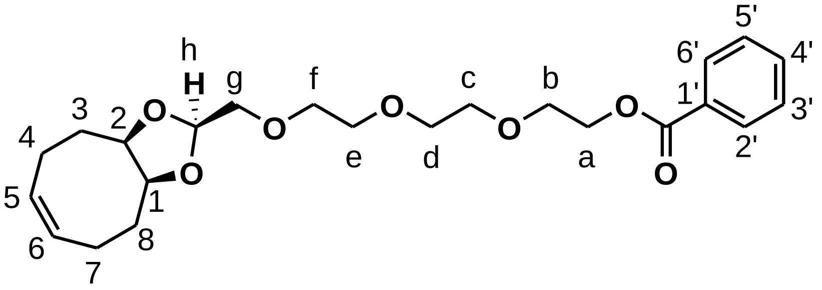

Synthesized according to a modified protocol from Darko *et al.*[3]

To a solution of compound **12** (190 mg; 1.34 mmol) in anhydrous tetrahydrofuran (10 mL) were successively added compound **13** (396 mg; 1.34 mmol; 1.0 eq) and *p*-toluenesulfonic acid monohydrate (127 mg; 0.67 mmol; 0.5 eq.). The reaction mixture was heated at 50 °C for 24 h under N_2 atmosphere. Back to RT, the solution was diluted with ethyl acetate (25 mL) and with a saturated aqueous solution of sodium hydrogencarbonate (25 mL). After separation, the aqueous phase was extracted with ethyl acetate (2 x 25 mL). The organic layers were combined, dried over magnesium sulfate, filtered and evaporated under reduced pressure. The crude product was purified by column chromatography (ethyl acetate/dichloromethane 10:90 → 40:60) to yield **14** (337 mg; 0.80 mmol; 60%) as a colorless oil. Rf on TLC (ethyl acetate/dichloromethane 20:80 v/v) 0.46; ^{1}H NMR ($CDCl_3$) 1.92-1.98 (2H, m, H-3, H-8), 2.01-2.10 (4H, m, H-3, H-8, H-4, H-7), 2.44-2.50 (2H, m, H-4, H-7), 3.56 (2H, d, J = 4.2 Hz, CH_2-g), 3.64-3.70 (8H, m, CH_2-c, d, e, f), 3.82-3.84 (2H, m, CH_2-b), 4.12-4.15 (2H, m, H-1, H-2), 4.46-4.48 (2H, m, CH_2-a), 4.96 (1H, t, J = 4.2 Hz, H-h), 5.58-5.60 (2H, m, H-5, H-6), 7.41-7.45 (2H, m, H-3', 5'), 7.53-7.57 (1H, tt, J = 7.4 Hz, J = 1.6 Hz, H-4'), 8.04-8.06 (2H, m, H-2', 6'); ^{13}C NMR ($CDCl_3$) 23.6 (2C, C-4, C-7), 28.6 (2C, C-3, C-8), 64.3 (C-a), 69.4 (C-b), 70.7 (C-c or d or e or f), 70.8 (C-c or d or e or f), 70.8 (C-c or d or e or f), 71.3 (C-c or d or e or f), 73.2 (C-g), 79.2 (2C, C-1, C-2), 100.8 (C-h), 128.4 (2C, C-3', C-5'), 129.3 (2C, C-5, C-6), 129.8 (2C, C-2', C-6'), 130.2 (C-1'), 133.1 (C-4'), 166.7 (C=O); HRMS/ESI m/z $[M+H]^+$ = 421.2228 (calculated for $C_{23}H_{32}O_7$: 421.2221). The stereochemistry was determined according to Darko *et al.*[3]

2-(2-(2-(((2*s*,3a*R*,9a*S*,*Z*)-3a,4,5,8,9,9a-hexahydrocycloocta[*d*][1,3]dioxol-2-yl)methoxy)ethoxy)ethoxy)ethanol (15)

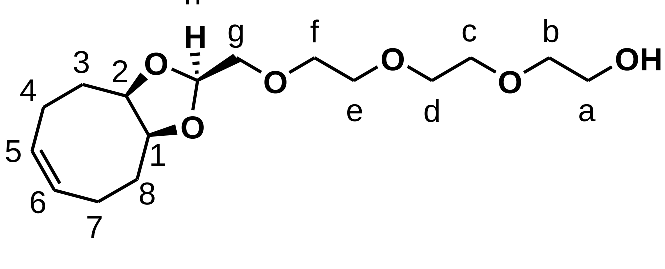

To a solution of compound **14** (322 mg; 0.77 mmol) in absolute ethanol (2.0 mL) was added lithium hydroxide monohydrate (48 mg; 1.15 mmol; 1.5 eq.). The mixture was stirred at RT for 2 h. The mixture was then poured in a mixture of saturated aqueous solution of sodium chloride (20 mL), water (6 mL) and a 5% aqueous solution of sodium carbonate (2 mL). After extraction with dichloromethane (3 x 20 mL), the organic layers were combined, dried over magnesium sulfate, filtered and evaporated under reduced pressure. The crude product was purified by column chromatography (ethyl acetate) to yield **15** (223 mg; 0.71 mmol; 92%) as a colorless oil. Rf on TLC (ethyl acetate) 0.24; ^{1}H NMR ($CDCl_3$) 1.89-1.94 (2H, m, H-3, H-8), 1.96-2.06 (4H, m, H-3, H-8, H-4, H-7), 2.41-2.45 (2H, m, H-4, H-7), 2.70 (1H, br s, OH), 3.53-3.62 (10H, m, CH_2-c, d, e, f, g), 3.64-3.67 (4H, m, CH_2-a, b), 4.07-4.11 (2H, m, H-1, H-2), 4.92 (1H, t, *J* = 4.2 Hz, H-h), 5.54-5.55 (2H, m, H-5, H-6); ^{13}C NMR ($CDCl_3$) 23.5 (2C, C-4, C-7), 28.6 (2C, C-3, C-8), 61.8 (C-a), 70.4 (C-c or d or e or f or g), 70.6 (C-c or d or e or f or g), 70.7 (C-c or d or e or f or g), 71.1 (C-c or d or e or f or g), 72.6 (C-c or d or e or f or g), 73.1 (C-b), 79.2 (2C, C-1, C-2), 100.8 (C-h), 129.3 (2C, C-5, C-6); HRMS/ESI m/z $[M+H]^+$ = 317.1963 (calculated for $C_{16}H_{28}O_6$: 317.1959).

2-(2-(2-(((2*s*,3a*R*,9a*S*,*Z*)-3a,4,5,8,9,9a-hexahydrocycloocta[*d*][1,3]dioxol-2-yl)methoxy)ethoxy)ethoxy)ethyl 4-methylbenzenesulfonate (16)

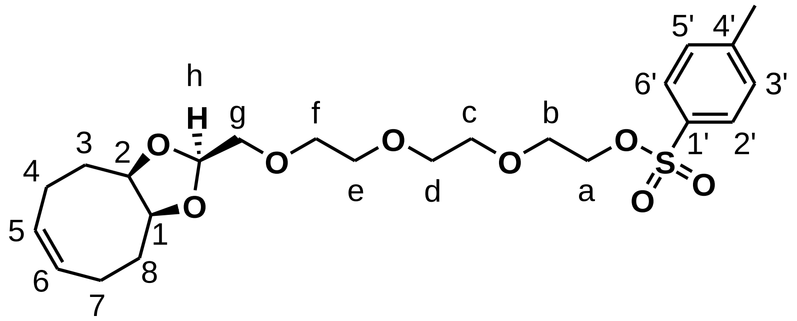

To a solution of compound **15** (400 mg; 1.26 mmol), distilled triethylamine (423 µL; 3.03 mmol; 2.4 eq.), and 4-(dimethylamino)pyridine (31 mg; 0.25 mmol; 0.2 eq.) in dry dichloromethane (15 mL) was added tosyl chloride (289 mg; 1.52 mmol; 1.2 eq.) under N_2 atmosphere. The reaction mixture was stirred at RT for 3 h. The mixture was poured into a saturated aqueous solution of sodium hydrogencarbonate (35 mL). After extraction with dichloromethane (3 x 25 mL), the organic layers were combined, dried over magnesium sulfate, filtered and evaporated under reduced pressure. The crude product was purified by column chromatography (ethyl acetate/*n*-heptane 5:5 → 1:0) to yield **16** (494 mg; 1.05 mmol; 83%) as a colorless oil. Rf on TLC (ethyl acetate/*n*-heptane 5:5) 0.36; ^{1}H NMR ($CDCl_3$) 1.95-1.97 (2H, m, H-3, H-8), 2.05-2.07 (4H, m, H-3, H-8, H-4, H-7), 2.44-2.49 (5H, m, CH_3, H-4, H-7), 3.55-3.69 (12H, m, CH_2-b, c, d, e, f, g), 4.14-4.16 (4H, m, CH_2-a, H-1, H-2), 4.96 (1H, t, *J* = 4.2 Hz, H-h), 5.60 (2H, s, H-5, H-6), 7.34 (2H, d, *J* = 7.8 Hz, H-3', H-5'), 7.79 (2H, d, *J* = 8.1 Hz, H-2', H-6'); ^{13}C NMR ($CDCl_3$) 21.7 (CH_3), 23.5 (2C, C-4, C-7), 28.6 (2C, C-3, C-8), 68.7 (C-b), 69.3 (C-a), 70.6 (2C, C-c or d or e or f or g), 70.8 (C-c or d or e or f or g), 71.2 (C-c or d or e or f or g), 73.2 (C-c or d or e or f or g), 79.2 (2C, C-1, C-2), 100.7 (C-h), 128.0 (2C, C-2', C-6'), 129.3 (2C, C-5, C-6), 129.9 (2C, C-3', C-5'), 133.1 (C-1'), 144.8 (C-4'); HRMS/ESI m/z $[M+NH_4]^+$ = 488.2371 (calculated for $C_{23}H_{34}O_8S$: 488.2313).

(2*s*,3a*R*,9a*S*,*Z*)-2-((2-(2-(2-fluoroethoxy)ethoxy)ethoxy)methyl)-3a,4,5,8,9,9a-hexahydrocycloocta[*d*][1,3]dioxole (18)

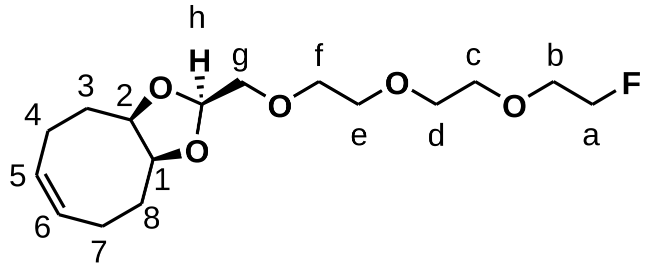

To a solution of compound **16** (175 mg; 0.37 mmol) in dry tetrahydrofuran (1.5 mL) was added a solution of tetrabutylammonium fluoride 1 M in tetrahydrofuran (1.86 mL; 1.86 mmol; 5 eq.) under N_2 atmosphere. The reaction mixture was heated at 80 °C for 2 h. Back to RT, the mixture was poured into a saturated aqueous solution of sodium hydrogencarbonate (20 mL). After extraction with dichloromethane (3 x 20 mL), the organic layers were combined, dried over magnesium sulfate, filtered and evaporated under reduced pressure. The crude product was purified by column chromatography (ethyl acetate/*n*-heptane 5:5) to yield **18** (94 mg; 0.30 mmol; 81%) as a colorless oil. Rf on TLC (ethyl acetate/*n*-heptane 5:5) 0.50; ^{1}H NMR ($CDCl_3$) 1.93-1.98 (2H, m, H-3, H-8), 2.02-2.09 (4H, m, H-3, H-8, H-4, H-7), 2.45-2.50 (2H, m, H-4, H-7), 3.57 (2H, d, J = 4.2 Hz, CH_2-g), 3.64-3.71 (9H, m, H-b, CH_2-c, d, e, f), 3.77 (1H, t, J = 4.2 Hz, H-b), 4.12-4.16 (2H, m, H-1, H-2), 4.55 (2H, dt, J = 4.2 Hz, $^2J_{H\text{-}F}$ = 47.7 Hz, CH_2-a), 4.97 (1H, t, J = 4.2 Hz, H-h), 5.58-5.60 (2H, m, H-5, H-6); ^{13}C NMR ($CDCl_3$) 23.6 (2C, C-4, C-7), 28.6 (2C, C-3, C-8), 70.5 (d, $^2J_{C\text{-}F}$ = 20 Hz, C-b), 70.6 (C-c or d or e or f), 70.7 (C-c or d or e or f), 70.9 (C-c or d or e or f), 71.2 (C-c or d or e or f), 73.2 (C-g), 79.2 (2C, C-1, C-2), 83.2 (d, $^1J_{C\text{-}F}$ = 170 Hz, C-a), 100.8 (C-h), 129.3 (2C, C-5, C-6); HRMS/ESI m/z $[M+NH_4]^+$ = 336.2228 (calculated for $C_{16}H_{27}FO_5$: 336.2181).

2-(2-(2-(((2*s*,3a*R*,9a*S*,*Z*)-3a,4,5,8,9,9a-hexahydrocycloocta[*d*][1,3]dioxol-2-yl)methoxy)ethoxy)ethoxy)ethyl methanesulfonate (17)

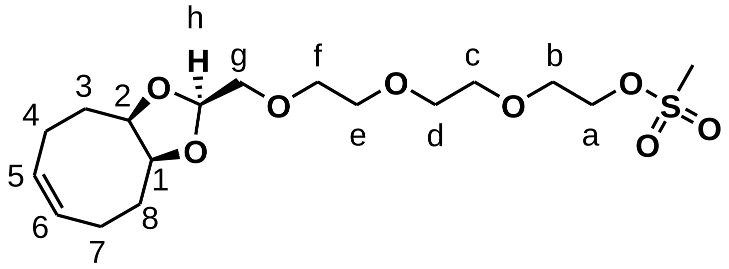

To a solution of compound **15** (150 mg; 0.47 mmol), distilled triethylamine (159 µL; 1.14 mmol; 2.4 eq.), and 4-(dimethylamino)pyridine (12 mg; 95 µmol; 0.2 eq.) in dry dichloromethane (10 mL) was added mesyl chloride (44 µL; 0.57 mmol; 1.2 eq.) under N_2 atmosphere. The reaction mixture was stirred at RT for 90 min. The mixture was poured into a saturated aqueous solution of sodium hydrogencarbonate (25 mL). After extraction with dichloromethane (3 x 20 mL), the organic layers were combined, dried over magnesium sulfate, filtered and evaporated under reduced pressure. The crude product was purified by column chromatography (ethyl acetate/dichloromethane 8:2) to yield **17** (185 mg; 0.47 mmol; 99%) as a pale yellow oil. Rf on TLC (ethyl acetate) 0.50; ^{1}H NMR ($CDCl_3$) 1.93-1.99 (2H, m, H-3, H-8), 2.01-2.12 (4H, m, H-3, H-8, H-4, H-7), 2.45-2.51 (2H, m, H-4, H-7), 3.07 (3H, s, CH_3), 3.56 (2H, d, J = 4.2 Hz, CH_2-g), 3.62-3.70 (8H, m, CH_2-c, d, e, f), 3.75-3.77 (2H, m, CH_2-b), 4.13-4.16 (2H, m, H-1, H-2), 4.36-4.38 (2H, m, CH_2-a), 4.96 (1H, t, J = 4.2 Hz, H-h), 5.59-5.61 (2H, m, H-5, H-6); ^{13}C NMR ($CDCl_3$) 23.5 (2C, C-4, C-7), 28.6 (2C, C-3, C-8), 37.8 (CH_3), 69.0 (C-b), 69.4 (C-a), 70.5 (C-c or d or e or f), 70.6 (C-c or d or e or f), 70.6 (C-c or d or e or f), 71.2 (C-c or d or e or f), 73.1 (C-g), 79.1 (2C, C-1, C-2), 100.7 (C-h), 129.3 (2C, C-5, C-6); HRMS/ESI m/z $[M+NH_4]^+$ = 412.2000 (calculated for $C_{17}H_{30}O_8S$: 412.2000).

2.3 Synthesis of (*S*)-2-(3-((*S*)-1-carboxy-5-(5-oxo-5-((3-(6-(4-(trifluoromethyl)phenyl)-1,2,4,5-tetrazin-3-yl)phenyl)amino)pentanamido)pentyl)ureido)pentanedioic acid (**21**)

(*S*)-di-*tert*-butyl 2-(3-((*S*)-1-(*tert*-butoxy)-1-oxo-6-(5-oxo-5-((3-(6-(4-(trifluoromethyl)phenyl)-1,2,4,5-tetrazin-3-yl)phenyl)amino)pentanamido)hexan-2-yl)ureido)pentanedioate (25)

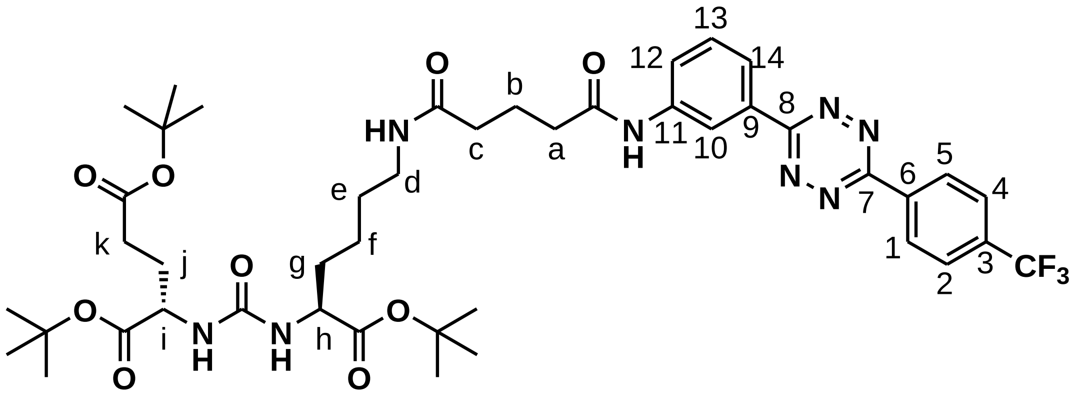

To a solution of 4-(3-(6-(4-(trifluoromethyl)phenyl)-1,2,4,5-tetrazin-3-yl)phenylcarbamoyl)butanoic acid[4] (35 mg; 81 µmol) in dry *N*,*N*-dimethylformamide (800 µL) was added 1-[bis(dimethylamino)methylene]-1*H*-1,2,3-triazolo[4,5-*b*]pyridinium 3-oxid hexafluorophosphate HATU (62 mg; 0.16 mmol; 2 eq.). The reaction mixture was stirred at RT for 15 min and then a solution of 2-[3-(5-amino-1-*tert*-butoxycarbonylpentyl)ureido]pentanedioic acid di-*tert*-butyl ester[5] (44 mg; 89 µmol; 1.1 eq.) and *N*,*N*-diisopropylethylamine (28 µL; 0.16 mmol; 2 eq.) in dry *N*,*N*-dimethylformamide (800 µL) was added. The reaction mixture was then stirred at RT overnight. The mixture was then diluted with dichloromethane (15 mL) and filtered. The filtrate was extracted with water (2 x 150 mL). The organic phase was dried over magnesium sulfate, filtered, and evaporated under reduced pressure. The crude product was purified by column chromatography (dichloromethane/methanol, 98:02) to yield **25** (51 mg; 57 µmol; 70%) as a purple wax. Rf on TLC (dichloromethane/methanol 98:02 v/v) 0.13; ^{1}H NMR (DMSO-d$_6$) 1.24-1.31 (2H, m, CH$_2$-f), 1.38-1.44 (29H, m, C(CH$_3$)$_3$, CH$_2$-e), 1.48-1.71 (3H, m, CH$_2$-g, H-j), 1.83-1.88 (3H, m, CH$_2$-b, H-j), 2.14 (2H, t, *J* = 7.6 Hz, CH$_2$-a or c), 2.18-2.25 (2H, m, CH$_2$-k), 2.38 (2H, t, *J* = 7.6 Hz, CH$_2$-a or c), 3.01-3.04 (2H, m, CH$_2$-d), 3.93-3.99 (1H, m, H-h), 4.01-4.06 (1H, m, H-i), 6.24-6.30 (2H, m, NH), 7.63 (1H, t, *J* = 8.0 Hz, H-13), 7.92 (1H, d, *J* = 8.0 Hz, H-12 or 14), 8.08 (2H, d, *J* = 8.2 Hz, H-2, 4), 8.23 (1H, d, *J* = 8.0 Hz, H-12 or 14), 8.74 (2H, d, *J* = 8.2 Hz, H-1, 5), 8.93 (1H, s, H-10); ^{13}C NMR (DMSO-d$_6$) 21.2 (C-b), 22.5 (C-f), 27.6 (C-j), 27.6 and 27.7 (9C, C(<u>C</u>H$_3$)$_3$), 28.8 (C-e), 30.9 (C-k), 31.7 (C-g), 34.6 (C-a or C-c), 35.8 (C-a or C-c), 38.1 (C-d), 52.1 (C-i), 52.9 (C-h), 79.7 (<u>C</u>(CH$_3$)$_3$), 80.3 (<u>C</u>(CH$_3$)$_3$), 80.6 (<u>C</u>(CH$_3$)$_3$), 118.0 (C-10), 122.3 (C-12 or 14), 123.1 (C-12 or 14), 123.9 (q, $^{1}J_{C\text{-}F}$ = 275 Hz, CF$_3$), 126.3 (q, 2C, $^{3}J_{C\text{-}F}$ = 4 Hz, C-2, C-4), 128.4 (2C, C-1, C-5), 130.0 (C-13), 132.1 (C-9), 132.1 (q, $^{2}J_{C\text{-}F}$ = 31 Hz, C-3), 135.9 (C-6), 140.4 (C-11), 157.0 (NHCONH), 162.6 (C-7 or C-8), 163.5 (C-7 or C-8), 171.3 (CO), 171.4 (CO), 171.8 (CO), 171.9 (CO), 172.2 (CO); ^{19}F NMR (CDCl$_3$) -63.1; HRMS/ESI m/z [M+H]$^+$ = 901.4434 (calculated for $C_{44}H_{59}F_3N_8O_9$: 901.4430).

(*S*)-2-(3-((*S*)-1-carboxy-5-(5-oxo-5-((3-(6-(4-(trifluoromethyl)phenyl)-1,2,4,5-tetrazin-3-yl)phenyl)amino)pentanamido)pentyl)ureido)pentanedioic acid (21)

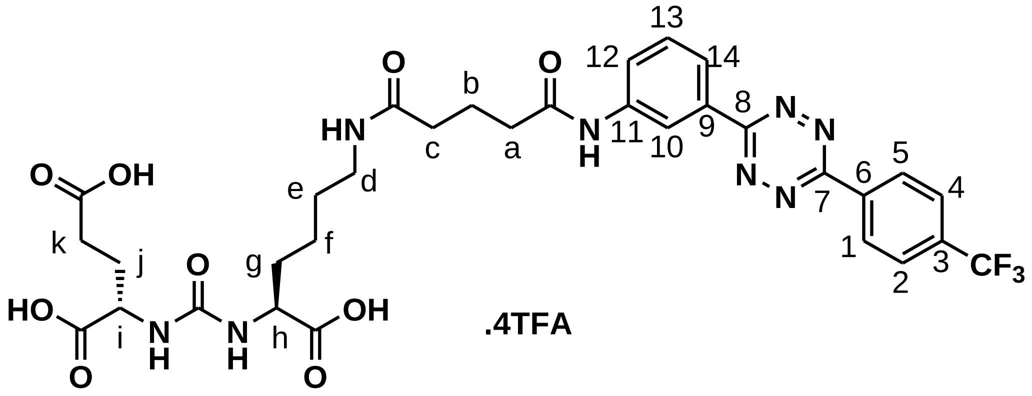

To a solution of compound **25** (22 mg; 24 µmol) in dichloromethane (1.5 mL) was added trifluoroacetic acid (1.0 mL). The reaction mixture was then stirred at RT for 3 h. The solvents were evaporated under reduced pressure. The sample was taken up in absolute ethanol and evaporated under reduced pressure (2 x 5 mL) to give compound **21** (28 mg; 24 µmol; 98%) as a purple wax. ^{1}H NMR (CD_3OD) 1.42-1.47 (2H, m, CH_2-f), 1.51-1.57 (2H, m, CH_2-e), 1.62-1.71 (1H, m, H-g), 1.80-1.92 (2H, m, H-g, H-j), 2.03 (2H, quint., *J* = 7.4 Hz, CH_2-b), 2.08-2.17 (1H, m, H-j), 2.31 (2H, t, *J* = 7.4 Hz, CH_2-a or c), 2.38-2.42 (2H, m, CH_2-k), 2.48 (2H, t, *J* = 7.4 Hz, CH_2-a or c), 3.18-3.22 (2H, m, CH_2-d), 4.26-4.33 (2H, m, H-h, H-i), 7.60 (1H, t, *J* = 8.0 Hz, H-13), 7.91 (1H, d, *J* = 8.0 Hz, H-12 or 14), 7.97 (2H, d, *J* = 8.5 Hz, H-2, 4), 8.37 (1H, d, *J* = 8.0 Hz, H-12 or 14), 8.81 (2H, d, *J* = 8.2 Hz, H-1, 5), 8.89 (1H, s, H-10); ^{13}C NMR (CD_3OD) 23.0 (C-b), 23.9 (C-f), 28.9 (C-j), 29.9 (C-e), 31.1 (C-k), 33.2 (C-g), 36.2 (C-a or C-c), 37.1 (C-a or C-c), 40.1 (C-d), 53.5 (C-h or C-i), 53.9 (C-h or C-i), 120.5 (C-10), 124.6 (C-12 or 14), 125.3 (C-12 or 14), 125.4 (q, $^{1}J_{C-F}$ = 273 Hz, CF_3), 127.2 (q, 2C, $^{3}J_{C-F}$ = 4 Hz, C-2, C-4), 129.5 (2C, C-1, C-5), 130.9 (C-13), 133.9 (C-9), 134.9 (q, $^{2}J_{C-F}$ = 33 Hz, C-3), 137.3 (C-6), 141.1 (C-11), 160.1 (NHCONH), 164.6 (C-7 or C-8), 165.5 (C-7 or C-8), 174.1 (CO), 175.4 (CO), 175.9 (CO), 176.4 (2C, CO); ^{19}F NMR (CD_3OD) -64.0 (Ar-CF_3), -76.9 (CF_3CO); HRMS/ESI m/z $[M+H]^+$ = 733.2558 (calculated for $C_{32}H_{35}F_3N_8O_9$: 733.2552).

3. Photoisomerizations

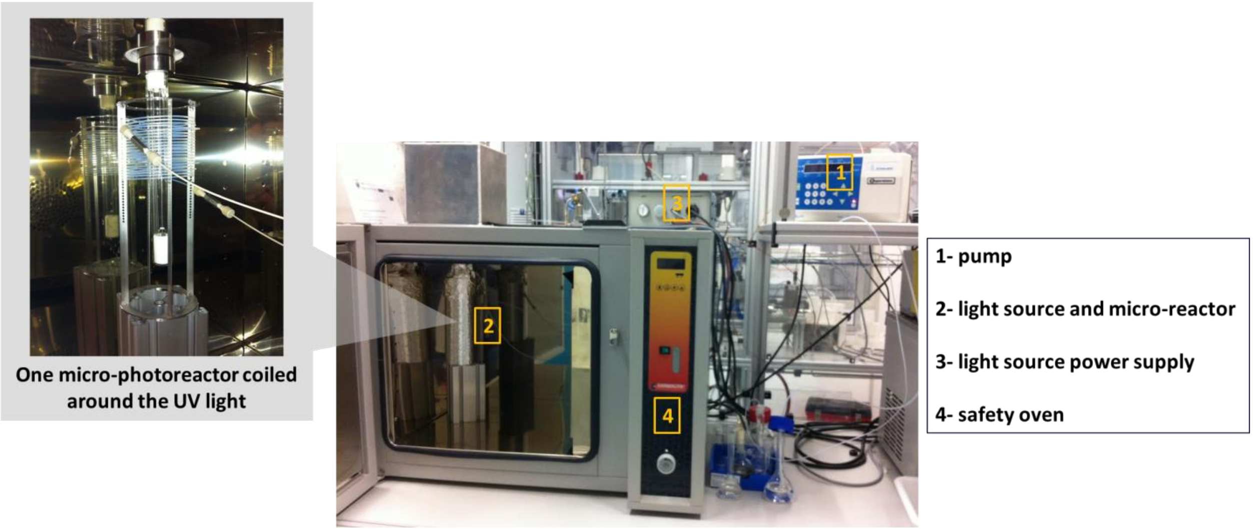

Figure S1. The microfluidic device used for *trans*-for-*cis* isomerization of functionalised cyclooctene derivatives

(*E*)-2-(2-(2-(2-(cyclooct-4-en-1-yloxy)ethoxy)ethoxy)ethoxy)ethanol (10a and 10b)

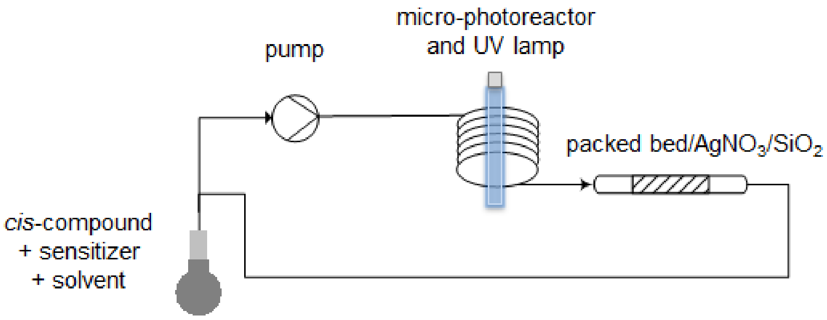

To a solution of compound **6** (364 mg; 1.20 mmol) in a mixture diethyl ether/*n*-hexane (4:1, 50 mL) was added methyl benzoate (377 mg; 2.77 mmol; 2.3 eq.). The reaction mixture was pumped through the microreactor (L 2.5 m) and then to a column (L 52 mm) filled with silver nitrate-impregnated silica gel (314 mg) and glass beads (200 mg of 1 mm beads and 70 mg of 212-300 µm beads), at an average flow rate of 0.2 $mL.min^{-1}$, to achieve a residence time of 2.5 min in the photoreactor. After 10 h of experiment, the packed bed was washed with diethyl ether/*n*-hexane (4:1), and then the column was emptied in an Erlenmeyer flask. The silica gel was stirred with a mixture of ammonium hydroxide solution (28% NH_3 basis, 10 mL) and dichloromethane (10 mL) for 5 min. After filtration, the filtrate was transferred to an extraction funnel. The organic phase was separated, and the aqueous phase was extracted with dichloromethane (3 x 15 mL). The organic layers were combined, dried over magnesium sulfate, filtered and evaporated under reduced pressure. The crude products were purified by column chromatography (ethyl acetate/ethanol 100:0 then 97:3) to yield **10a** (major isomer; 33 mg; 9%) and **10b** (minor isomer; 20 mg; 6%) as colorless oils. The stereochemistry was determined according to Royzen *et al.*[6]

MAJOR compound **10a**:

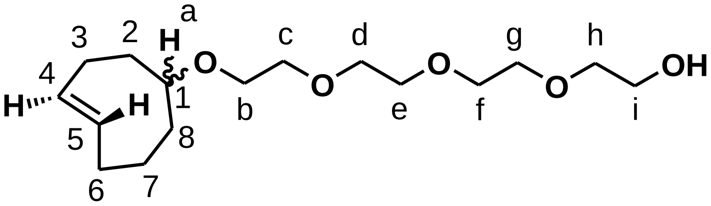

Rf on TLC (ethyl acetate) 0.15; ^{1}H NMR ($CDCl_3$) 1.46-1.49 (2H, m, H-7, H-8), 1.79-1.85 (2H, m, H-2, H-8), 1.88-1.96 (2H, m, H-6, H-7), 2.05-2.09 (1H, m, H-2), 2.18-2.22 (1H, m, H-3), 2.32-2.34 (2H, m, H-3, H-6), 2.53 (1H, br s, OH), 2.98-3.00 (1H, m, H-a), 3.41-3.44 (1H, m, H-b), 3.50-3.64 (13H, m, H-b, CH_2-c, d, e, f, g, h), 3.69-3.70 (2H, m, CH_2-i), 5.32-5.38 (1H, m, H-5), 5.52-5.58 (1H, m, H-4); ^{13}C NMR ($CDCl_3$) 31.9 (C-7), 33.1 (C-3), 34.6 (C-6), 37.8 (C-8), 40.8 (C-2), 61.8 (C-i), 67.5 (C-b), 70.3 (C-c, d, e, f or g), 70.6 (2C, C-c, d, e, f or g), 70.7 (C-c, d, e, f or g), 70.9 (C-c, d, e, f or g), 72.8 (C-h), 86.2 (C-1), 132.3 (C-5), 135.5 (C-4); HRMS/ESI m/z $[M+H]^+$ = 303.2200 (calculated for $C_{16}H_{30}O_5$: 303.2166).

MINOR compound **10b**:

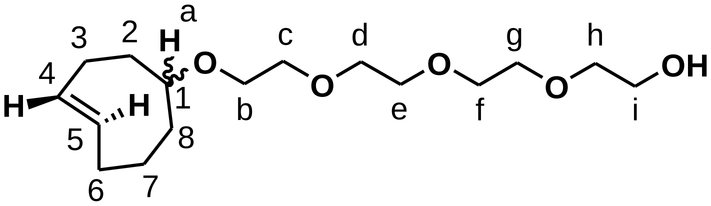

Rf on TLC (ethyl acetate) 0.30; ^{1}H NMR ($CDCl_3$) 1.10-1.16 (1H, m, H-8), 1.42-1.48 (1H, m, H-2), 1.70-1.72 (1H, m, H-7), 1.76-1.84 (2H, m, H-6, H-7), 1.98-2.00 (1H, m, H-3), 2.11-2.22 (2H, m, H-6, H-8), 2.28-2.32 (2H, m, H-2, H-3), 2.84 (1H, br s, OH), 3.44-3.49 (1H, m, H-b), 3.56-3.71 (16H, m, H-a, H-b, CH_2-c, d, e, f, g, h, i), 5.43-5.49 (1H, m, H-4), 5.57-5.63 (1H, m, H-5); ^{13}C NMR ($CDCl_3$) 27.7 (C-7), 29.9 (C-3), 33.1 (C-8), 34.7 (C-6), 40.4 (C-2), 61.9 (C-i), 68.4 (C-b), 70.5 (C-c, d, e, f or g), 70.8 (C-c, d, e, f or g), 70.8 (C-c, d, e, f or g), 70.9 (C-c, d, e, f or g), 71.0 (C-c, d, e, f or g), 72.7 (C-h), 75.0 (C-1), 131.5 (C-4), 136.0 (C-5); HRMS/ESI m/z $[M+H]^+$ = 303.2187 (calculated for $C_{16}H_{30}O_5$: 303.2166).

2-(2-(2-(((2*s*,3a*R*,9a*S*,*E*)-3a,4,5,8,9,9a-hexahydrocycloocta[*d*][1,3]dioxol-2-yl)methoxy)ethoxy)ethoxy)ethanol (19)

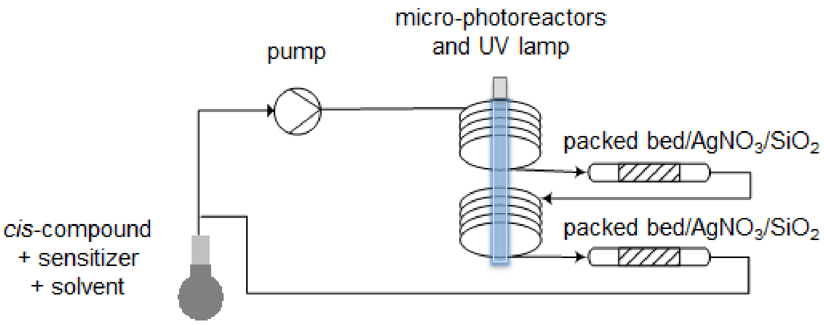

To a solution of compound **15** (85 mg; 0.27 mmol) in *tert*-butyl methyl ether (25 mL) was added methyl benzoate (201 mg; 1.48 mmol; 5.5 eq.). The reaction mixture was pumped through a first microreactor (L 2.5 m) followed by a first column (L 52 mm) filled with silver nitrate-impregnated silica gel (324 mg) and glass beads (35 mg of 212-300 µm beads and 110 mg of 1 mm beads), then through a second microreactor (L 1.0 m) followed by a second column (L 25 mm) filled with silver nitrate-impregnated silica gel (160 mg) and glass beads (37 mg of 212-300 µm beads and 120 mg of 1 mm beads), at a flow rate

of 0.1 mL.min^{-1}, to achieve a residence time of 2.5 min in the first reactor and 1.0 min in the second reactor. After 10 h of experiment, the packed beds were washed with *tert*-butyl methyl ether, and then the columns were emptied in an Erlenmeyer flask. The silica gel was stirred with a mixture of ammonium hydroxide solution (28% NH_3 basis, 5 mL) and dichloromethane (5 mL) for 5 min. After filtration, the filtrate was transferred to an extraction funnel. The organic phase was separated, and the aqueous phase was extracted with dichloromethane (3 x 10 mL). The organic layers were combined, dried over magnesium sulfate, filtered and evaporated under reduced pressure to yield **19** (18 mg; 57 µmol; 21%) as a colorless oil. ^{1}H NMR ($CDCl_3$) 1.48-1.57 (1H, m, H-3 or 8), 1.64-1.72 (1H, m, H-3 or 8), 1.79-1.91 (2H, m, H-3 or 8, and H-4 or 7), 2.07-2.28 (3H, m, H-4 or 7, H-3 or 8, and H-4 or 7), 2.35-2.42 (1H, m, H-4 or 7), 3.55 (2H, t, J = 4.1 Hz, CH_2-g), 3.58-3.72 (12H, m, CH_2-a, b, c, d, e, f), 3.87-3.96 (2H, m, H-1, H-2), 4.89 (1H, t, J = 4.1 Hz, H-h), 5.46-5.71 (2H, m, H-5, H-6); ^{13}C NMR ($CDCl_3$) 25.7 (C-4 or 7), 31.4 (C-4 or 7), 33.9 (C-3 or 8), 38.8 (C-3 or 8), 61.9 (C-a), 70.4 (C-c or d or e or f), 70.7 (C-c or d or e or f), 70.8 (C-c or d or e or f), 71.1 (C-c or d or e or f), 72.7 (C-b or C-g), 72.8 (C-b or C-g), 80.7 (C-1 or 2), 82.7 (C-1 or 2), 100.3 (C-h), 131.3 (C-5 or 6), 136.5 (C-5 or 6); HRMS/ESI m/z $[M+H]^+$ = 317.1984 (calculated for $C_{16}H_{28}O_6$: 317.1959). The stereochemistry was determined according to Darko *et al.*[3]

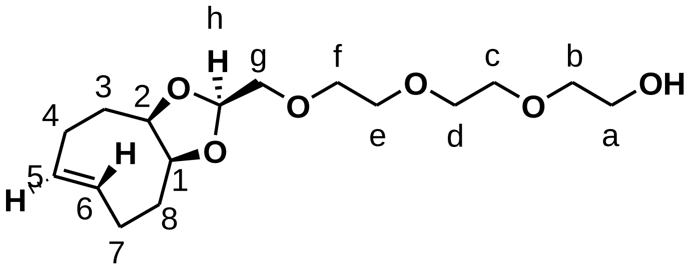

(*E*)-5-(2-(2-(2-(2-fluoroethoxy)ethoxy)ethoxy)ethoxy)cyclooct-1-ene (2a and 2b)

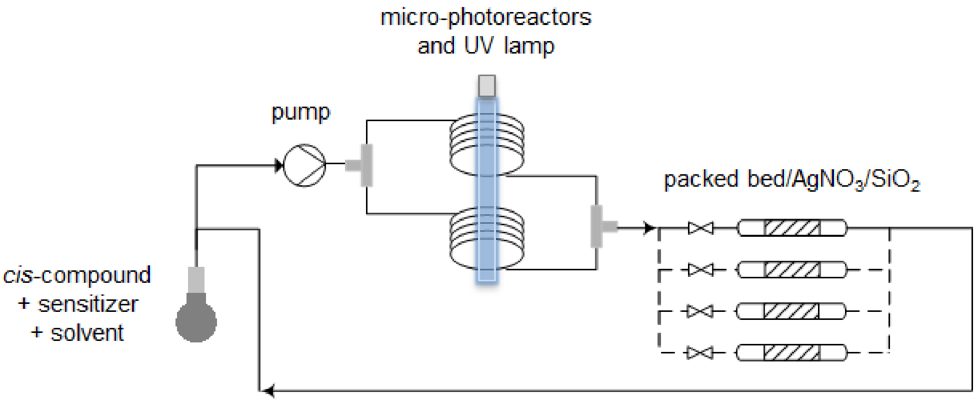

To a solution of compound **9** (82 mg; 0.27 mmol) in a mixture diethyl ether/*n*-hexane (1:1, 25 mL) was added methyl benzoate (169 mg; 1.24 mmol; 4.6 eq.). The reaction mixture was pumped through two microreactors (L 5.0 m) in parallel followed by four columns (L 25 mm) in parallel (switch from one column to another every 90 min) filled with silver nitrate-impregnated silica gel (200-220 mg in each column) and glass beads (100-120 mg of 1 mm beads in each column), at a flow rate of 1.0 mL.min^{-1}, to achieve a residence time of 2.0 min in each photoreactor. After 6 h of experiment, the packed beds were washed with diethyl ether/*n*-hexane (1:1), and then the column was emptied in an Erlenmeyer flask. The silica gel was stirred with a mixture of ammonium hydroxide solution (28% NH_3 basis, 5 mL) and dichloromethane (5 mL) for 5 min. After filtration, the filtrate was transferred to an extraction funnel. The organic phase was separated, and the aqueous phase was extracted with dichloromethane (3 x 10 mL). The organic layers were combined, washed with water, dried over magnesium sulfate, filtered and evaporated under reduced pressure. The crude products were purified by column chromatography (ethyl

acetate/*n*-heptane 2:8 then 5:5) to yield **2a** (major isomer; 16 mg; 20%) and **2b** (minor isomer; 4 mg; 5%) as colorless oils. The stereochemistry was determined according to Royzen *et al.*[6]

Major compound **2a**:

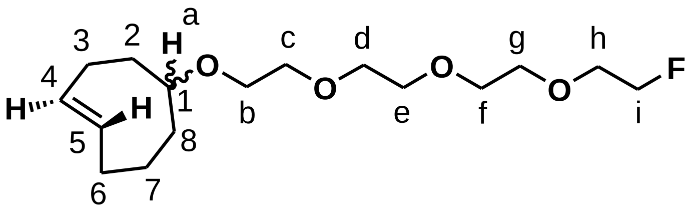

Rf on TLC (ethyl acetate/*n*-heptane 2:8) 0.11; ^{1}H NMR ($CDCl_3$) 1.47-1.50 (2H, m, H-7, H-8), 1.76-1.82 (2H, m, H-2, H-8), 1.90-1.97 (2H, m, H-6, H-7), 2.07-2.11 (1H, m, H-2), 2.20-2.24 (1H, m, H-3), 2.34-2.36 (2H, m, H-3, H-6), 3.00-3.03 (1H, m, H-a), 3.42-3.45 (1H, m, H-b), 3.51-3.68 (12H, m, H-b, H-h, CH_2-c, d, e, f, g), 3.77-3.79 (1H, m, H-h), 4.56 (2H, d, $^{2}J_{H\text{-}F}$ = 47.7 Hz, CH_2-i), 5.33-5.40 (1H, m, H-5), 5.54-5.60 (1H, m, H-4); ^{13}C NMR ($CDCl_3$) 31.9 (C-7), 33.2 (C-3), 34.7 (C-6), 37.9 (C-8), 41.0 (C-2), 67.6 (C-b), 70.5 (d, $^{2}J_{C\text{-}F}$ = 20 Hz, C-h), 70.8 (3C, C-c, d, e, f or g), 71.0 (2C, C-c, d, e, f or g), 83.3 (d, $^{1}J_{C\text{-}F}$ = 170 Hz, C-i), 86.2 (C-1), 132.4 (C-5), 135.5 (C-4); HRMS/ESI m/z $[M+H]^+$ = 305.2143 (calculated for $C_{16}H_{29}FO_4$: 305.2123).

Minor compound **2b**:

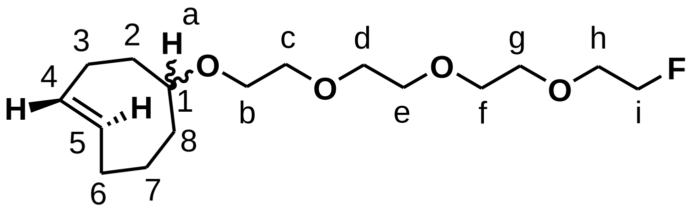

Rf on TLC (ethyl acetate/*n*-heptane 2:8) 0.21; ^{1}H NMR ($CDCl_3$) 1.11-1.17 (1H, m, H-8), 1.43-1.50 (1H, m, H-2), 1.69-1.71 (1H, m, H-7), 1.78-1.86 (2H, m, H-6, H-7), 1.98-2.01 (1H, m, H-3), 2.14-2.31 (4H, m, H-6, H-8, H-2, H-3), 3.45-3.49 (1H, m, H-b), 3.56-3.59 (2H, m, H-a, H-b), 3.66-3.69 (11H, m, H-h, CH_2-c, d, e, f, g), 3.78-3.80 (1H, m, H-h), 4.56 (2H, d, $^{2}J_{H\text{-}F}$ = 47.2 Hz, CH_2-i), 5.44-5.50 (1H, m, H-4), 5.59-5.65 (1H, m, H-5); ^{13}C NMR ($CDCl_3$) 27.7 (C-7), 29.9 (C-3), 33.2 (C-8), 34.7 (C-6), 40.4 (C-2), 68.4 (C-b), 70.6 (d, $^{2}J_{C\text{-}F}$ = 20 Hz, C-h), 70.8 (C-c, d, e, f or g), 71.0 (C-c, d, e, f or g), 71.0 (C-c, d, e, f or g), 71.0 (2C, C-c, d, e, f or g), 75.0 (C-1), 83.3 (d, $^{1}J_{C\text{-}F}$ = 168 Hz, C-i), 131.4 (C-4), 136.0 (C-5); HRMS/ESI m/z $[M+H]^+$ = 305.2149 (calculated for $C_{16}H_{29}FO_4$: 305.2123).

(2*s*,3a*R*,9a*S*,*E*)-2-((2-(2-(2-fluoroethoxy)ethoxy)ethoxy)methyl)-3a,4,5,8,9,9a-hexahydrocycloocta[*d*][1,3]dioxole (3)

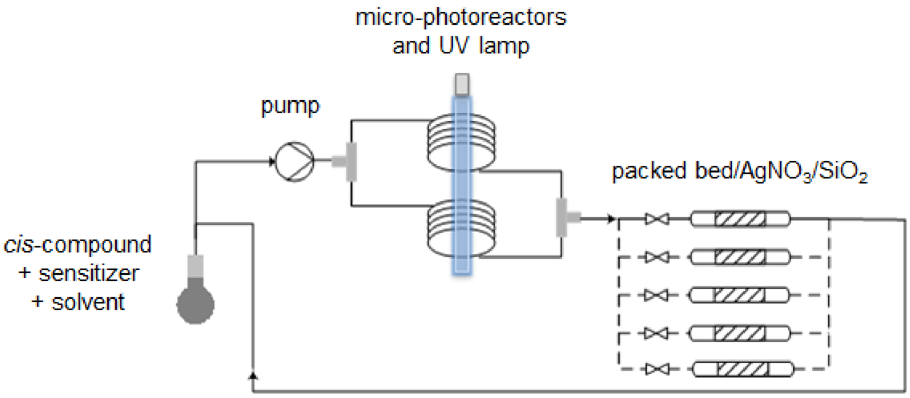

To a solution of compound **18** (37 mg; 0.12 mmol) in *tert*-butyl methyl ether (10 mL) was added methyl benzoate (70 mg; 0.51 mmol; 4.3 eq.). The reaction mixture was pumped through two microreactors (L 5.0 m) in parallel followed by five columns (L 25 mm) in parallel (switch from one column to another every 75 min) filled with silver nitrate-impregnated silica gel (200-220 mg in each column) and glass beads (100-120 mg of 1 mm beads in each column), at a flow rate of 0.66-1.0 $mL.min^{-1}$, to achieve an average residence time of 3.0 min in each photoreactor. After 6 h of experiment, the packed beds were washed with *tert*-butyl methyl ether, and then the columns were emptied in an Erlenmeyer flask. The silica gel was stirred with a mixture of ammonium hydroxide solution (28% NH_3 basis, 5 mL) and dichloromethane (5 mL) for 5 min. After filtration, the filtrate was transferred to an extraction funnel. The organic phase was separated, and the aqueous phase was extracted with dichloromethane (3 x 10 mL). The organic layers were combined, washed with water, dried over magnesium sulfate, filtered and evaporated under reduced pressure to yield **3** (29 mg; 91 µmol; 76%) as a colorless oil. ^{1}H NMR ($CDCl_3$) 1.48-1.58 (1H, m, H-3 or 8), 1.62-1.73 (1H, m, H-3 or 8), 1.80-1.92 (2H, m, H-3 or 8, and H-4 or 7), 2.08-2.28 (3H, m, H-4 or 7, H-3 or 8, and H-4 or 7), 2.36-2.44 (1H, m, H-4 or 7), 3.55 (2H, dd, J = 4.2 Hz, J = 3.0 Hz, CH_2-g), 3.64-3.71 (9H, m, H-b, CH_2-c, d, e, f), 3.77-3.79 (1H, m, H-b), 3.87-3.96 (2H, m, H-1, H-2), 4.56 (2H, dt, J = 4.1 Hz, $^{2}J_{H\text{-}F}$ = 47.7 Hz, CH_2-a), 4.89 (1H, t, J = 4.2 Hz, H-h), 5.47-5.66 (2H, m, H-5, H-6); ^{13}C NMR ($CDCl_3$) 25.8 (C-4 or 7), 31.4 (C-4 or 7), 33.9 (C-3 or 8), 38.9 (C-3 or 8), 70.5 (d, $^{2}J_{C\text{-}F}$ = 20 Hz, C-b), 70.7 (C-c or d or e or f), 70.8 (C-c or d or e or f), 71.0 (C-c or d or e or f), 71.3 (C-c or d or e or f), 72.8 (C-g), 80.7 (C-1 or 2), 82.7 (C-1 or 2), 83.3 (d, $^{1}J_{C\text{-}F}$ = 168 Hz, C-a), 100.4 (C-h), 131.4 (C-5 or 6), 136.5 (C-5 or 6); ^{19}F NMR ($CDCl_3$) -222.9; HRMS/ESI m/z $[M+NH_4]^+$ = 336.2217 (calculated for $C_{16}H_{27}FO_5$: 336.2181). The stereochemistry was determined according to Darko *et al.*[3]

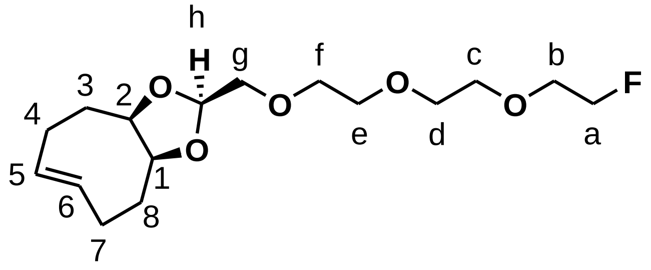

2-(2-(2-(((2*s*,3a*R*,9a*S*,*E*)-3a,4,5,8,9,9a-hexahydrocycloocta[*d*][1,3]dioxol-2-yl)methoxy)ethoxy)ethoxy)ethyl methanesulfonate (20)

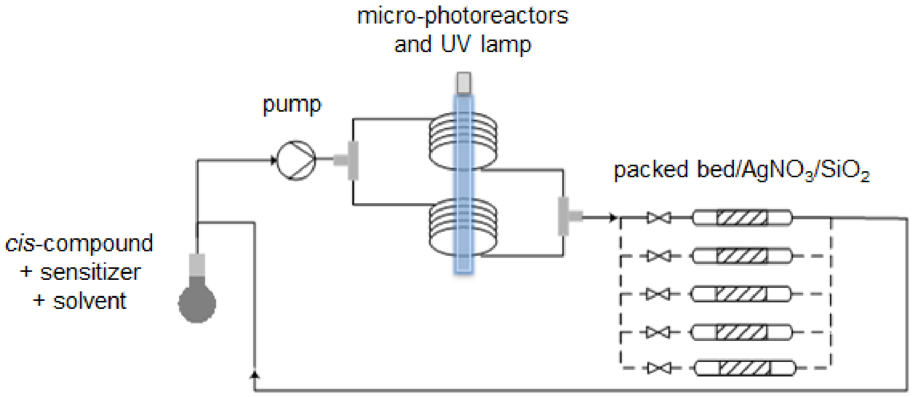

To a solution of compound **17** (61 mg; 0.16 mmol) in a mixture diethyl ether/*n*-hexane (3:7, 10 mL) was added methyl benzoate (114 mg; 0.84 mmol; 5.3 eq.). The reaction mixture was pumped through two microreactors (L 5.0 m) in parallel followed by five columns (L 25 mm) in parallel (switch from one column to another every 75 min) filled with silver nitrate-impregnated silica gel (200-220 mg in each column) and glass beads (100-120 mg of 1 mm beads in each column), at a flow rate of 1.0-1.2 $mL.min^{-1}$, to achieve an average residence time of 2.5 min in each photoreactor. After 6 h of experiment, the

packed beds were washed with diethyl ether/*n*-hexane (3:7), and then the columns were emptied in an Erlenmeyer flask. The silica gel was stirred with a mixture of ammonium hydroxide solution (28% NH_3 basis, 5 mL) and dichloromethane (5 mL) for 5 min. After filtration, the filtrate was transferred to an extraction funnel. The organic phase was separated, and the aqueous phase was extracted with dichloromethane (2 x 10 mL). The organic layers were combined, washed with water, dried over magnesium sulfate, filtered and evaporated under reduced pressure and then under N_2 flow. The crude product was purified by semi-preparative HPLC (Column Waters XBridge C18, 5 µm, 4.6 mm x 150 mm; flow rate: 1.0 mL.min^{-1}; UV detection: 210 nm; mobile phase: ammonium acetate buffer, 50 µM, pH 8/EtOH 74:26 v/v). The solvents were removed by lyophilisation to yield **20** as a pale yellow oil (27 mg; 69 µmol; 44%). ^{1}H NMR ($CDCl_3$) 1.54-1.59 (1H, m, H-3 or 8), 1.63-1.73 (1H, m, H-3 or 8), 1.80-1.93 (2H, m, H-3 or 8, and H-4 or 7), 2.05-2.30 (3H, m, H-4 or 7, H-3 or 8, H-4 or 7), 2.36-2.44 (1H, m, H-4 or 7), 3.08 (3H, s, CH_3), 3.54 (2H, dd, J = 4.2 Hz, J = 2.1 Hz, CH_2-g), 3.63-3.67 (8H, m, CH_2-c, d, e, f), 3.75-3.77 (2H, m, CH_2-b), 3.87-3.97 (2H, m, H-1, H-2), 4.37-4.39 (2H, m, CH_2-a), 4.89 (1H, t, J = 4.2 Hz, H-h), 5.47-5.66 (2H, m, H-5, H-6). ^{13}C NMR ($CDCl_3$) 25.7 (C-4 or 7), 31.4 (C-4 or 7), 33.9 (C-3 or 8), 37.8 (CH_3), 38.7 (C-3 or 8), 69.1 (C-b), 69.4 (C-a), 70.6-71.2 (4C, C-c, d, e, f), 72.8 (C-g), 80.7 (C-1 or 2), 82.6 (C-1 or 2), 100.3 (C-h), 131.3 (C-5 or 6), 136.4 (C-5 or 6); HRMS/ESI m/z $[M+NH_4]^+$ = 412.2002 (calculated for $C_{17}H_{30}O_8S$: 412.2000). The stereochemistry was determined according to Darko *et al.*[3]

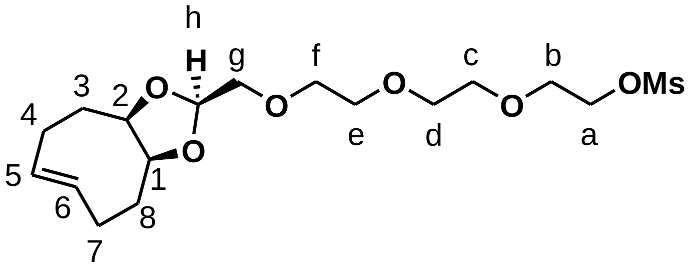

***Trans*-cyclooctenol**

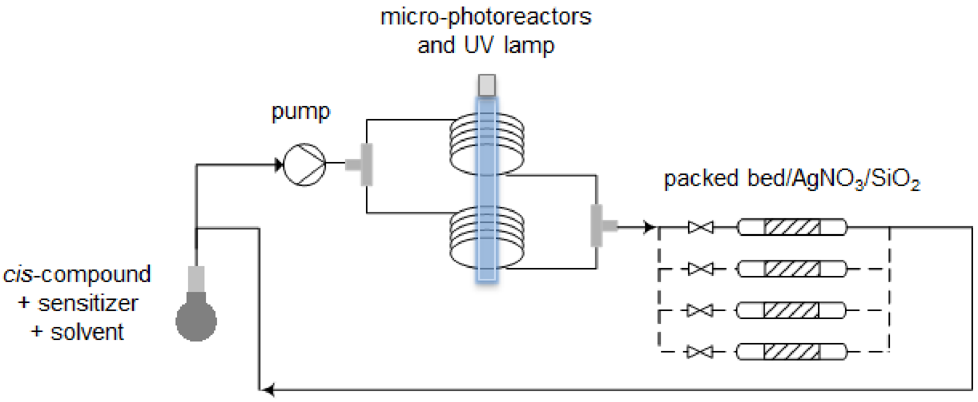

To a solution of *cis*-cyclooctenol (40 mg; 0.32 mmol) in a mixture diethyl ether/*n*-hexane (1:4, 15 mL) was added methyl benzoate (95 mg; 0.70 mmol; 2.2 eq.). The reaction mixture was pumped through two microreactors (L 5.0 m) in parallel followed by four columns (L 25 mm) in parallel (switch from one column to another every 30-45 min) filled with silver nitrate-impregnated silica gel (200-220 mg in each column) and glass beads (100-120 mg of 1 mm beads in each column), at a flow rate of 0.66-0.80 mL.min^{-1}, to achieve an average residence time of 3.0 min in each photoreactor. After 3.25 h of experiment, the packed beds were washed with diethyl ether/*n*-hexane (1:4), and then the column was emptied in an Erlenmeyer flask. The silica gel was stirred with a mixture of ammonium hydroxide solution (28% NH_3 basis, 5 mL) and dichloromethane (5 mL) for 5 min. After filtration, the filtrate was transferred to an extraction funnel. The organic phase was separated, and the aqueous phase was extracted with dichloromethane (3 x 10 mL). The organic layers were combined, washed with water, dried over

magnesium sulfate, filtered and evaporated under reduced pressure. The crude products were purified by column chromatography (ethyl acetate/*n*-heptane 1:4) to yield *trans*-cyclooctenol major isomer (28 mg; 70%) and *trans*-cyclooctenol minor isomer (6 mg; 15%) as colorless oils. The stereochemistry was determined according to Royzen *et al.*[6]

Major compound:

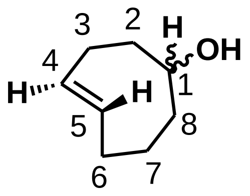

Rf on TLC (ethyl acetate/*n*-heptane 1:4 v/v) 0.19; ^{1}H NMR ($CDCl_3$) 1.25 (1H, s, OH), 1.54-1.71 (3H, m, H-7, H-8, H-8), 1.89-1.98 (4H, m, H-2, H-2, H-6, H-7), 2.23-2.36 (3H, m, H-3, H-3, H-6), 3.43-3.48 (1H, m, H-1), 5.34-5.42 (1H, m, H-5), 5.53-5.61 (1H, m, H-4); ^{13}C NMR ($CDCl_3$) 31.4 (C-7), 32.8 (C-3), 34.5 (C-6), 41.2 (C-8), 44.7 (C-2), 77.9 (C-1), 132.9 (C-5), 135.2 (C-4).

Minor compound:

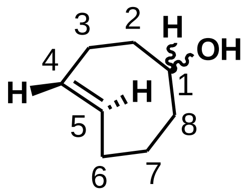

Rf on TLC (ethyl acetate/*n*-heptane 1:4 v/v) 0.37; ^{1}H NMR ($CDCl_3$) 1.21-1.30 (2H, m, OH, H-8), 1.61-1.70 (1H, m, H-2), 1.75-1.87 (3H, m, H-6, H-7, H-7), 2.06-2.17 (2H, m, H-3, H-8), 2.19-2.27 (2H, m, H-2, H-6), 2.32-2.42 (1H, m, H-3), 4.02-4.06 (1H, m, H-1), 5.55-5.58 (2H, m, H-4, H-5); ^{13}C NMR ($CDCl_3$) 27.8 (C-7), 29.4 (C-3), 34.2 (C-6 or C-8), 34.2 (C-6 or C-8), 43.1 (C-2), 67.5 (C-1), 133.2 (C-4 or C-5), 134.4 (C-4 or C-5).

4. Kinetics

The reaction between the dienophile (**10a**, **10b** or **19**) and 3,6-di(pyridin-2-yl)-1,2,4,5-tetrazine (diPy-Tz) was monitored by UV-visible spectrophotometry at 290 nm under pseudo-first order conditions. The dienophile (1.5 mL, 200 µM in methanol) was added to diPy-Tz (1.5 mL, 20 µM in methanol) in a 3.0 mL cuvette. The final concentrations were 100 µM for the dienophile and 10 µM for diPy-Tz. Analyses were carried out in triplicate at RT. For each run, the absorbance at 290 nm was acquired every 5 s, during 100-600 s. The k_{obs} was determined by nonlinear regression analysis of the data points using GraphPad Prism 5 software. Results are displayed in Figure S2 and Table S1.

The reaction between *trans*-cyclooctenol major, *trans*-cyclooctenol minor or ((1*R*,8*S*,9*s*)-bicyclo[6.1.0]non-4-yn-9-yl)methyl 4-hydroxybutylcarbamate (BCN-OH) and diPy-Tz was also monitored by UV-visible spectrophotometry at 290 nm under pseudo-first order conditions, following the same experimental protocol, for comparison. Results are displayed in Figure S2 and Table S1.

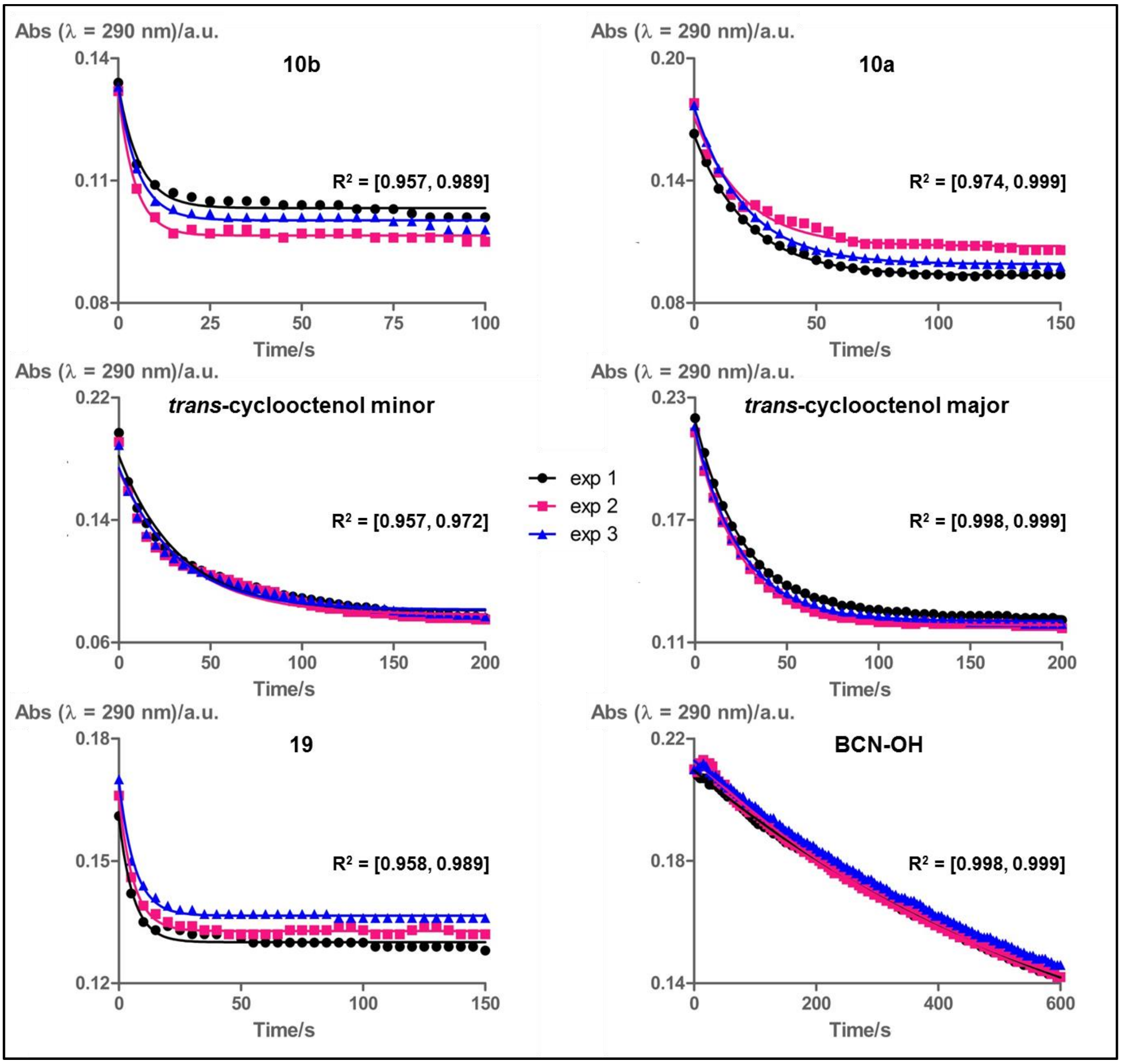

Figure S2. Reaction between dienophiles (**10b**, **10a**, **19**, *trans*-cyclooctenol minor, *trans*-cyclooctenol major or BCN-OH) and 3,6-di(pyridin-2-yl)-1,2,4,5-tetrazine in MeOH at RT was monitored by UV-visible spectrophotometry at 290 nm. Graphs represent the absorbance at 290 nm (a.u.) as a function of time (seconds).

	k_{obs} (s^{-1})	k_2 ($M^{-1}.s^{-1}$)	half-life (s)
**10a**	0.04756 ± 0.00328	476 ± 33	14.6
**10b**	0.19130 ± 0.01959	1913 ± 196	3.7
**19**	0.16197 ± 0.01493	1620 ± 149	4.3
***trans*-cyclooctenol major**	0.03918 ± 0.00064	392 ± 6	17.7
***trans*-cyclooctenol minor**	0.03001 ± 0.00223	300 ± 22	23.1
**BCN-OH**	0.00153 ± 0.00005	15 ± 1	461.9

Table S1. Rate constants and corresponding half-lives for the reaction between dienophiles (**10b**, **10a**, **19**, *trans*-cyclooctenol minor, *trans*-cyclooctenol major or BCN-OH) and 3,6-di(pyridin-2-yl)-1,2,4,5-tetrazine in MeOH at RT measured under pseudo-first order conditions using a UV-visible spectrophotometer

For each experiment, LC-HRMS of the reaction mixture was performed, to confirm the presence of the IEDDA reaction products:

- Reaction of **10a** with 3,6-di(pyridin-2-yl)-1,2,4,5-tetrazine:

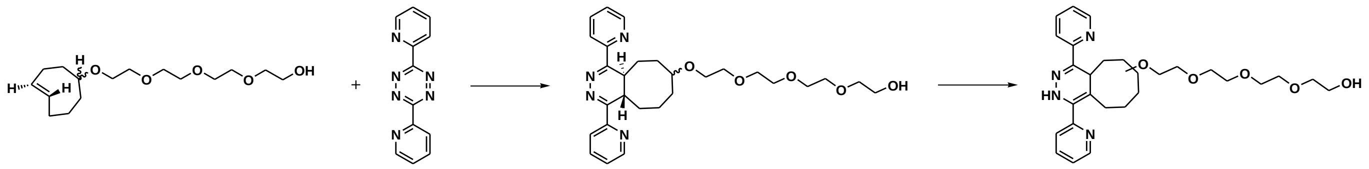

HRMS/ESI m/z $[M+H]^+$ = 511.2935 (calculated for $C_{28}H_{38}N_4O_5$: 511.2915)

- Reaction of **10b** with 3,6-di(pyridin-2-yl)-1,2,4,5-tetrazine:

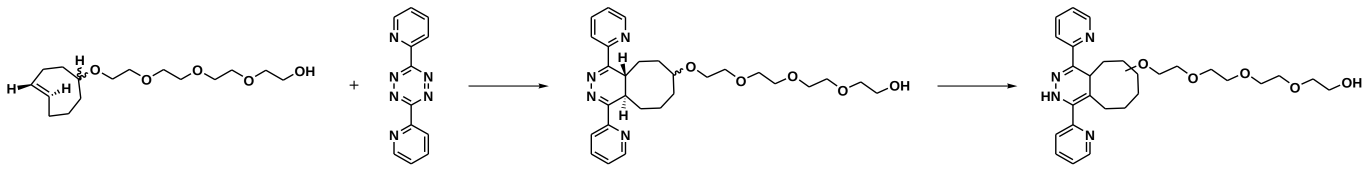

HRMS/ESI m/z $[M+H]^+$ = 511.2928 (calculated for $C_{28}H_{38}N_4O_5$: 511.2915)

- Reaction of **19** with 3,6-di(pyridin-2-yl)-1,2,4,5-tetrazine:

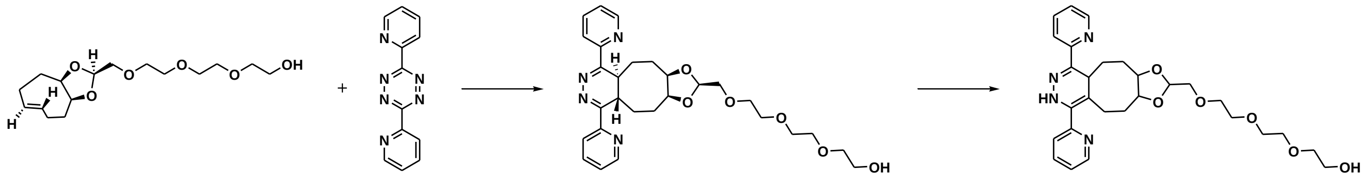

HRMS/ESI m/z $[M+H]^+$ = 525.2728 (calculated for $C_{28}H_{36}N_4O_6$: 525.2708)

- Reaction of *trans*-cyclooctenol major with 3,6-di(pyridin-2-yl)-1,2,4,5-tetrazine:

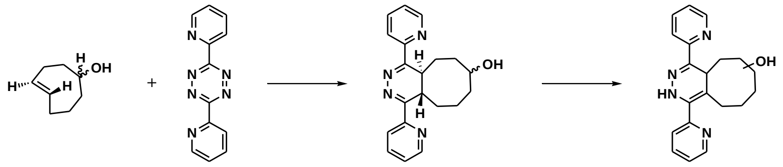

HRMS/ESI m/z $[M+H]^+$ = 335.1883 (calculated for $C_{20}H_{22}N_4O$: 335.1866)

- Reaction of *trans*-cyclooctenol minor with 3,6-di(pyridin-2-yl)-1,2,4,5-tetrazine:

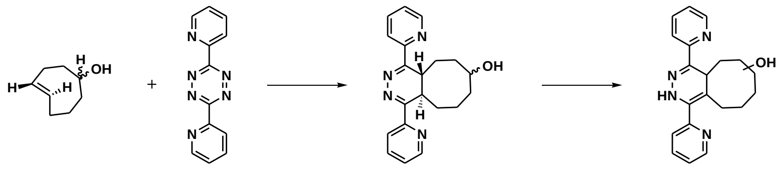

HRMS/ESI m/z $[M+H]^+$ = 335.1889 (calculated for $C_{20}H_{22}N_4O$: 335.1866)

- Reaction of BCN-OH with 3,6-di(pyridin-2-yl)-1,2,4,5-tetrazine:

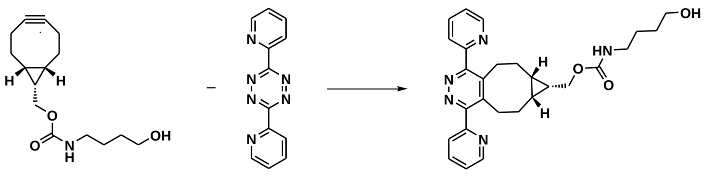

HRMS/ESI m/z $[M+H]^+$ = 474.2510 (calculated for $C_{27}H_{31}N_5O_3$: 474.2500)

5. Stability studies

Stability of compound 3 in PBS at 37 °C:

To a solution of compound **3** (2 mg; 6 µmol) in absolute ethanol (50 µL) in a tinted glass vial, PBS (2.0 mL; pH 7.4) was added. The mixture was heated at 37 °C. At selected time points (0 min, 30 min, 60 min, 120 min, 19 h) a sample (50 µL) was taken from the mixture and added to a solution of internal standard **24** (50 µL, 1 mg.mL^{-1} in ethanol) and the mixture was analyzed by HPLC (Waters Xbridge C18, 5 µm, 4.6 mm x 150 mm; flow rate: 1.0 mL.min^{-1}; UV detection: 210 nm; mobile phase: A: Na_2HPO_4, 0.025 M, pH 8.5; B: EtOH; 10% to 80% B in 20 min (linear gradient) then isocratic elution of 80% B for 5 min; or isocratic elution of 28% B for 35 min; injection volume: 50 µL; retention times are indicated in Table S2). Ratio between area (mAU*min) of **24** (and area (mAU*min) of **3** ($\mathcal{A}_{24}/\mathcal{A}_3$) was calculated for each time point. Results are displayed in Table S3. Compound **3** was stable in PBS at 37 °C for 19 h at least.

	Retention Times using gradient elution (10% to 80% B in 20 min (linear gradient) then isocratic 80% B for 5 min)	Retention times using isocratic elution (28% B for 35 min)
**24**	13.07 min	9.92 min
**3**	15.85 min	23.77 min
**18**	16.30 min	28.47 min

Table S2. Retention time of *trans*-derivative **3**, corresponding *cis*-derivative **18**, and internal standard **24**, in the HPLC elution conditions used for the experiment.

Time point	HPLC chromatogram	$\mathcal{A}_{24}/\mathcal{A}_{3}$
0 min	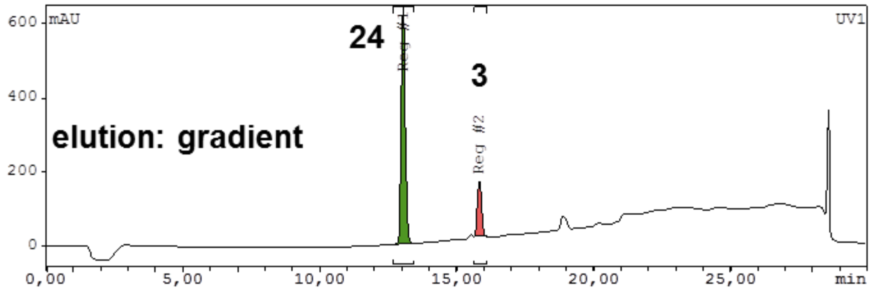	6616/1667 = 3.97
30 min	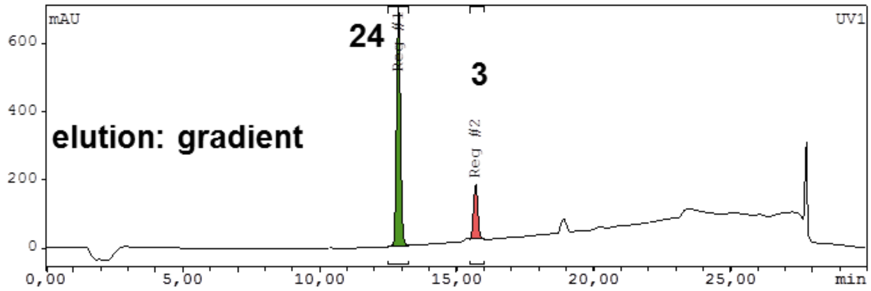	7155/1774 = 4.03
60 min	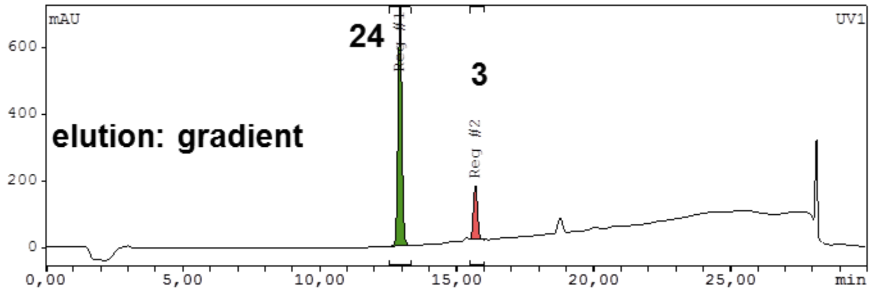	7007/1704 = 4.11
120 min	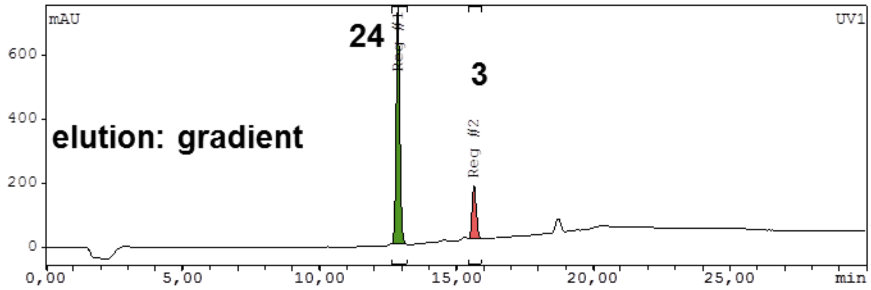	6935/1711 = 4.05
19 h	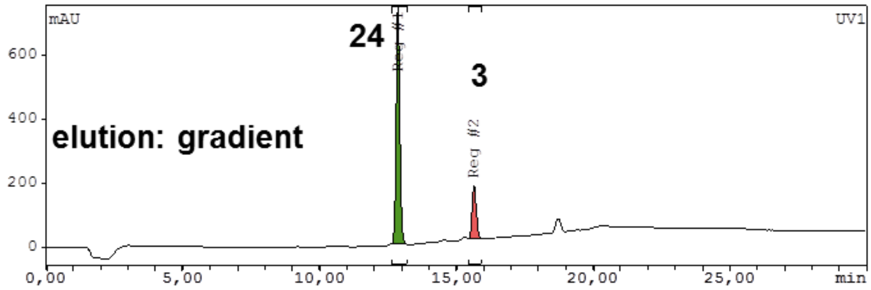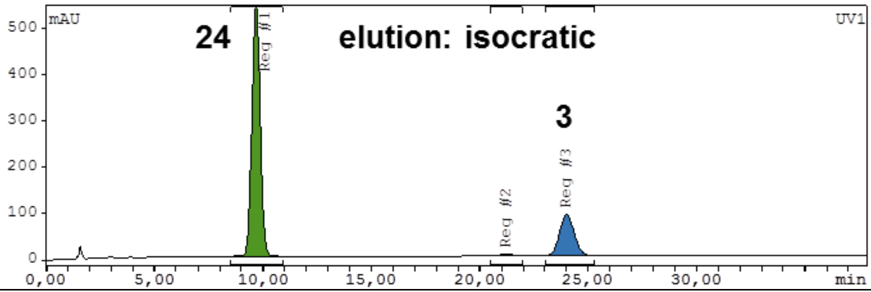	14674/3795 = 3.87

Table S3. Representative HPLC chromatograms in gradient elution conditions (10% to 80% solvent B in 20 min (linear gradient) then isocratic elution of 80% B for 5 min) or in isocratic conditions (28% B for 35 min) obtained at different time points of experiment, as well as corresponding ratio between area (mAU*min) of **24** and area (mAU*min) of **3**.

Stability of 2a in PBS at 37 °C:

To a solution of compound **2a** (4 mg; 13 µmol) in absolute ethanol (50 µL) in a tinted glass vial, PBS (1.0 mL; pH 7.4) was added. The mixture was heated at 37 °C. At selected time points (0 min, 30 min, 60 min, 120 min, 22 h) a sample (50 µL) was taken from the mixture and added to a solution of internal standard **24** (50 µL, 1 mg.mL^{-1} in ethanol) and the mixture was analyzed by HPLC (Waters Xbridge C18, 5 µm, 4.6 mm x 150 mm; flow rate: 1.0 mL.min^{-1}; UV detection: 210 nm; mobile phase: Na_2HPO_4, 0.025 M, pH 8.5/EtOH 60:40 v/v; injection volume: 50 µL; retention times are indicated in Table S4). Ratio between area (mAU*min) of **24** and area (mAU*min) of **2a** ($\mathcal{A}_{24}/\mathcal{A}_{2a}$) was calculated for each time point. Results are displayed in Table S5. Compound **2a** was stable in PBS at 37 °C for 22 h at least.

	Retention Times
**24**	3.28 min
**2a**	10.78 min
**9**	13.28 min

Table S4. Retention time of *trans*-derivative **2a**, corresponding *cis*-derivative **9**, and internal standard **24**, in the HPLC elution conditions used for the experiment.

Time point	$\mathcal{A}_{24}/\mathcal{A}_{2a}$
0 min	12126/20403 = 0.59
30 min	13643/22930 = 0.60
60 min	13633/23096 = 0.59
120 min	12172/22606 = 0.54
22 h	14794/23674 = 0.63

Table S5. Ratio between area (mAU*min) of **24** and area (mAU*min) of **2a** at different time points of experiment.

Stability of 2b in PBS at 37 °C:

To a solution of compound **2b** (2 mg; 6 µmol) in absolute ethanol (50 µL) in a tinted glass vial, PBS (500 µL; pH 7.4) was added. The mixture was heated at 37 °C. At selected time points (0 min, 30 min, 60 min, 24 h), a sample (40 µL) was taken from the mixture and added to a solution of internal standard **24** (20 µL, 1 mg.mL^{-1} in ethanol) and the mixture was analyzed by HPLC (Waters Xbridge C18, 5 µm, 4.6 mm x 150 mm; flow rate: 1.0 mL.min^{-1}; UV detection: 210 nm; mobile phase: Na_2HPO_4, 0.025 M, pH 8.5/EtOH 55:45 v/v; injection volume: 50 µL; retention times are indicated in Table S6). Ratio between area (mAU*min) of **24** and area (mAU*min) of **2b** ($\mathcal{A}_{24}/\mathcal{A}_{2b}$) was calculated for each time point. Results are displayed in Table S7. Compound **2b** was stable in PBS at 37 °C for 24 h at least.

	Retention Times
**24**	2.72 min
**2b**	14.82 min
**9**	9.02 min

Table S6. Retention time of *trans*-derivative **2b**, corresponding *cis*-derivative **9**, and internal standard **24**, in the HPLC elution conditions used for the experiment.

Time point	$\mathcal{A}_{24}/\mathcal{A}_{2b}$
0 min	10269/8363 = 1.23
30 min	9408/8099 = 1.16
60 min	9857/8119 = 1.21
24 h	9788/8892 = 1.10

Table S7. Ratio between area (mAU*min) of **24** and area (mAU*min) of **2b** at different time points of experiment.

Isomerization of compound 3 in PBS (pH 7.4) at RT:

To a solution of compound **3** (3.2 mg; 10 µmol) in absolute ethanol (25 µL) in a tinted glass vial, PBS (475 µL; pH 7.4) was added. The mixture was kept at RT for 7 days. At selected time points (0, 2 h 30, 24 h, 96 h, 168 h) a sample (10 µL) was taken from the mixture and analyzed by HPLC (Waters Xbridge C18, 5 µm, 4.6 mm x 150 mm; flow rate: 1.0 $mL.min^{-1}$; UV detection: 210 nm; mobile phase: Na_2HPO_4, 0.025 M, pH 8.5/EtOH 72:28 v/v). No isomerization was observed after 7 days.

Isomerization of compound 3 in the presence of 2-mercaptoethanol in PBS (pH 7.4) at RT:

To a solution of compound **3** (4.8 mg; 15 µmol) in absolute ethanol (25 µL) in a tinted glass vial, PBS (475 µL; pH 7.4) was added, followed by 2-mercaptoethanol (1.0 µL; 15 µmol). The mixture was kept at RT for 7 days. At selected time points (0, 1 h, 3 h, 5 h, 24 h, 96 h, 168 h) a sample (10 µL) was taken from the mixture and analyzed by HPLC (Waters Xbridge C18, 5 µm, 4.6 mm x 150 mm; flow rate: 1.0 mL/min; UV detection: 210 nm; mobile phase: Na_2HPO_4, 0.025 M, pH 8.5/EtOH 72:28 v/v). *Trans*-**3** was stable for about 5 h and then gradually isomerized to corresponding *cis*-**18** (Table S8).

Time/h	%**3**	%**18**
0	100	0
1	100	0
3	100	0
5	98	2
24	33	67
96	20	80
168	20	80

Table S8. Stability of compound **3** (30 mM) in the presence of 2-mercaptoethanol (30 mM) in PBS (pH 7.4) at RT.

Isomerization of compound 3 in rat plasma at RT:

To a solution of compound **3** (0.8 mg; 3 µmol) in absolute ethanol (25 µL) in a tinted glass vial, rat plasma (475 µL) was added. The mixture was kept at RT for 24 h. At selected time points (0, 1 h, 3 h, 5 h, 24 h) a sample (50 µL) was taken from the mixture and added to acetonitrile (50 µL). After stirring and centrifugation (3000 rpm, 5 min), the supernatant was removed, filtered (0.22 µm), and analyzed by HPLC (Waters Xbridge C18, 5 µm, 4.6 mm x 150 mm; flow rate: 1.0 $mL.min^{-1}$; UV detection: 210 nm; mobile phase: Na_2HPO_4, 0.025 M, pH 8.5/EtOH 72:28 v/v; injection volume: 20 µL). Results were plotted using GraphPad Prism 5 software (Figure S3).

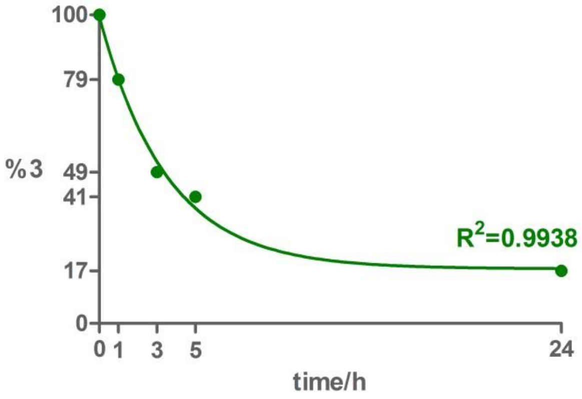

Figure S3. Stability profile of compound **3** in rat plasma at RT.

Isomerization of compound 3 in the presence of human serum albumin, a metal-containing biomolecule, at RT:

To a solution of compound **3** (0.4 mg; 1 µmol) in absolute ethanol (5 µL) in a tinted vial, PBS (45 µL; pH 7.4) was added followed by human serum albumin (20 mg.mL^{-1}, 200 µL). The mixture was kept at RT for 24 h. At selected time points (0, 30 min, 1 h, 6 h, 24 h) a sample (50 µL) was taken from the mixture and added to acetonitrile (50 µL). After stirring and centrifugation (3000 rpm, 5 min), the supernatant was removed, filtered (0.22 µm), and analyzed by HPLC (Waters Xbridge C18, 5 µm, 4.6 mm x 150 mm; flow rate: 1.0 mL.min^{-1}; UV detection: 210 nm; mobile phase: Na_2HPO_4, 0.025 M, pH 8.5/EtOH 72:28 v/v; injection volume: 20 µL). No isomerization was observed after 24 h.

6. Radiosyntheses

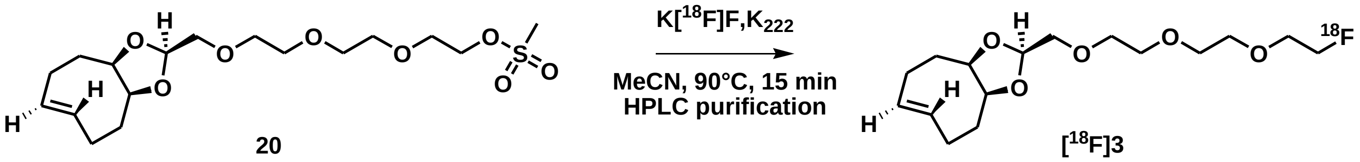

Preparation of K[^{18}F]F,K$_{222}$: [^{18}F]F$^-$ in aqueous solution H_2[^{18}O]O (Activity at end-of-bombardment: 20-35 GBq) was trapped on a QMA cartridge (CO_3^{2-} form). The radioactivity was eluted in the reactor as a K[^{18}F]F,K$_{222}$ complex, using a mixture of potassium carbonate (1.2 mg) and Kryptofix (K$_{222}$, 13.9 mg) in solution (acetonitrile/water 98.5:2.5 v/v, 750 µL). Solvents were evaporated at 110 °C for 6 min, under helium flow. A second and a third azeotropic drying were repeated after addition of anhydrous acetonitrile (2 x 500 µL), at 110 °C for 4 min, under helium flow. During the preparation and drying of the K[^{18}F]F,K$_{222}$ complex, the light in the shielded cell was turned off to avoid degradation of the precursor **20**.

Synthesis of **[^{18}F]3**: Mesylate precursor **20** (0.3 mg/300 µL anhydrous acetonitrile) was added in the reactor. The mixture was heated at 90 °C for 15 min. Back to 35 °C, the reaction mixture was diluted with HPLC eluent (0.6 mL) and injected on an HPLC column, using 0.6 mL of water (Waters Xbridge C18, 5 µm, 4.6 mm x 150 mm; flow rate: 1.0 mL.min^{-1}; mobile phase: Na_2HPO_4, 0.025 M, pH 8.5/EtOH; 72:28 v/v; t_R = 17.4 min). The collected solution was then diluted with saline for biological experiments. **[^{18}F]3**

was obtained in 60 min, with 12% overall radiochemical yield (decay-corrected). Radiochemical purity (RCP) was determined by analytical HPLC (VWR-Hitachi LaChrom Elite system; Waters Xbridge C18 column, 5 µm, 4.6 mm x 150 mm; flow rate: 1.0 mL.min^{-1}; UV detection 210 nm; mobile phase: Na_2HPO_4, 0.025 M, pH 8.5/EtOH 72:28 v/v; t_R = 16.42 min; Figure S4) and was >99%. Specific activity was determined by analytical HPLC (Waters system; Waters XSelect HSS PFP column, 3.5 µm, 4.6 mm x 150 mm; flow rate: 1.0 mL.min^{-1}; UV detection: 210 nm; mobile phase: Na_2HPO_4, 5 mM, pH 7.5/MeCN 78:22 v/v; t_R = 29.95 min) and was 70-188 GBq.µmol^{-1}. **[^{18}F]3** was radiochemically stable (RCP>99%) for at least 2 h after preparation. log *D* = 1.25 ± 0.01.

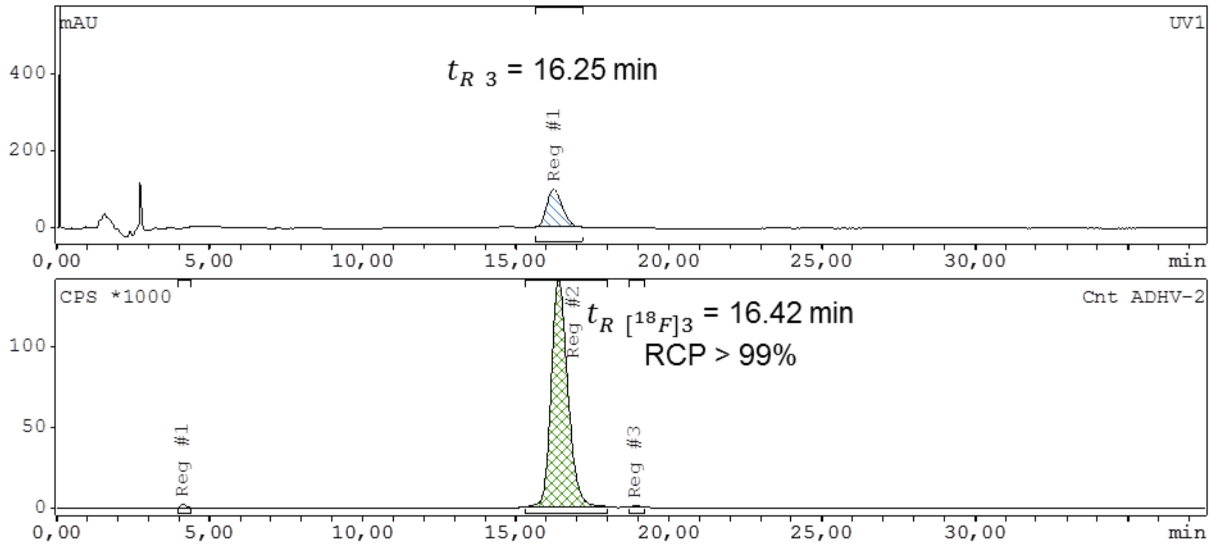

Figure S4: Quality control of **[^{18}F]3** by analytical HPLC demonstrated a RCP>99% and confirmed the identity of the collected product, by coinjection with non-radioactive compound **3**. *Top: UV-chromatogram, bottom: radio-chromatogram.*

7. Stability studies of [^{18}F]3 in PBS and rat plasma

Stability of [^{18}F]3 in PBS at 37 °C:

A solution of **[^{18}F]3** (100 µL) in HPLC eluent (Na_2HPO_4, pH 8.5, 0.025 M/EtOH 72:28 v/v) was added to PBS (pH 7.4, 400 µL), in a tinted glass vial. The mixture was incubated at 37 °C during 2 h. At selected time points (0, 60 and 120 min), a sample (50 µL) was taken from the mixture and analyzed by HPLC (Waters Xbridge C18, 5 µm, 4.6 mm x 150 mm; flow rate: 1.0 mL.min^{-1}; UV detection: 210 nm; mobile phase: Na_2HPO_4, 0.025 M, pH 8.5/EtOH 72:28 v/v; injection volume: 40 µL). Results are presented in Table S9 and Figure S5.

Stability of [^{18}F]3 in rat plasma at 37 °C:

A solution of **[^{18}F]3** (100 µL) in HPLC eluent (Na_2HPO_4, 0.025 M, pH 8.5/EtOH 72:28 v/v) was added to plasma obtained from rat serum (400 µL), in a tinted glass vial. The mixture was incubated at 37 °C during 2 h. At selected time points (0, 30, 60 and 120 min), a sample (50 µL) was taken from the mixture and added to acetonitrile (50 µL). After stirring and centrifugation (3000 rpm, 5 min), the supernatant was removed, filtered (0.22 µm), and analyzed by HPLC (Waters Xbridge C18, 5 µm, 4.6 mm x 150 mm; flow rate: 1.0 mL.min^{-1}; UV detection: 210 nm; mobile phase: Na_2HPO_4, 0.025 M, pH 8.5/EtOH 72:28 v/v;

injection volume: 30 µL). Results were plotted using GraphPad Prism 5 software (R^2>0.9905), and are presented in Table S9 and Figure S5.

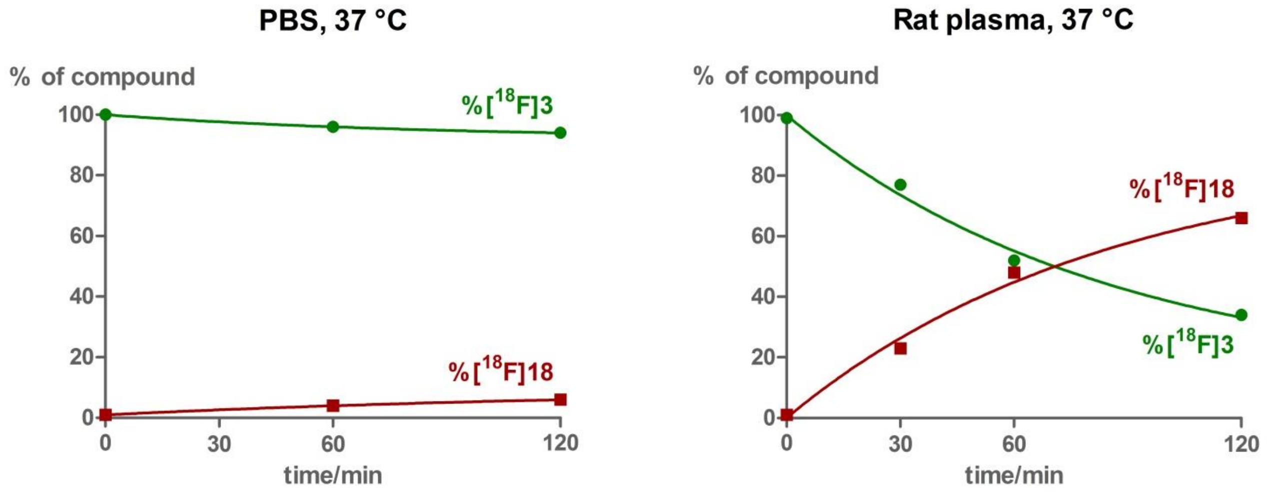

Figure S5. Stability of **[^{18}F]3** in PBS and rat plasma at 37 °C

Time (min)	%[^{18}F]3		%[^{18}F]18	
	PBS	rat plasma	PBS	rat plasma
**0**	>99	>99	<1	<1
**30**	n.d.[a]	77	n.d.[a]	23
**60**	96	52	4	48
**120**	94	34	6	66

[a]not determined

Table S9. Stability of **[^{18}F]3** in PBS and rat plasma at 37 °C.

8. Biodistribution after injection of [^{18}F]3 in healthy mice and radiometabolite analyses

	%ID ± SD			
	**2 min**	**10 min**	**30 min**	**60 min**
blood	12.8 ± 1.7	9.7 ± 0.4	7.4 ± 0.8	6.5 ± 0.2
bone	4.4 ± 0.7	3.6 ± 0.5	3.6 ± 0.4	3.9 ± 0.4
muscle	47.2 ± 5.2	36.4 ± 1.8	29.8 ± 2.0	23.6 ± 1.2
brain	1.1 ± 0.2	0.6 ± 0.1	0.5 ± 0.0	0.5 ± 0.0
kidneys	4.1 ± 0.3	3.3 ± 0.3	2.5 ± 0.2	2.4 ± 0.9
bladder	0.3 ± 0.1	6.6 ± 1.1	20.2 ± 3.3	31.8 ± 1.8
liver	13.9 ± 1.0	11.1 ± 0.6	8.5 ± 0.8	6.5 ± 0.3
intestines	10.9 ± 1.8	11.3 ± 1.3	11.6 ± 0.8	11.6 ± 0.2
stomach	1.2 ± 0.3	1.0 ± 0.1	1.0 ± 0.0	0.8 ± 0.1
spleen	0.4 ± 0.1	0.3 ± 0.0	0.3 ± 0.1	0.2 ± 0.0
pancreas	0.8 ± 0.4	0.7 ± 0.2	0.5 ± 0.1	0.3 ± 0.1
lungs	1.3 ± 0.5	0.8 ± 0.2	0.6 ± 0.2	0.7 ± 0.3
heart	0.7 ± 0.1	0.5 ± 0.1	0.4 ± 0.0	0.3 ± 0.1
carcass	58.2 ± 4.0	57.6 ± 2.0	49.3 ± 2.9	39.9 ± 1.9

Table S10. Biodistribution of radioactivity after injection of **[^{18}F]3** in healthy NMRI mice at 2, 10, 30 and 60 min p.i.

Data are expressed as percentage of injected dose (%ID) ± standard deviation (SD) (N = 3).

	SUV_w ± SD			
	**2 min**	**10 min**	**30 min**	**60 min**
blood	1.8 ± 0.2	1.4 ± 0.1	1.1 ± 0.1	0.9 ± 0.0
bone	0.4 ± 0.1	0.3 ± 0.0	0.3 ± 0.0	0.3 ± 0.0
muscle	1.2 ± 0.1	0.9 ± 0.1	0.7 ± 0.1	0.6 ± 0.0
kidneys	2.9 ± 0.4	2.5 ± 0.1	1.7 ± 0.2	1.6 ± 0.6
liver	3.4 ± 0.7	2.6 ± 0.1	1.9 ± 0.1	1.7 ± 0.1
spleen	1.6 ± 0.2	1.3 ± 0.1	1.1 ± 0.1	0.9 ± 0.1
pancreas	1.8 ± 0.5	1.3 ± 0.1	0.9 ± 0.1	0.7 ± 0.1
lungs	2.0 ± 0.3	1.5 ± 0.1	1.0 ± 0.1	0.9 ± 0.1
heart	1.9 ± 0.2	1.3 ± 0.1	1.0 ± 0.1	0.8 ± 0.1
cerebrum	1.3 ± 0.2	0.7 ± 0.1	0.6 ± 0.1	0.6 ± 0.1
cerebellum	1.3 ± 0.2	0.7 ± 0.1	0.6 ± 0.0	0.5 ± 0.0

Table S11. Biodistribution of radioactivity after injection of **[^{18}F]3** in healthy NMRI mice at 2, 10, 30 and 60 min p.i.

Data are expressed as standardized uptake value (SUV_w) ± standard deviation (SD) (N = 3).

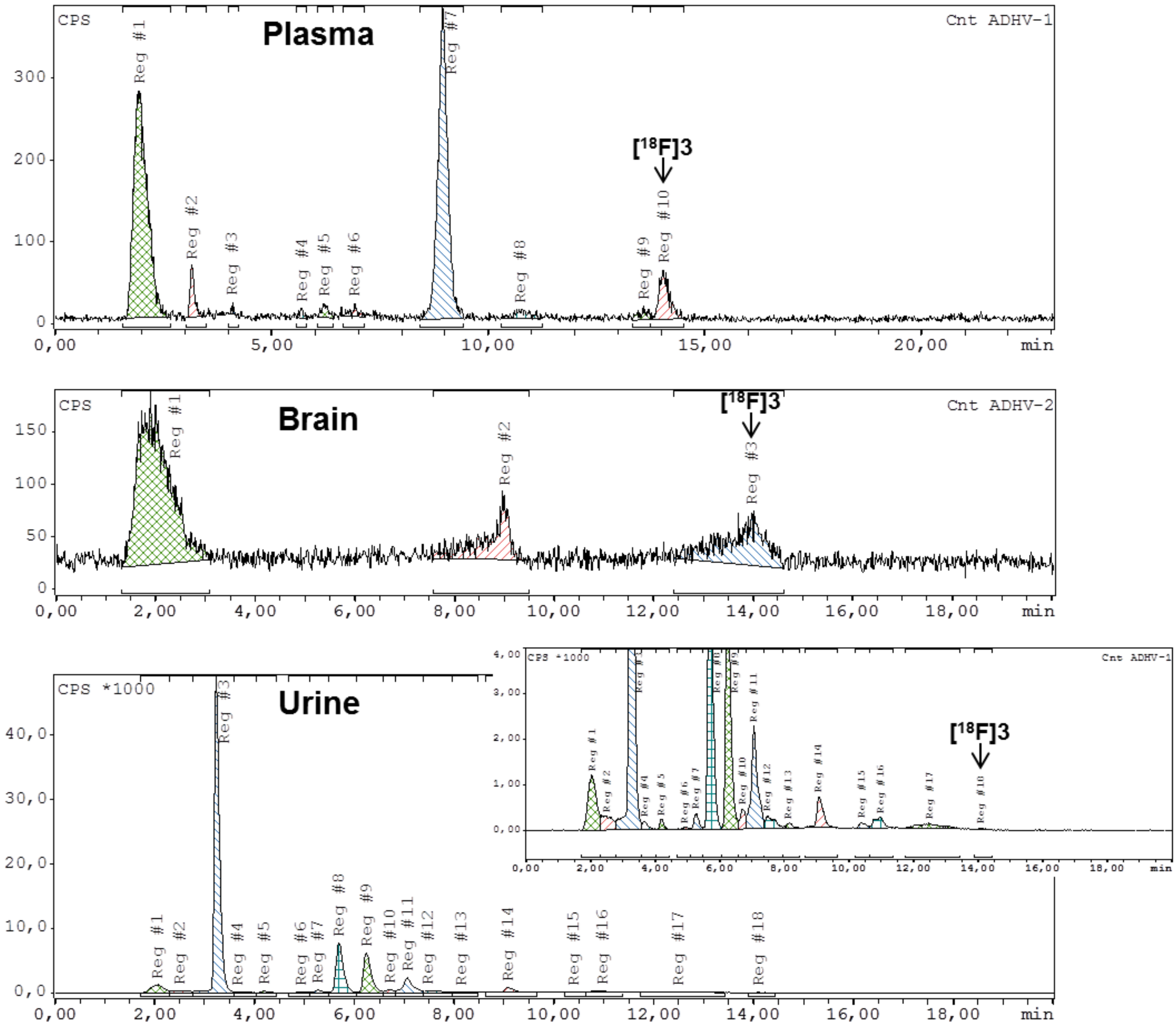

Figure S6. Representative radiochromatograms of plasma, brain and urine samples 15 min after i.v. injection of **[^{18}F]3** (~ 9 MBq) in healthy mice.

9. *In vitro* pretargeting

Radiosynthesis of [^{18}F]22:

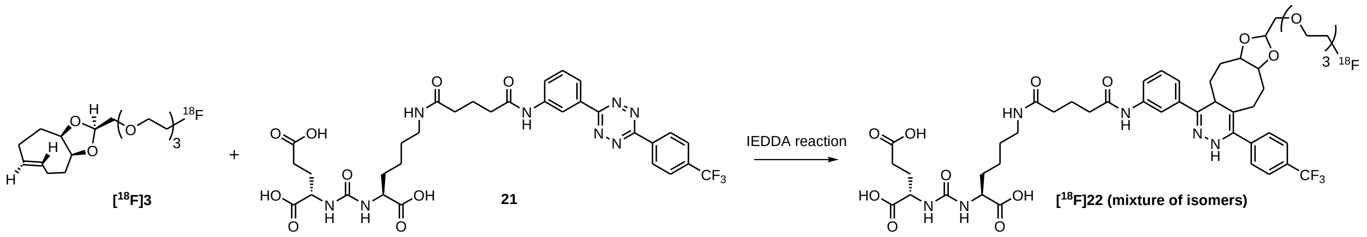

A solution of **[^{18}F]3** (500 µL, 30 MBq) in HPLC eluent (Na_2HPO_4, 0.025 M, pH 8.5/EtOH 72:28 v/v) was mixed with a solution of **21** (20 µL, 1 mM in PBS pH 7.4). After 2 min, the solution was purified by HPLC (Waters Xbridge C18, 5 µm, 4.6 mm x 150 mm; flow rate: 1.0 mL.min^{-1}; UV detection: 254 nm; mobile phase: Na_2HPO_4, 5 mM, pH 7.5/MeCN 70:30 v/v; t_R = 8.83 min and 9.45 min, Figure S7). **[^{18}F]22** (mixture of isomers) was produced in 12 min with 67% radiochemical yield (decay-corrected). The identity of the collected products was confirmed by LC-HRMS (HRMS/ESI m/z $[M+H]^+$ = 1023.4351 (calculated for $C_{48}H_{62}F_4N_6O_{14}$: 1023.4333). LC-HRMS also demonstrated that compound **21** was not collected during the purification.
It has to be noted that the click reaction is quantitative, as **[^{18}F]3** is totally consumed during the reaction. This was confirmed by HPLC analysis of crude reaction mixture using gradient conditions, with the gradient needed to elute **[^{18}F]3** (Waters Xbridge C18, 5 µm, 4.6 mm x 150 mm; flow rate: 1.0 mL.min^{-1}; UV detection: 210 nm; mobile phase: solvent A: Na_2HPO_4 5 mM pH 7.5; solvent B: acetonitrile; gradient 15% B to 50% in 20 min then isocratic elution with 50% B for 5 min, Figure S8).

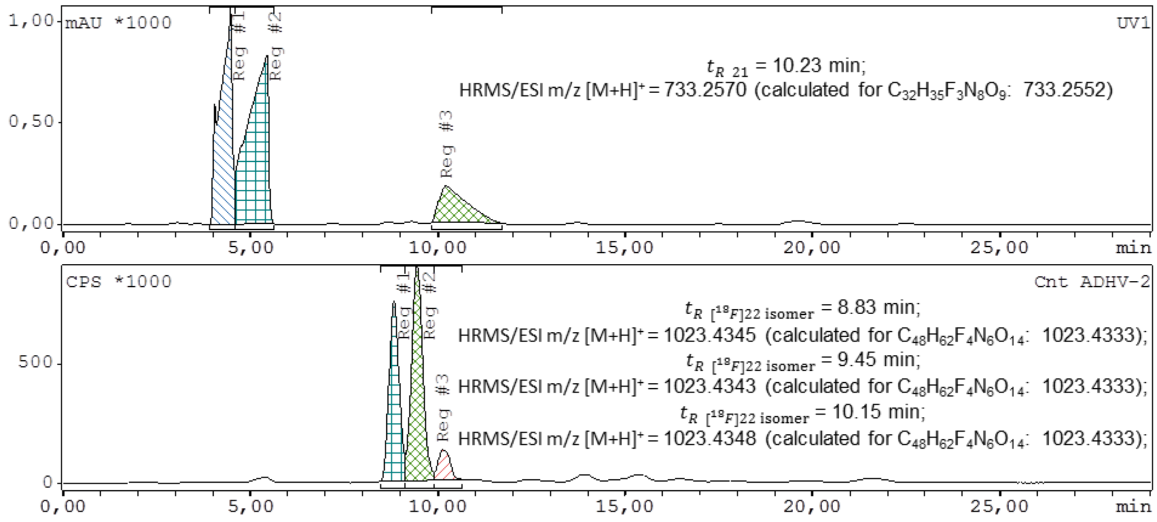

Figure S7. UV(top) and radio(bottom)-chromatograms of crude reaction mixture of **[^{18}F]22** radiosynthesis.
<u>HPLC conditions:</u> *Waters Xbridge C18, 5 µm, 4.6 mm x 150 mm; flow rate: 1.0 mL.min^{-1}; UV detection: 254 nm; mobile phase: Na_2HPO_4, 5 mM, pH 7.5/acetonitrile 70:30 v/v*
Molecular ion mass values were obtained by LC-HRMS analysis of the isolated peaks.

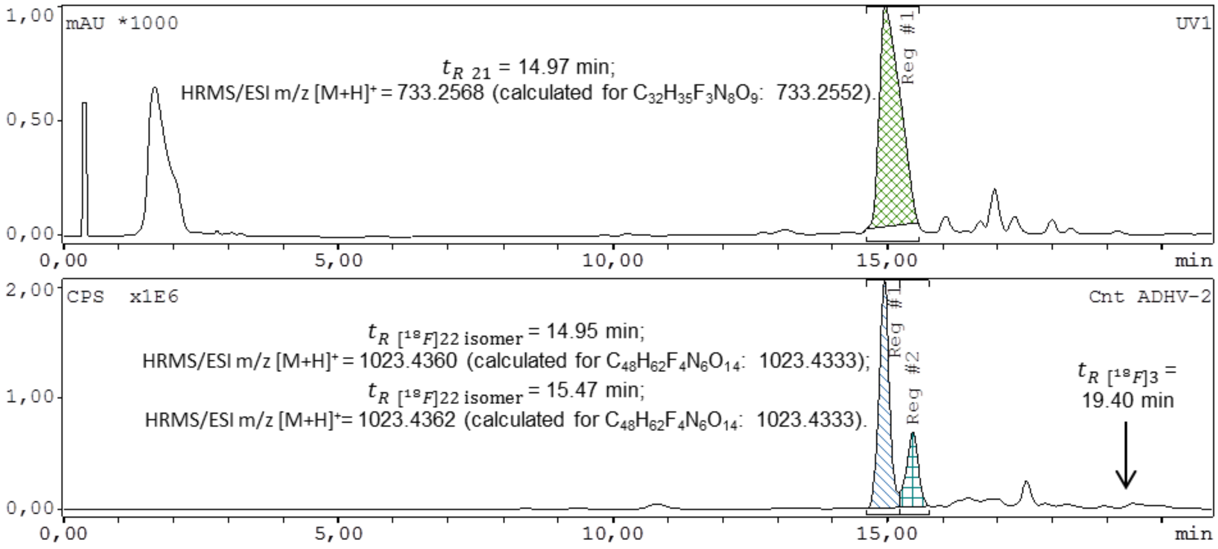

Figure S8. UV(top) and radio(bottom)-chromatograms of crude reaction mixture of **[^{18}F]22** radiosynthesis.

<u>HPLC conditions:</u> *Waters Xbridge C18, 5 µm, 4.6 mm x 150 mm; flow rate: 1.0 mL.min^{-1}; UV detection: 210 nm; mobile phase: solvent A: Na_2HPO_4 5 mM pH 7.5; solvent B: acetonitrile; gradient 15% B to 50% in 20 min then isocratic elution with 50% B for 5 min.*

Molecular ion mass values were obtained by LC-HRMS analysis of the isolated peaks.

Incubation of tumor slices and autoradiography:

Pretargeting with compound 21 and [^{18}F]3: Prostate tumor slices (LNCaP and PC-3 cells) were defrosted (N=4/experiment) and gently dried using a heat-gun. The slices were then pre-incubated in PBS (0.01 M, pH 7.4) at RT for 10 min. After a gentle drying, the slices were incubated either with a solution of **21** (1 µM in PBS 0.01 M, pH 7.4; 200 µL) or with a mixture of compound **21** (1 µM) and blocking agent 2-(phosphonomethyl)pentane-1,5-dioic acid (2-PMPA; 100 µM) in PBS (0.01 M, pH 7.4; 200 µL), at RT for 10 min. The slices were then washed at RT using the following procedure:

- 1 min in a solution of PBS (0.01 M, pH 7.4) containing 0.3% bovine serum albumin (BSA);
- 2 min in a solution of 70% ethanol in PBS (0.01 M, pH 7.4), containing 0.3% BSA;
- 1 min in a solution of 30% ethanol in PBS (0.01 M, pH 7.4), containing 0.3% BSA;
- 1 min in a solution of PBS (0.01 M, pH 7.4) containing 0.3% BSA.

The slices were then gently dried and incubated with a solution of **[^{18}F]3** (1.5 MBq.mL^{-1} in PBS 0.01 M, pH 7.4; 200 µL) at RT for 10 min. The slices were then washed at RT using the following procedure:

- 1 min in a solution of PBS (0.01 M, pH 7.4) containing 0.3% BSA;
- 2 min in a solution of 70% ethanol in PBS (0.01 M, pH 7.4), containing 0.3% BSA;
- 1 min in a solution of 30% ethanol in PBS (0.01 M, pH 7.4), containing 0.3% BSA;
- 1 min in a solution of PBS (0.01 M, pH 7.4) containing 0.3% BSA;
- a few seconds in water.

The slices were then gently dried and exposed overnight to a storage phosphor screens, in a cassette. For quantification, **[^{18}F]3** (1.5 MBq.mL^{-1} in PBS 0.01 M, pH 7.4) was spotted on an iTLC strip (10 and 20 µL) and exposed overnight in the same cassette.

Direct incubation with [^{18}F]22: Prostate tumor slices (LNCaP and PC-3 cells) were defrosted (N=4/experiment) and gently dried using a heat-gun. The slices were then pre-incubated in PBS (0.01 M, pH 7.4) at RT for 10 min. After a gentle drying, the slices were incubated either with a solution of **[^{18}F]22** (1.5 MBq.mL^{-1} in PBS 0.01 M, pH 7.4; 200 µL) or with a mixture of **[^{18}F]22** (1.5 MBq.mL^{-1}) and blocking agent 2-PMPA (100 µM) in PBS (0.01 M, pH 7.4; 200 µL), at RT for 10 min. The slices were then washed at RT using the following procedure:

- 1 min in a solution of PBS (0.01 M, pH 7.4) containing 0.3% BSA;
- 2 min in a solution of 70% ethanol in PBS (0.01 M, pH 7.4), containing 0.3% BSA;
- 1 min in a solution of 30% ethanol in PBS (0.01 M, pH 7.4), containing 0.3% BSA;
- 1 min in a solution of PBS (0.01 M, pH 7.4) containing 0.3% BSA.
- a few seconds in water.

The slices were then gently dried and exposed overnight to a storage phosphor screens, in a cassette. For quantification, **[^{18}F]3** (1.5 MBq.mL^{-1} in PBS 0.01 M, pH 7.4) was spotted on an iTLC strip (10 and 20 µL) and exposed overnight in the same cassette.

Incubation with [^{18}F]3: Prostate tumor slices (LNCaP and PC-3 cells) were defrosted (N=2-3/experiment) and gently dried using a heat-gun. The slices were then pre-incubated in PBS (0.01 M, pH 7.4) at RT for 10 min. After a gentle drying, the slices were incubated with a solution of **[^{18}F]3** (1.5 MBq.mL^{-1} in PBS 0.01 M, pH 7.4; 200 µL) at RT for 10 min. The slices were then washed at RT using the following procedure:
- 1 min in a solution of PBS (0.01 M, pH 7.4) containing 0.3% BSA;
- 2 min in a solution of 70% ethanol in PBS (0.01 M, pH 7.4), containing 0.3% BSA;
- 1 min in a solution of 30% ethanol in PBS (0.01 M, pH 7.4), containing 0.3% BSA;
- 1 min in a solution of PBS (0.01 M, pH 7.4) containing 0.3% BSA;
- a few seconds in water.

The slices were then gently dried and exposed overnight to a storage phosphor screens, in a cassette. For quantification, **[^{18}F]3** (1.5 MBq.mL^{-1} in PBS 0.01 M, pH 7.4) was spotted on an iTLC strip (10 and 20 µL) and exposed overnight in the same cassette.

Results

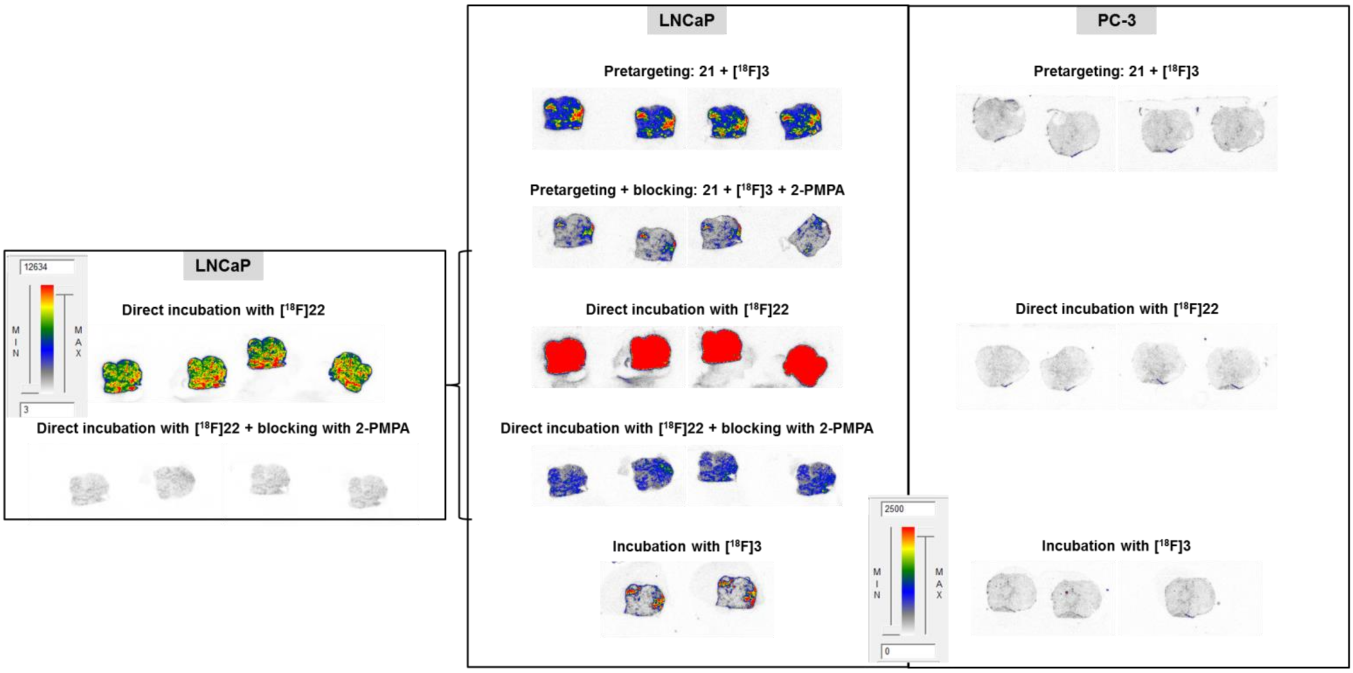

Figure S9. Autoradiography revealed that the binding to LNCaP tumor slices (expressing PSMA receptors) subjected to pretargeting with compound **21** followed by addition of **[^{18}F]3** was PSMA-specific but inferior to the binding of **[^{18}F]22** directly incubated with the slices. No significant binding was observed in PC-3 cells (negative control).

10. *In vivo* microPET imaging

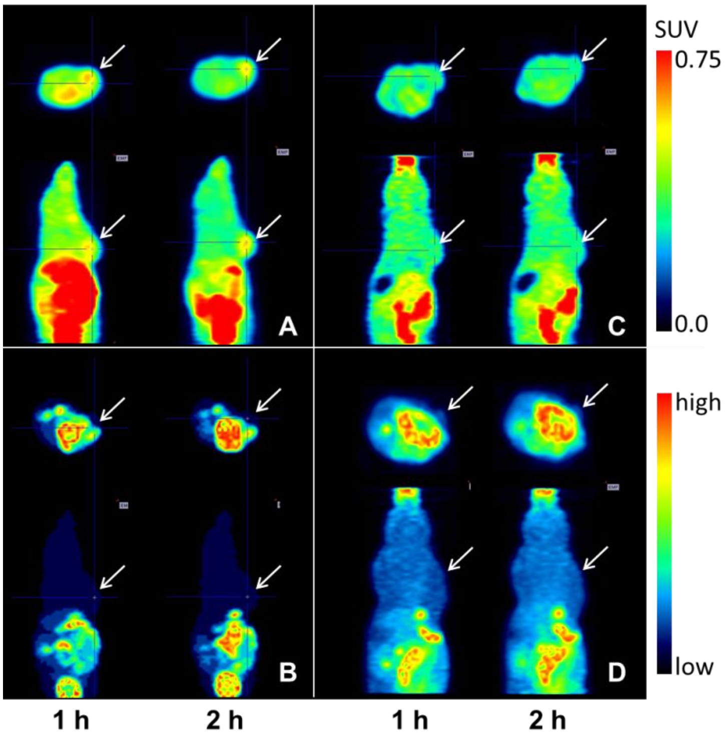

Figure S10. *In vivo* PET imaging and biodistribution of radioactivity after injection of bioorthogonal **[^{18}F]3**, a selective tracer for tetrazine-tagged tumors, in LNCaP prostate tumor-bearing nude mice. (A),(B) PET images 1 h and 2 h p.i. of **[^{18}F]3** (11 MBq) in a mouse bearing a LNCaP tumor treated with tetrazine **21** (50 µg). (A) Tomographic slices bisecting the tumor and (B) Maximum Intensity Projection for the same mouse. (C),(D) Control PET images 1 h and 2 h p.i. of **[^{18}F]3** (9 MBq) in a mouse bearing a non-treated LNCaP tumor. (C) Tomographic slices bisecting the tumor and (D) Maximum intensity projection for the same mouse. Tumors are indicated by arrows.

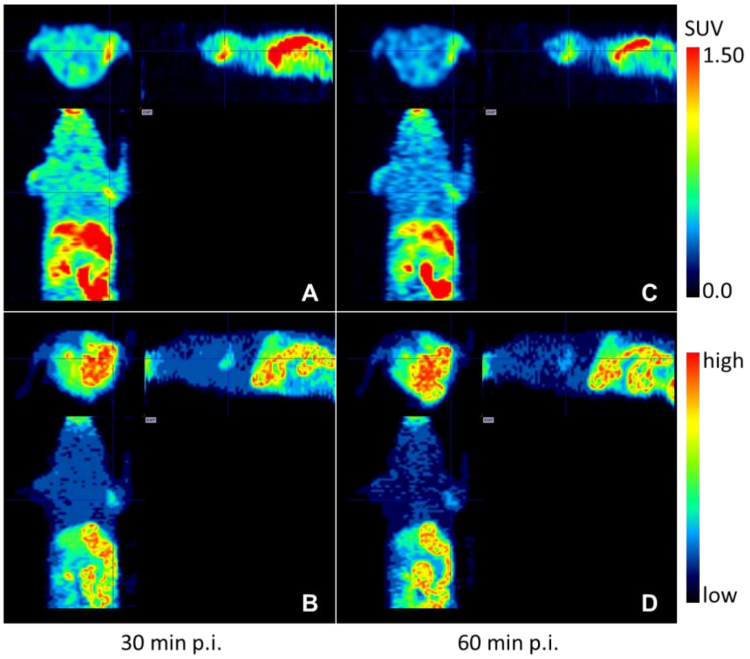

Figure S11. *In vivo* PET images 30 min and 60 min p.i. of **[^{18}F]3** (11 MBq) in an healthy NMRI mouse which received an intramuscular injection of tetrazine **21** (50 µg) in the muscle on the right shoulder, and saline with 10% dimethyl sulfoxide in the muscle on the left shoulder. (A),(C) Tomographic slices and (B),(D) Maximum intensity projection for the same mouse.

11. ^{1}H-NMR and ^{13}C-NMR spectra of representative compounds

(*Z*)-15-(cyclooct-4-en-1-yloxy)-3,3-diisopropyl-2-methyl-4,7,10,13-tetraoxa-3-silapentadecane 5

^{1}H NMR ($CDCl_3$):

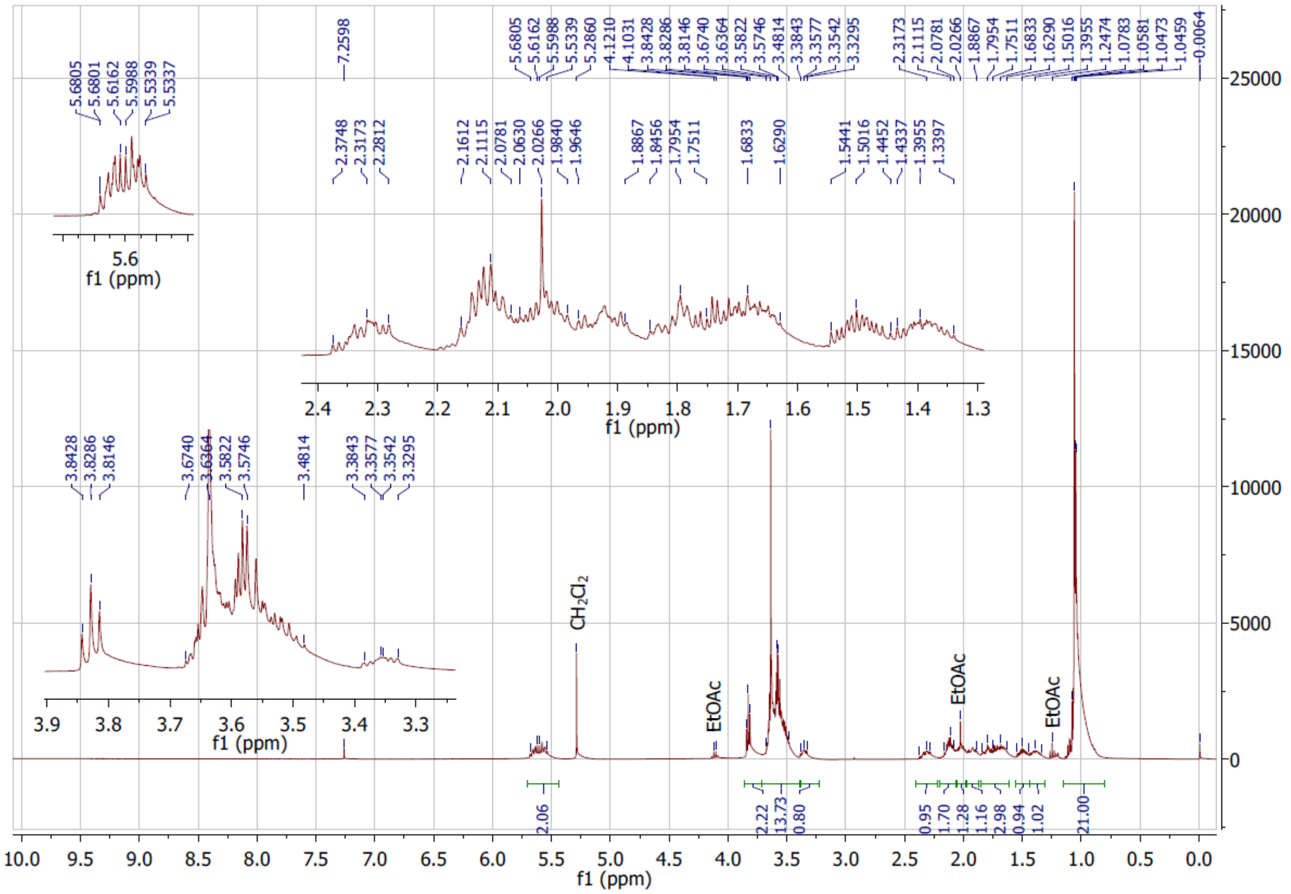

^{13}C NMR ($CDCl_3$):

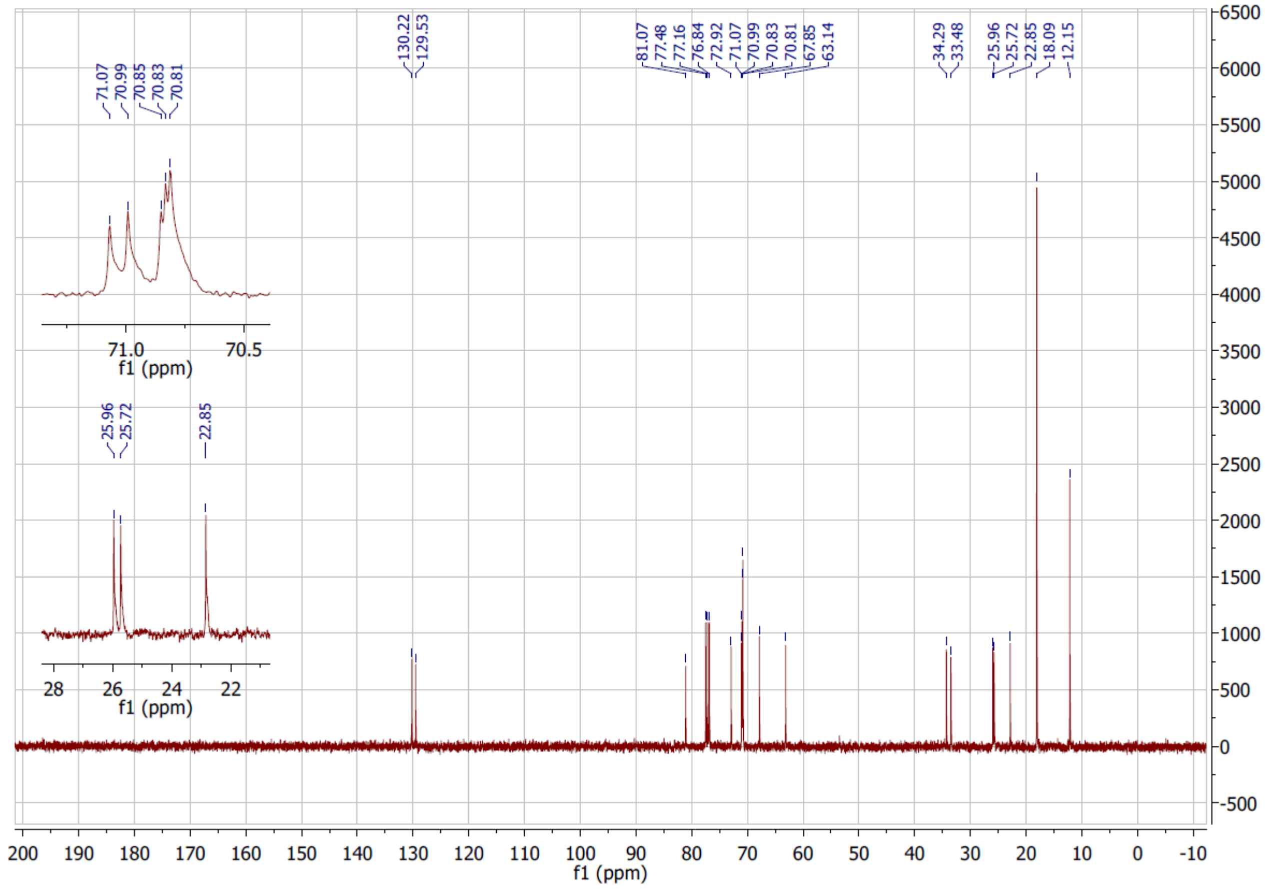

(*Z*)-2-(2-(2-(2-(cyclooct-4-en-1-yloxy)ethoxy)ethoxy)ethoxy)ethanol 6

^{1}H NMR ($CDCl_3$):

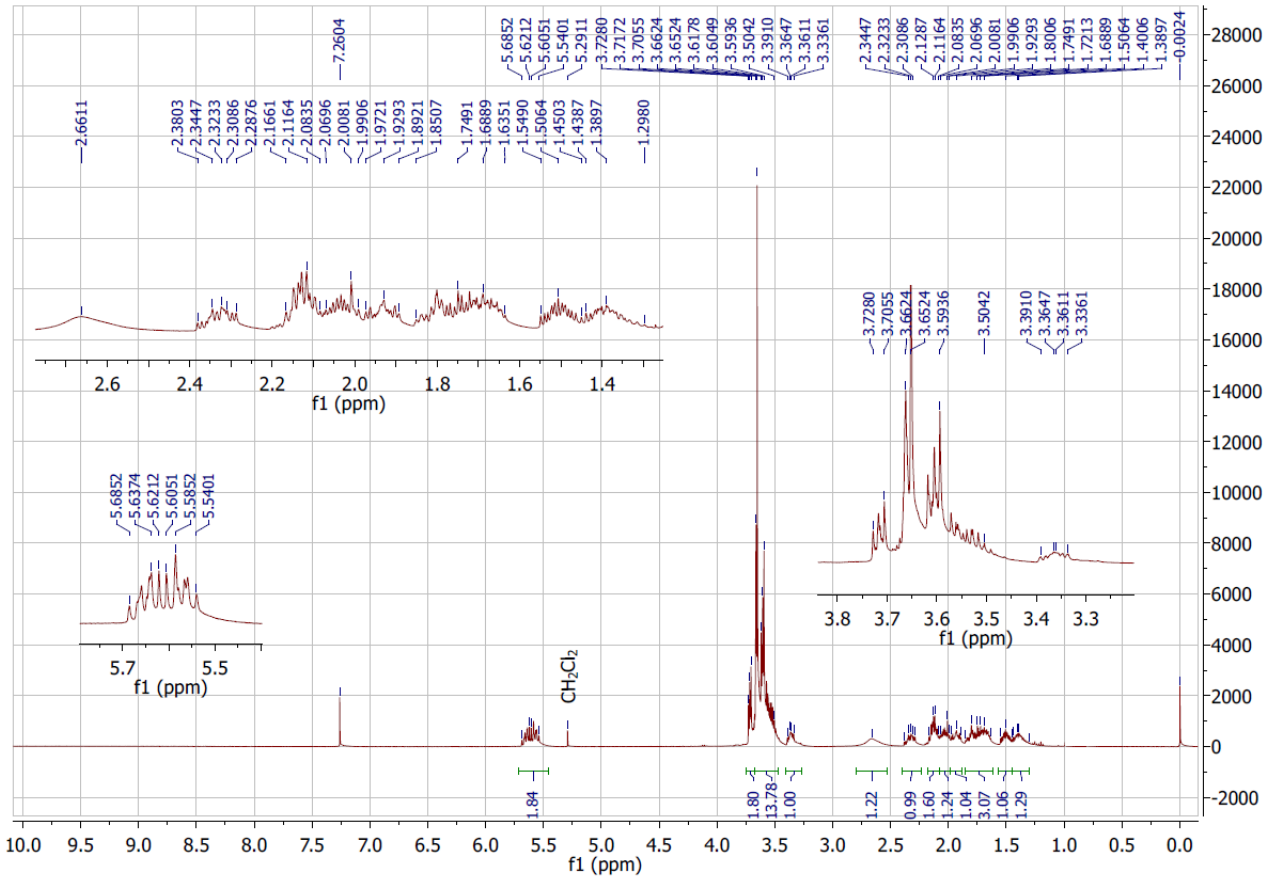

^{13}C NMR ($CDCl_3$):

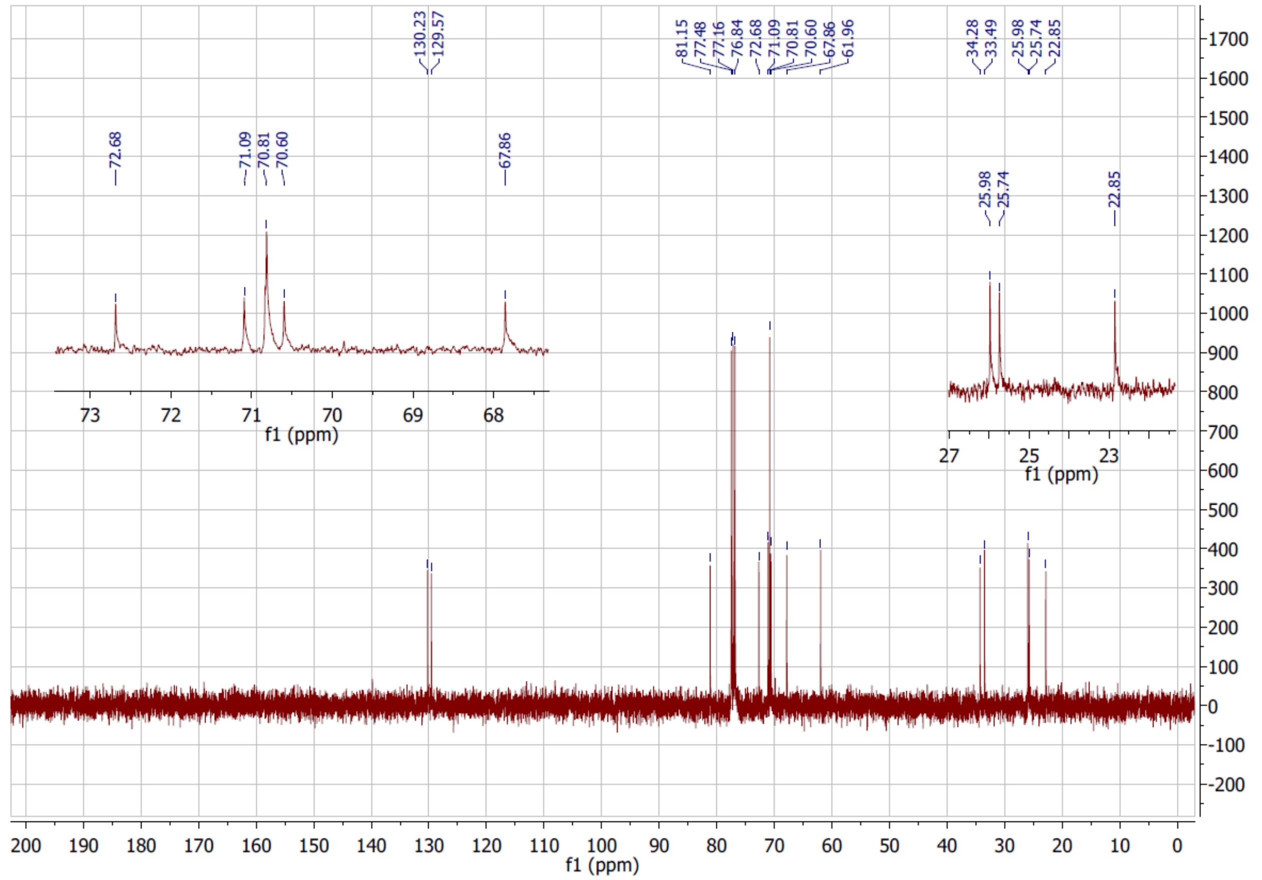

2-(2-(2-(2-((*Z*)-cyclooct-4-enyloxy)ethoxy)ethoxy)ethoxy)ethyl methanesulfonate 8

^{1}H NMR ($CDCl_3$):

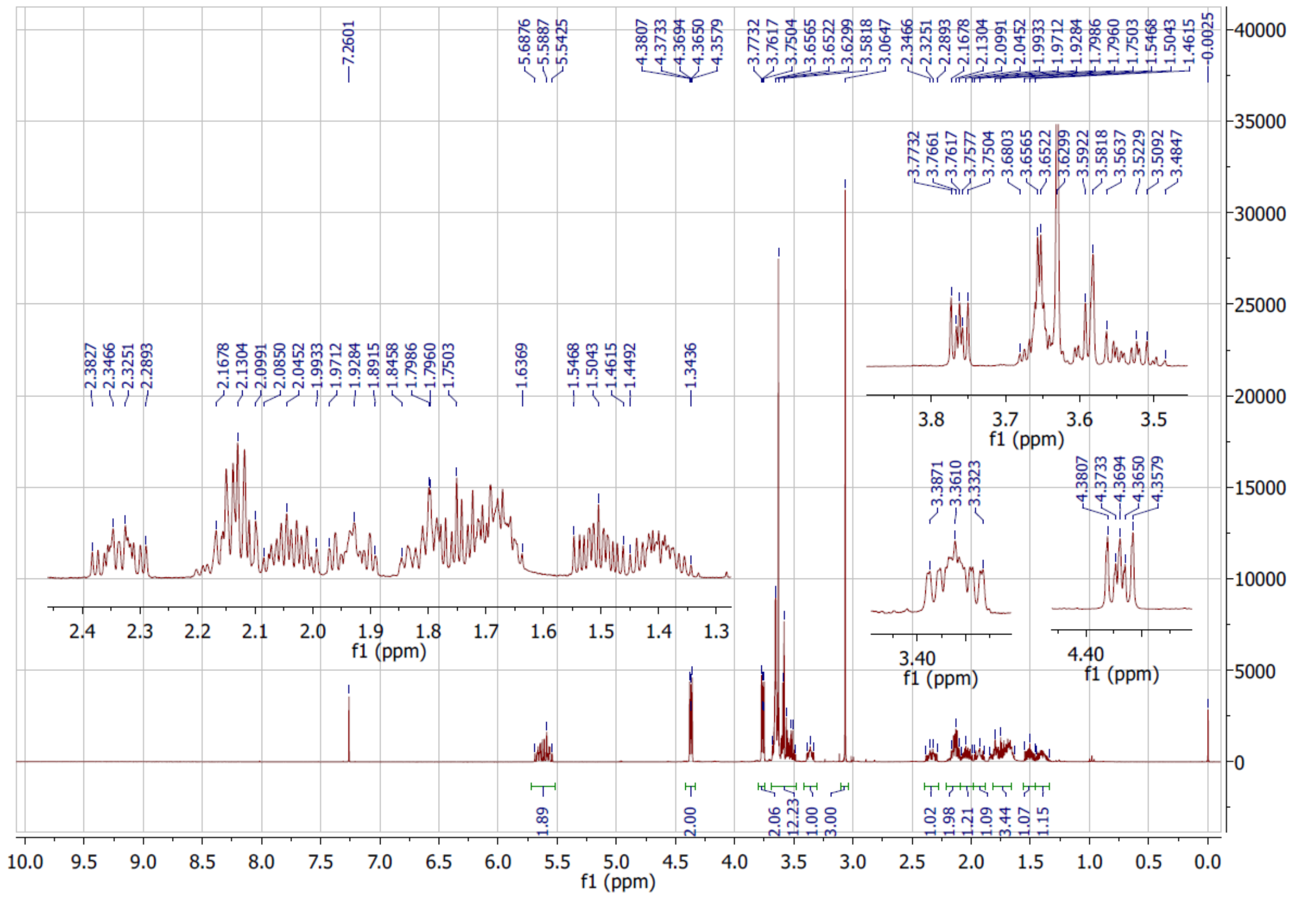

^{13}C NMR ($CDCl_3$):

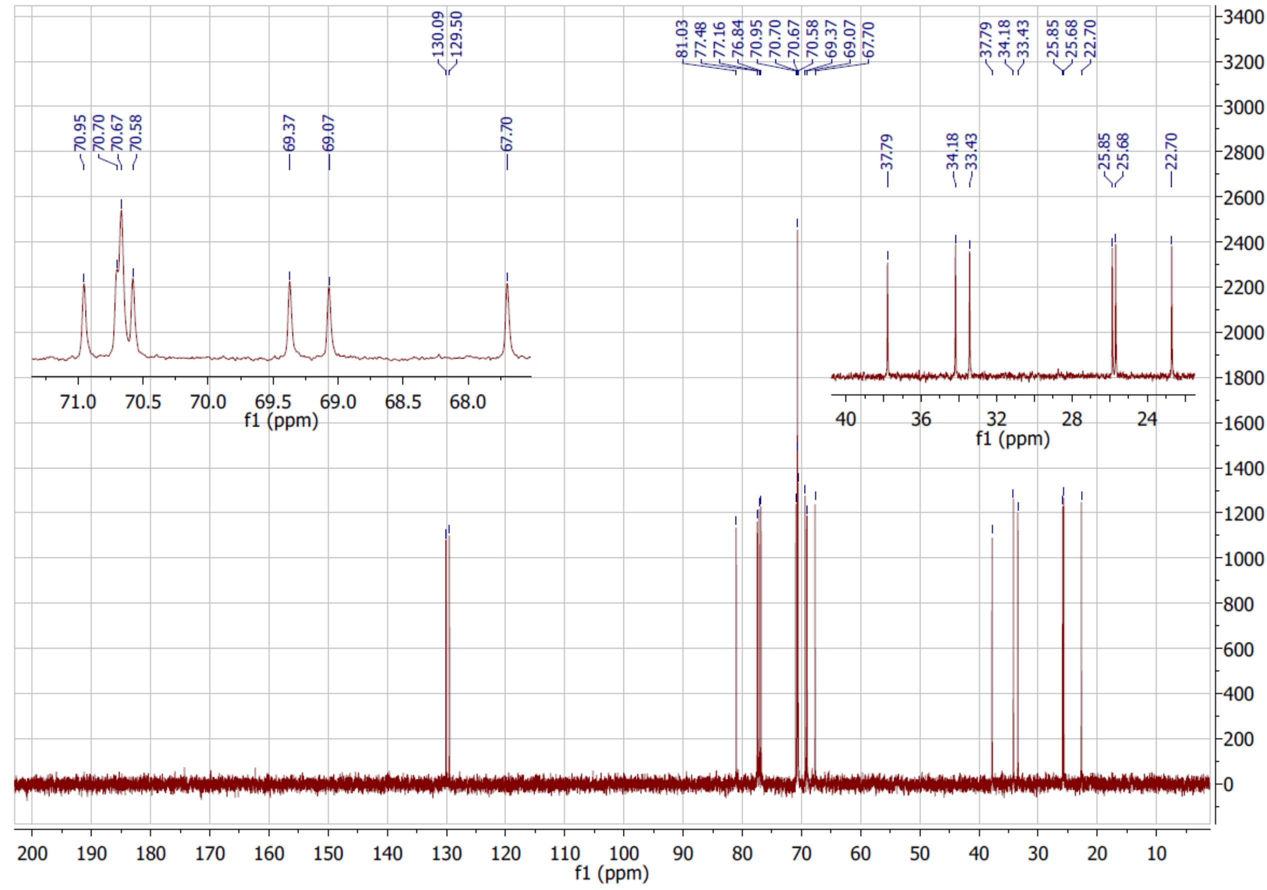

(*Z*)-5-(2-(2-(2-(2-fluoroethoxy)ethoxy)ethoxy)ethoxy)cyclooct-1-ene 9

^{1}H NMR ($CDCl_3$):

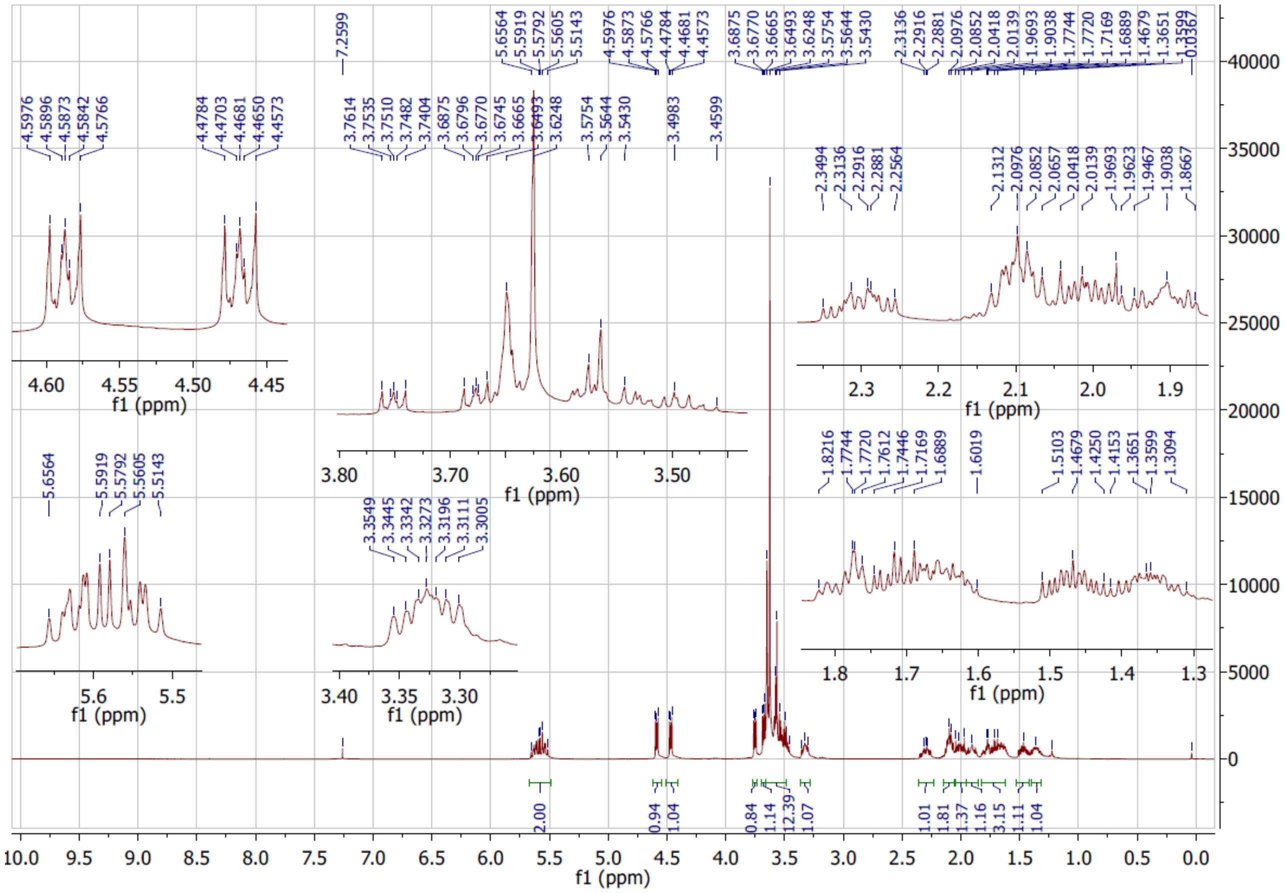

^{13}C NMR ($CDCl_3$):

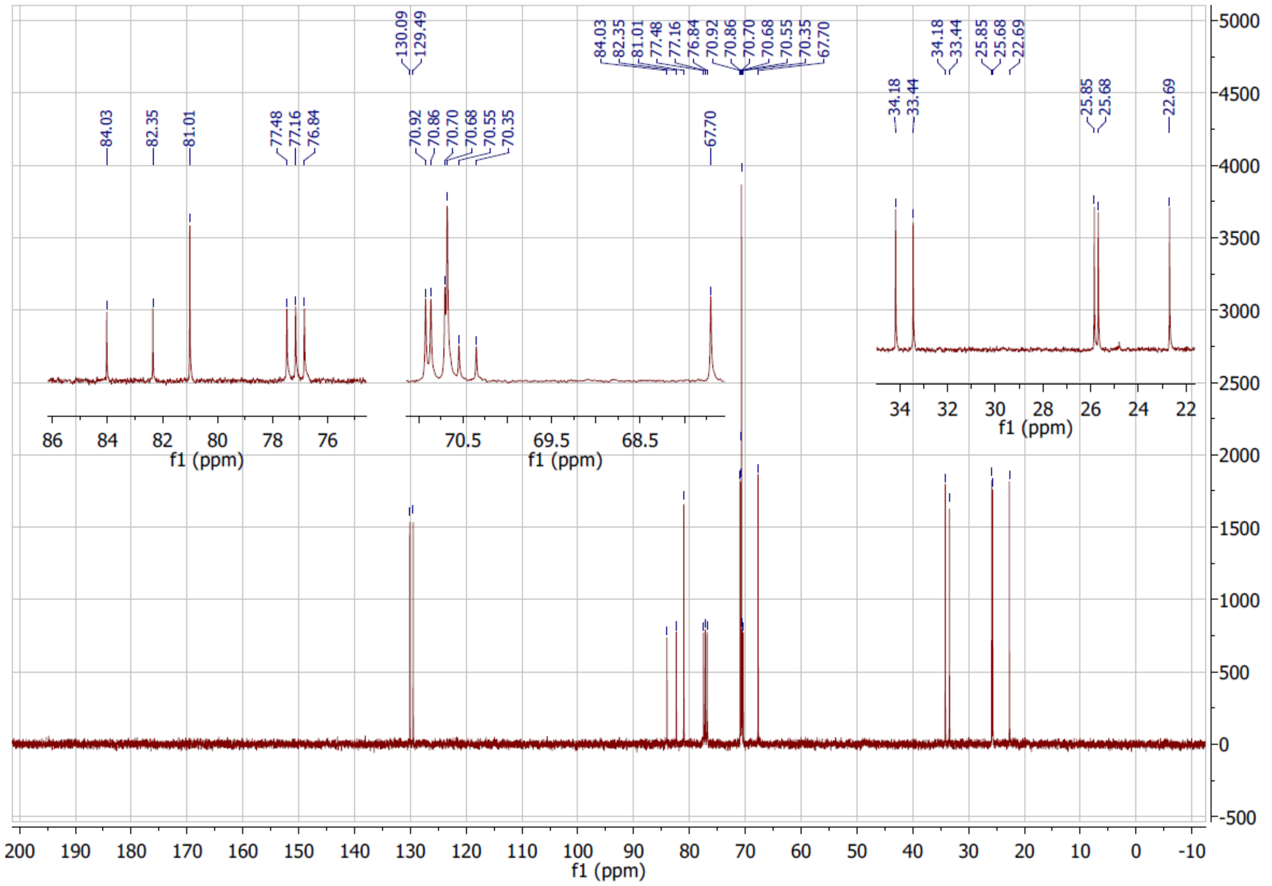

2-(2-(2-(((2*s*,3a*R*,9a*S*,*Z*)-3a,4,5,8,9,9a-hexahydrocycloocta[*d*][1,3]dioxol-2-yl)methoxy)ethoxy)ethoxy)ethyl benzoate 14

^{1}H NMR ($CDCl_3$):

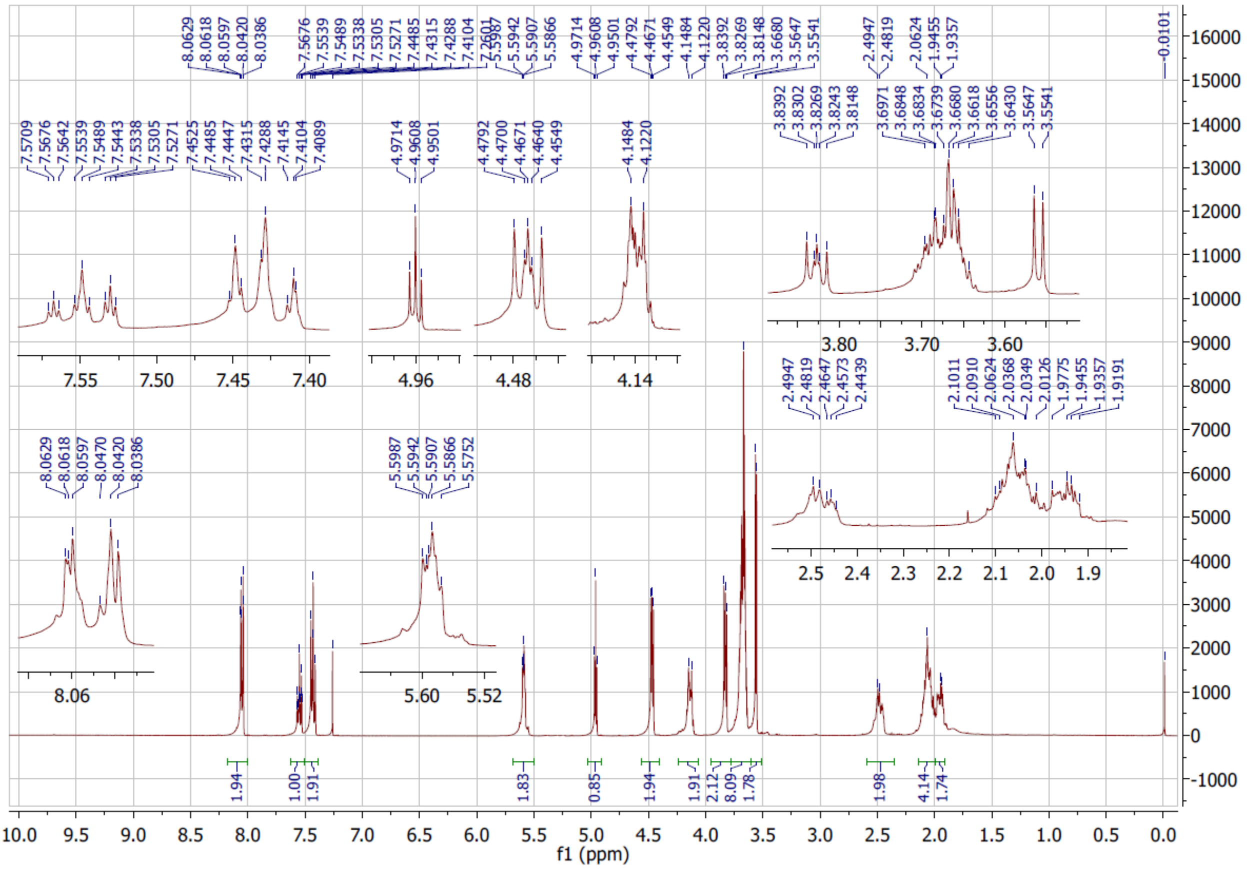

^{13}C NMR ($CDCl_3$):

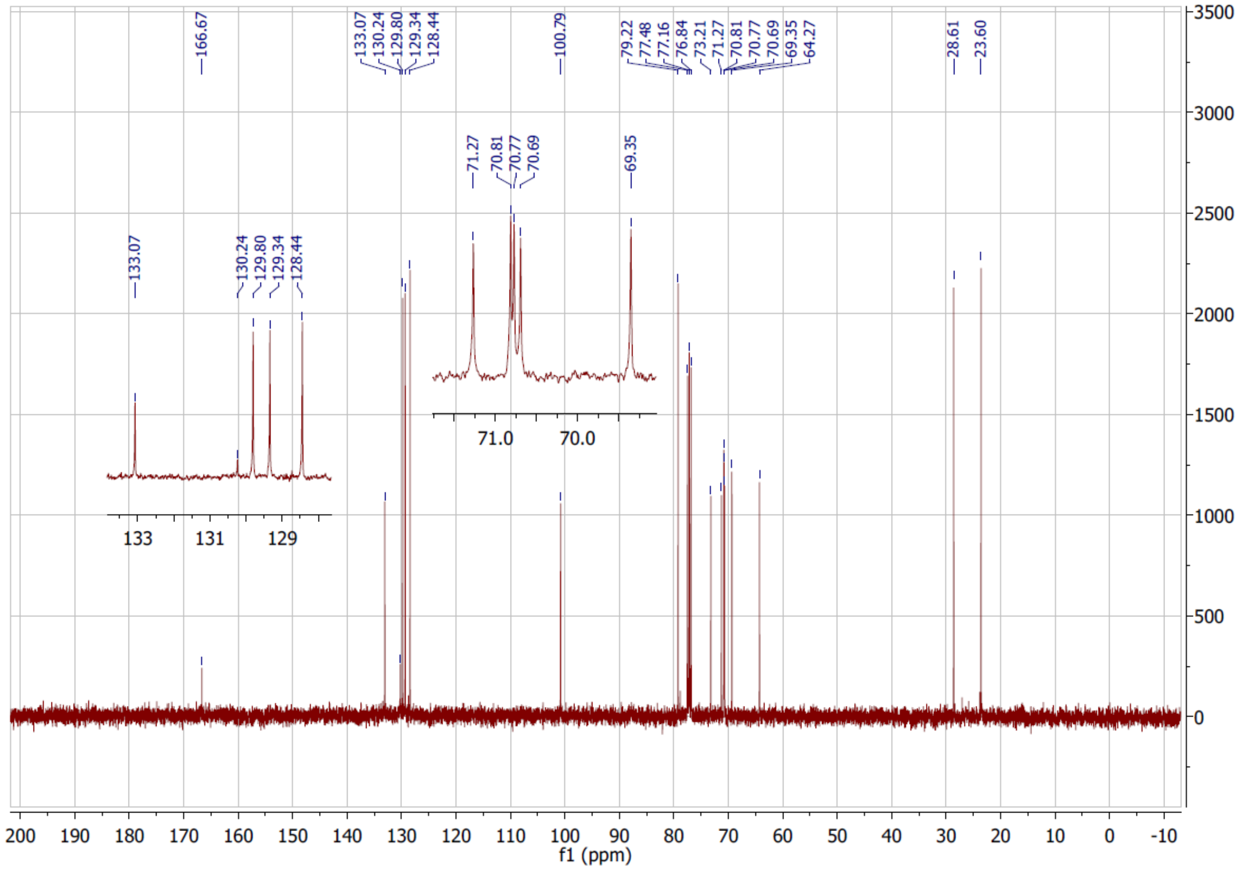

2-(2-(2-((((2*s*,3a*R*,9a*S*,*Z*)-3a,4,5,8,9,9a-hexahydrocycloocta[*d*][1,3]dioxol-2-yl)methoxy)ethoxy)ethoxy)ethanol 15

^{1}H NMR (CDCl$_3$):

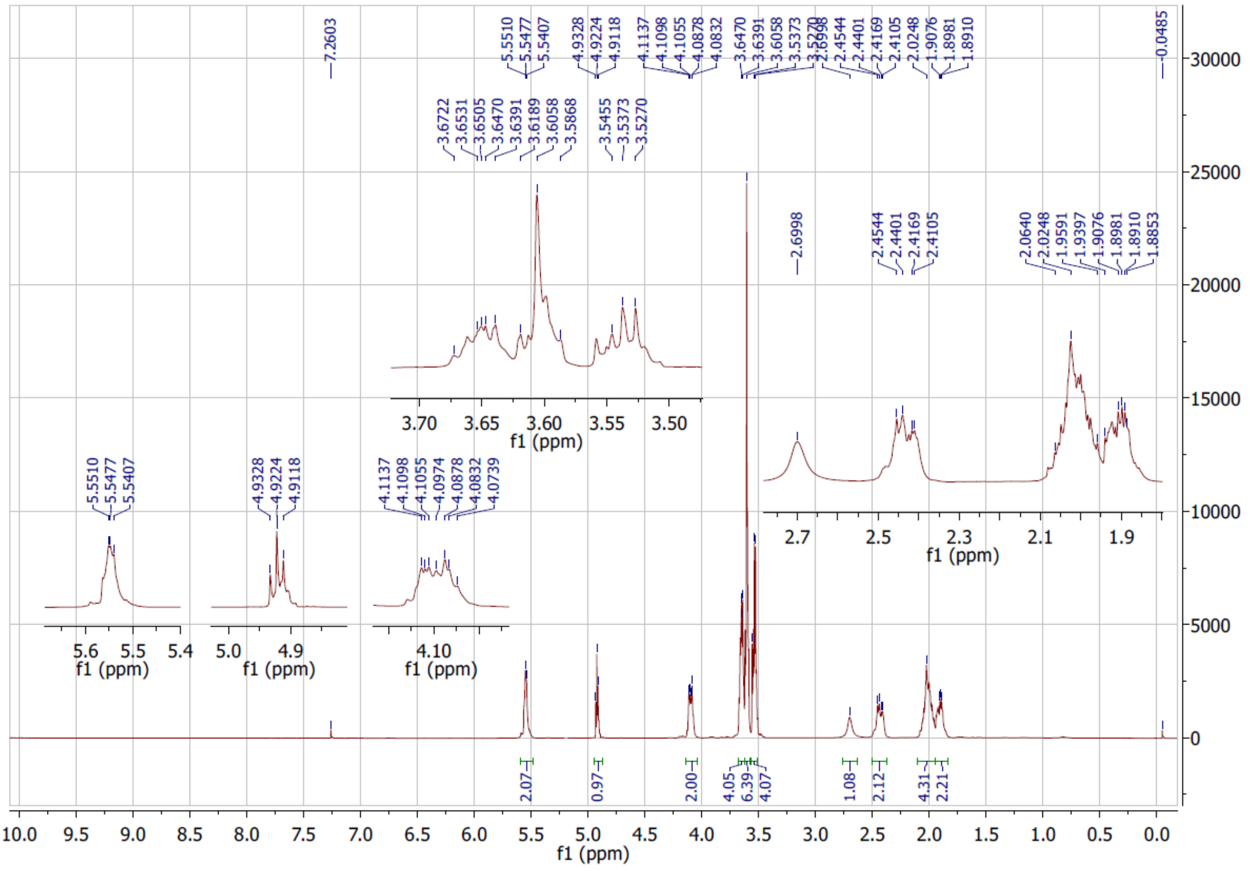

^{13}C NMR (CDCl$_3$):

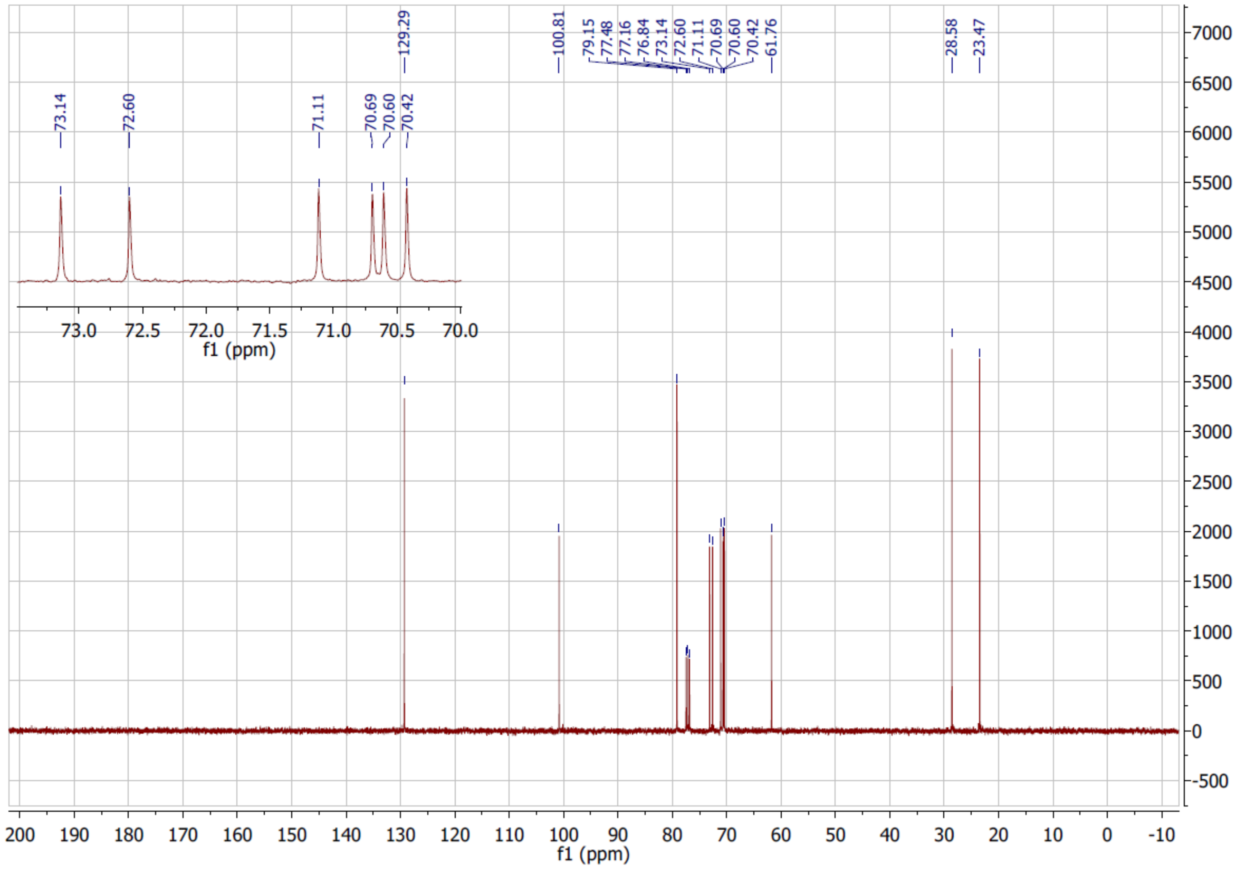

2-(2-(2-(((2*s*,3a*R*,9a*S*,*Z*)-3a,4,5,8,9,9a-hexahydrocycloocta[*d*][1,3]dioxol-2-yl)methoxy)ethoxy)ethoxy)ethyl methanesulfonate 17

^{1}H NMR ($CDCl_3$):

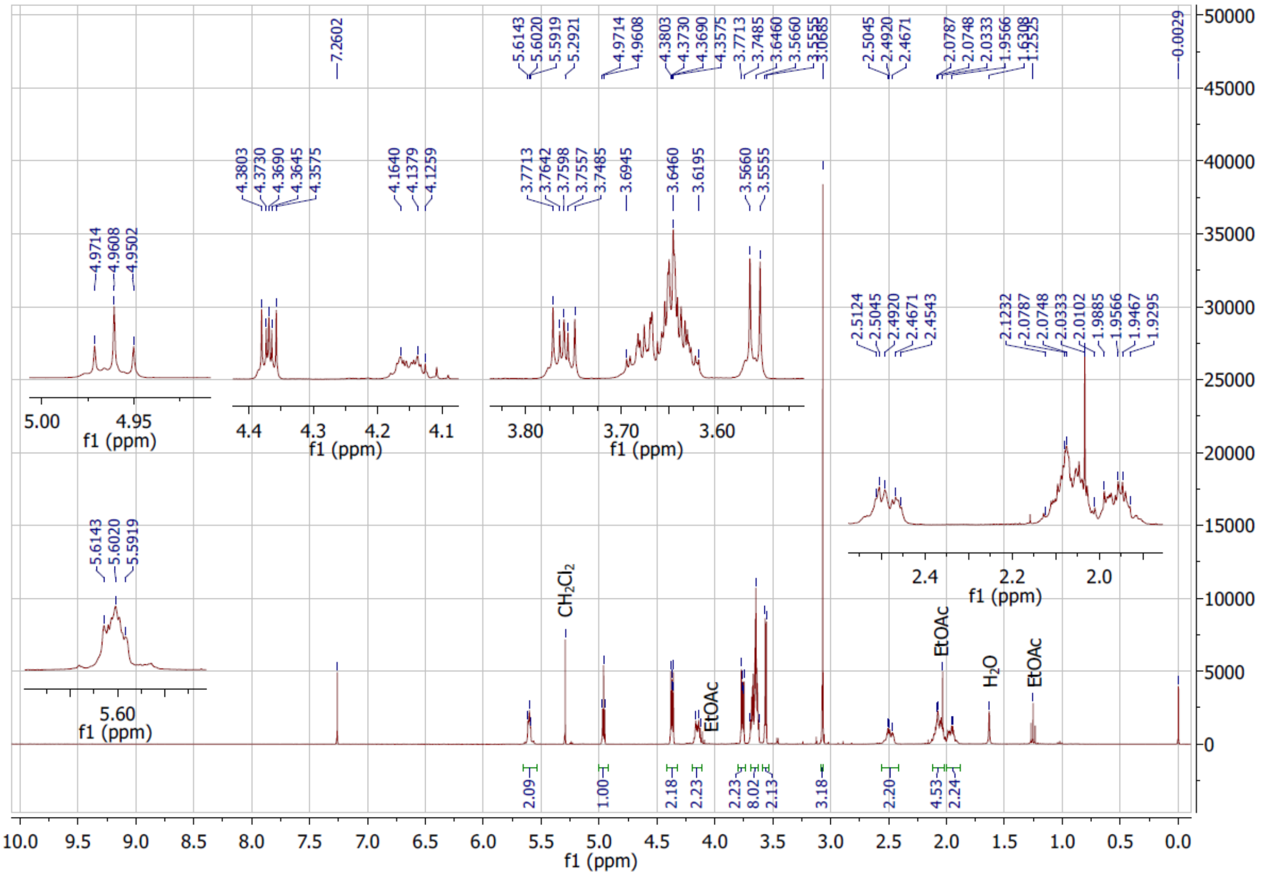

^{13}C NMR ($CDCl_3$):

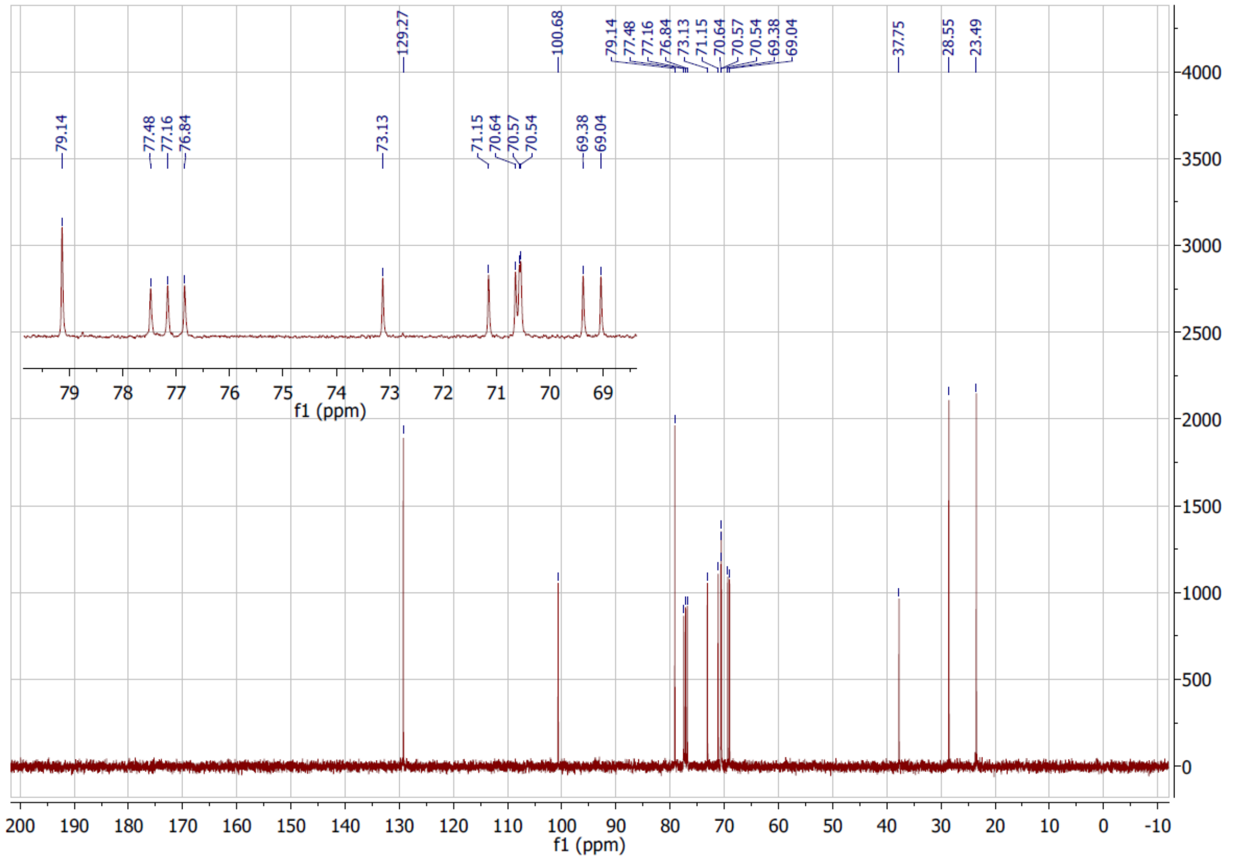

(2*s*,3a*R*,9a*S*,*Z*)-2-((2-(2-(2-fluoroethoxy)ethoxy)ethoxy)methyl)-3a,4,5,8,9,9a-hexahydrocycloocta[*d*][1,3]dioxole 18

^{1}H NMR ($CDCl_3$):

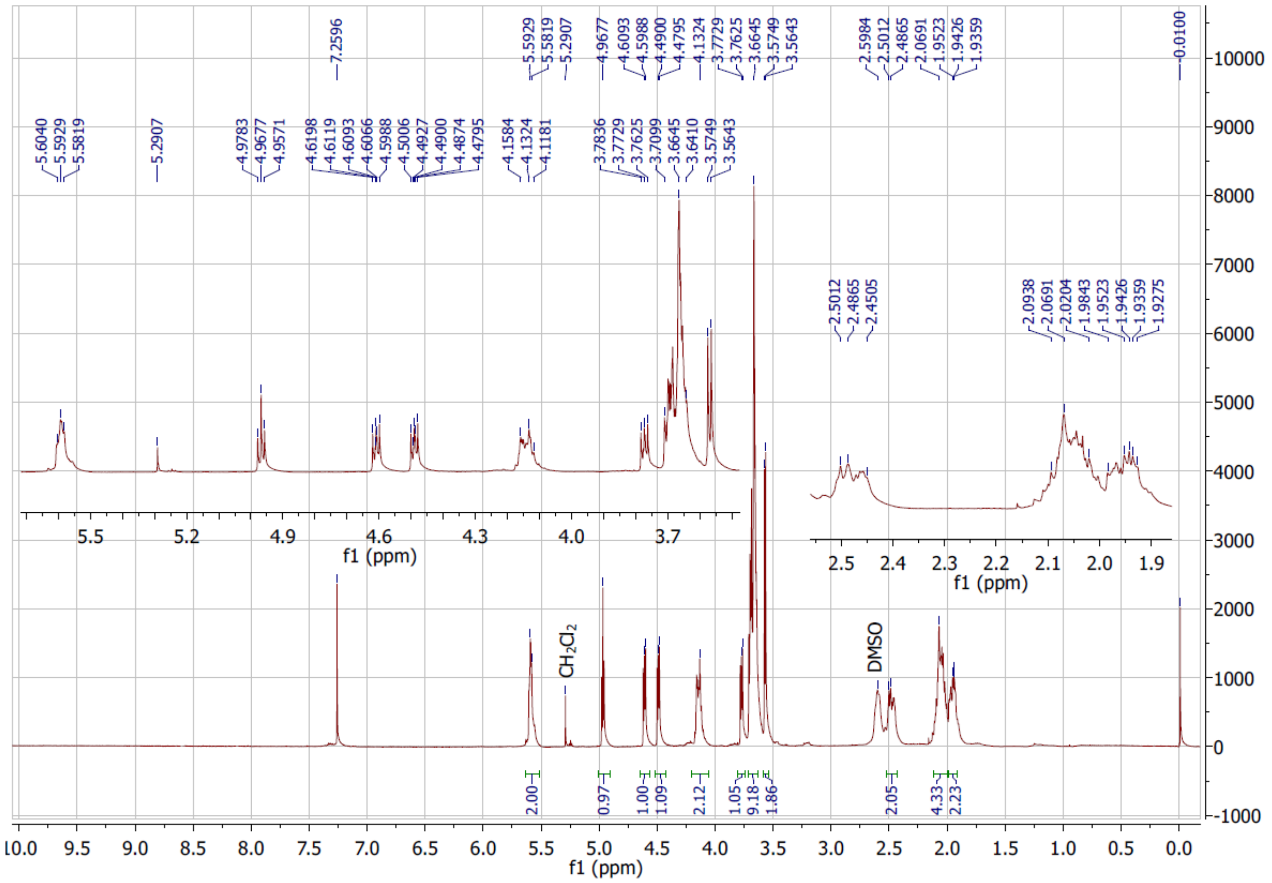

^{13}C NMR ($CDCl_3$):

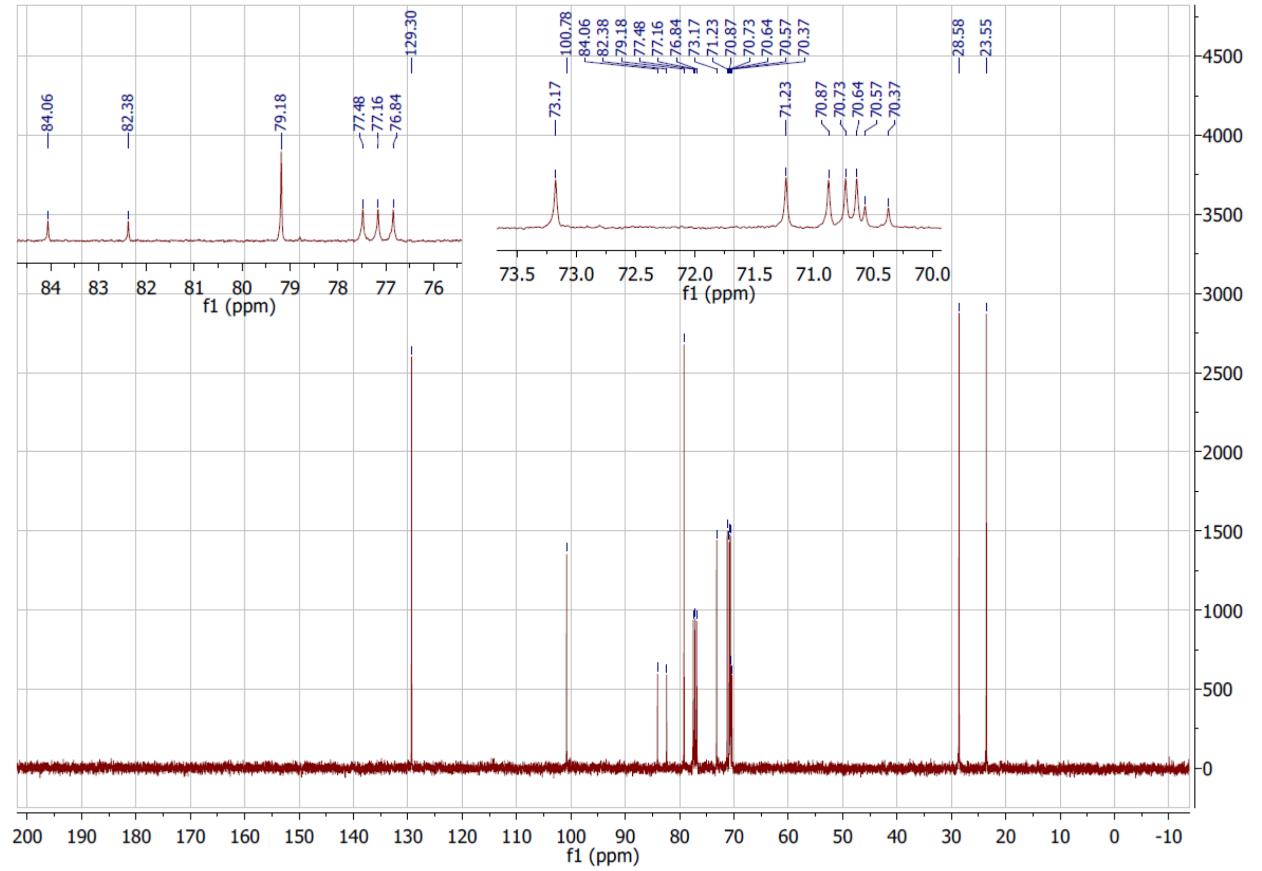

(*S*)-2-(3-((*S*)-1-carboxy-5-(5-oxo-5-((3-(6-(4-(trifluoromethyl)phenyl)-1,2,4,5-tetrazin-3-yl)phenyl)amino)pentanamido)pentyl)ureido)pentanedioic acid 21

^{1}H NMR (CD_3OD):

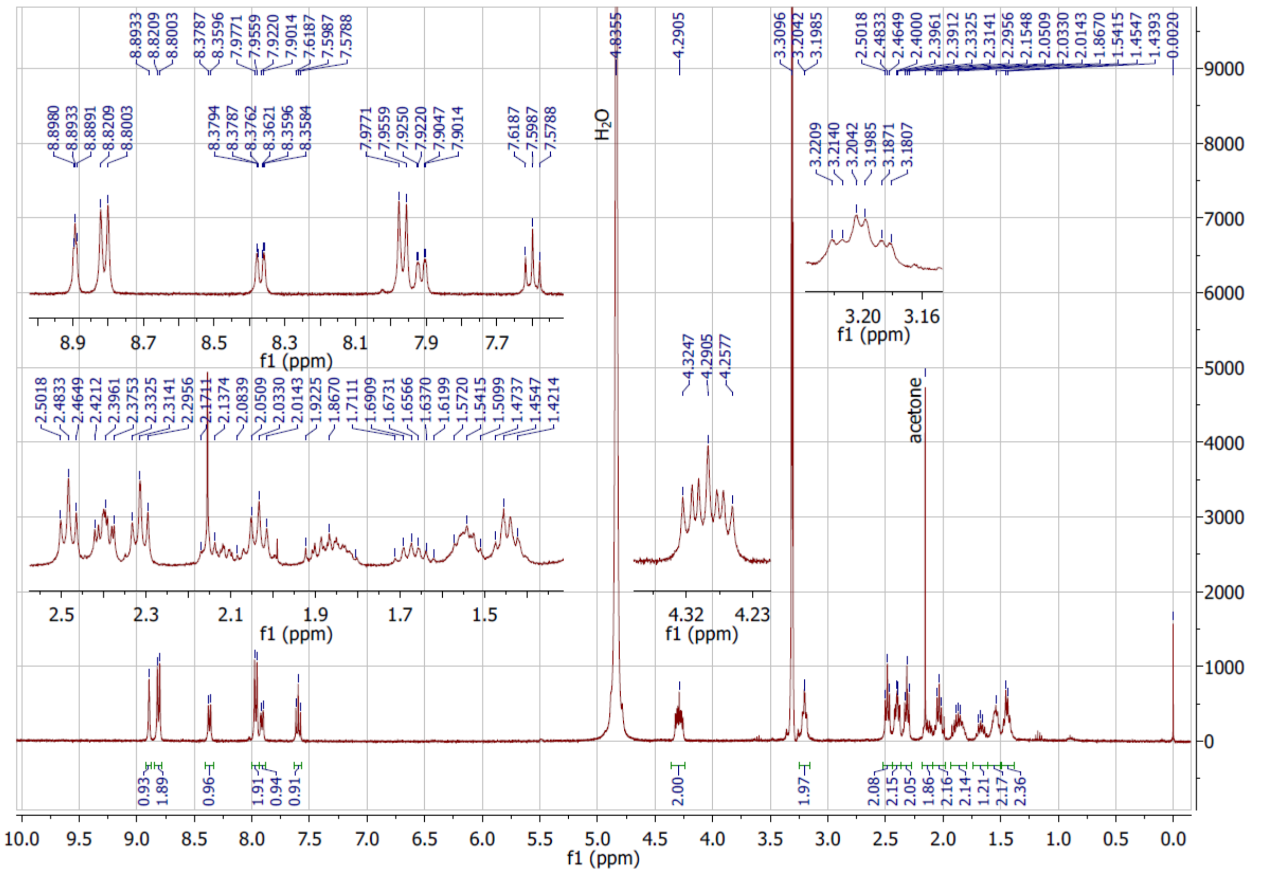

^{13}C NMR (CD_3OD):

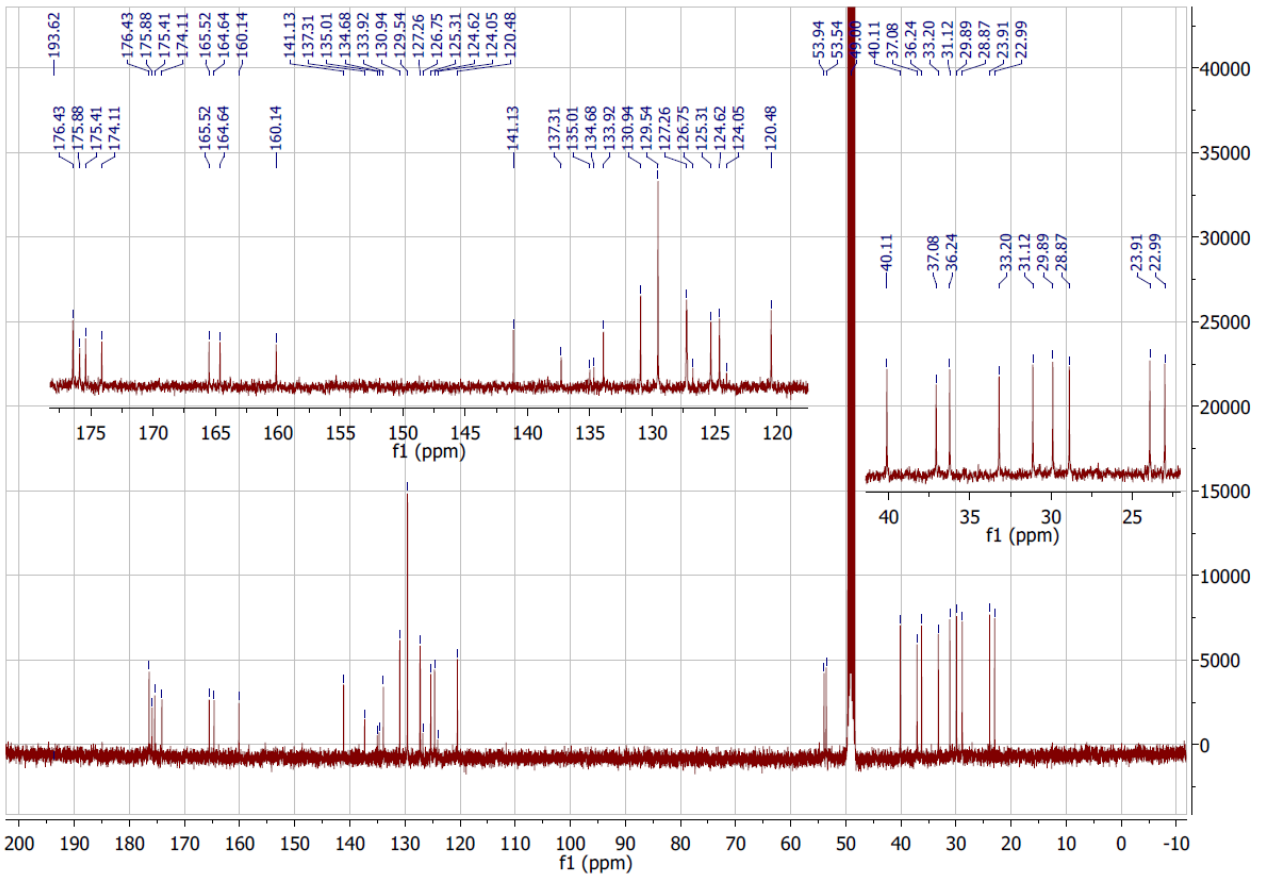

(*E*)-2-(2-(2-(2-(cyclooct-4-en-1-yloxy)ethoxy)ethoxy)ethoxy)ethanol 10a

^{1}H NMR ($CDCl_3$):

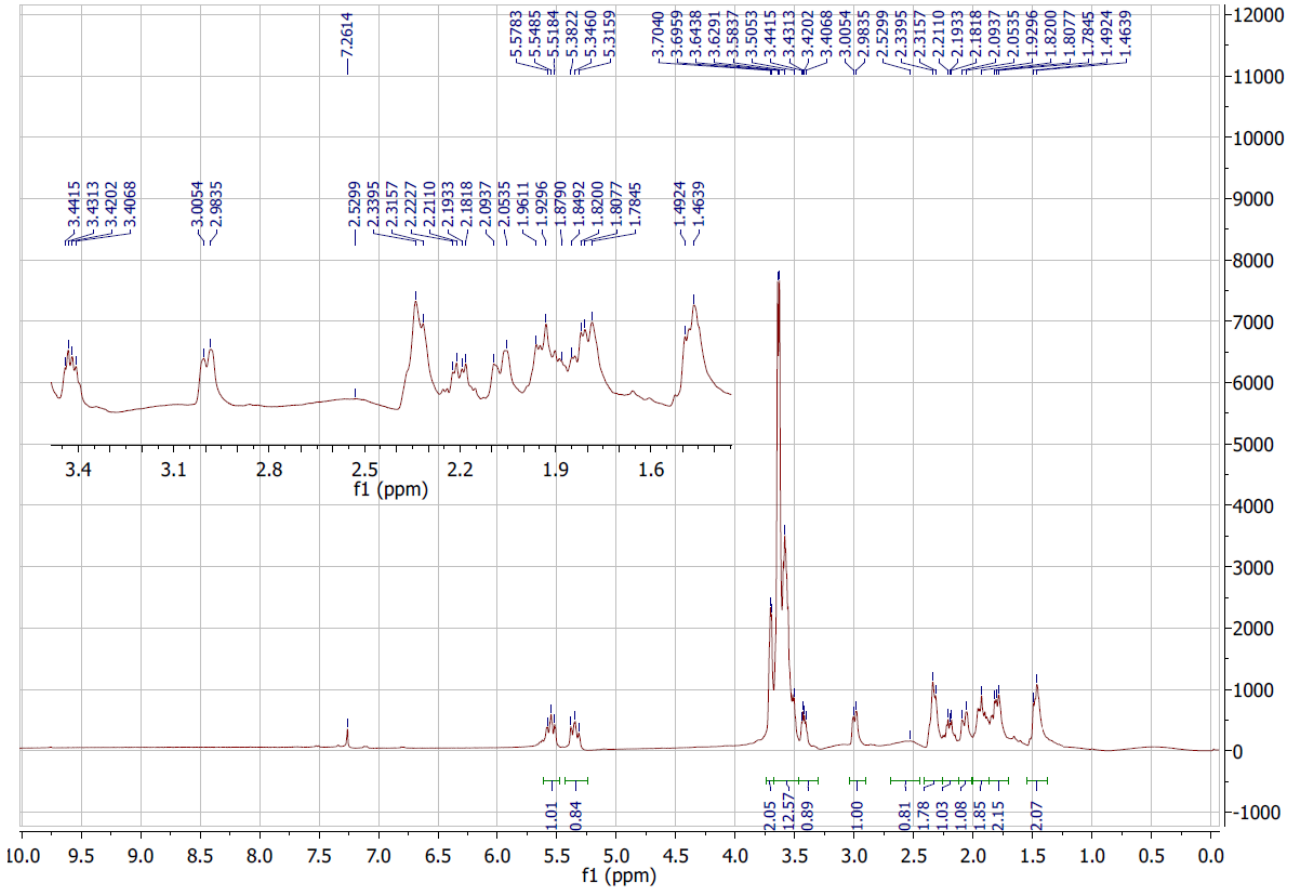

^{13}C NMR ($CDCl_3$):

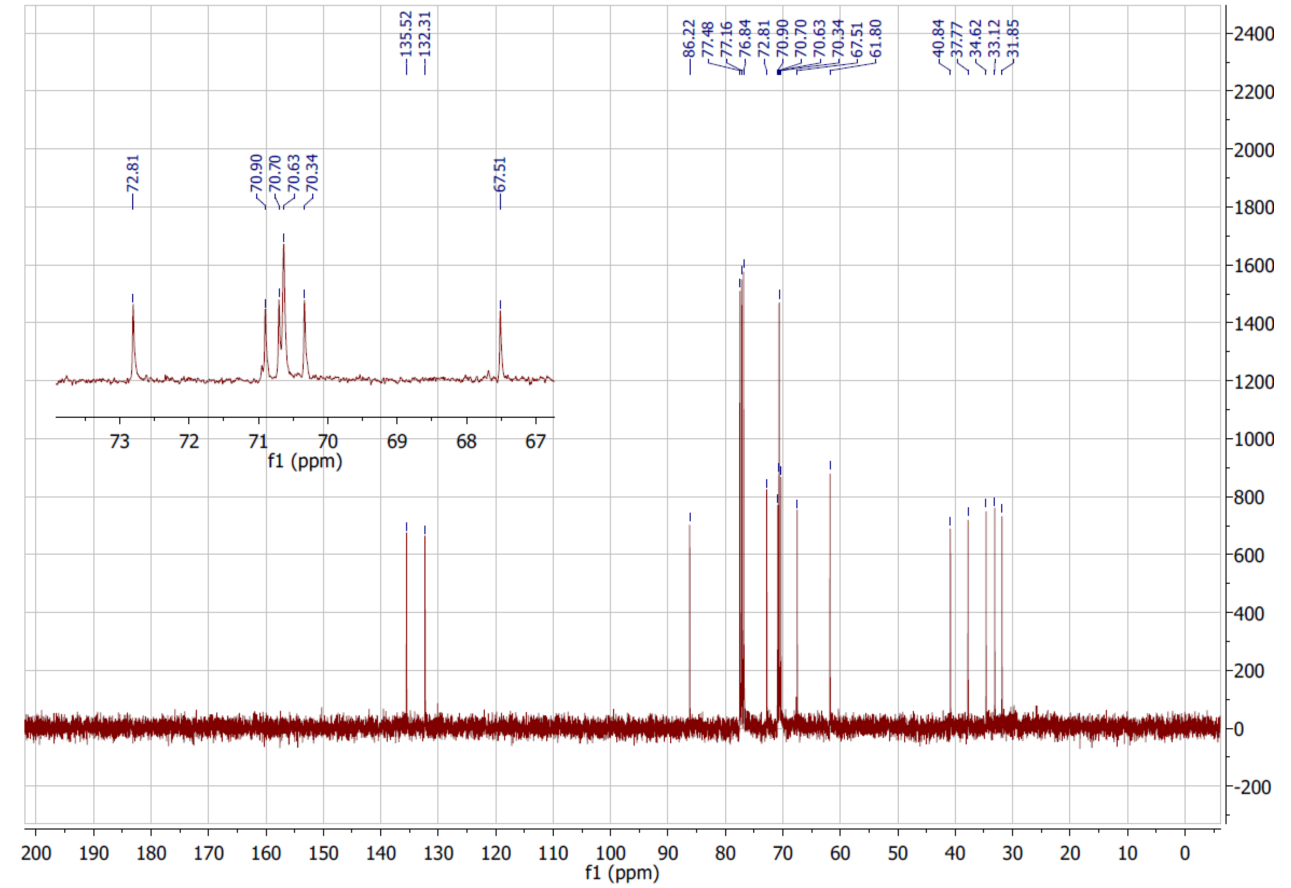

(*E*)-2-(2-(2-(2-(cyclooct-4-en-1-yloxy)ethoxy)ethoxy)ethoxy)ethanol 10b

^{1}H NMR ($CDCl_3$):

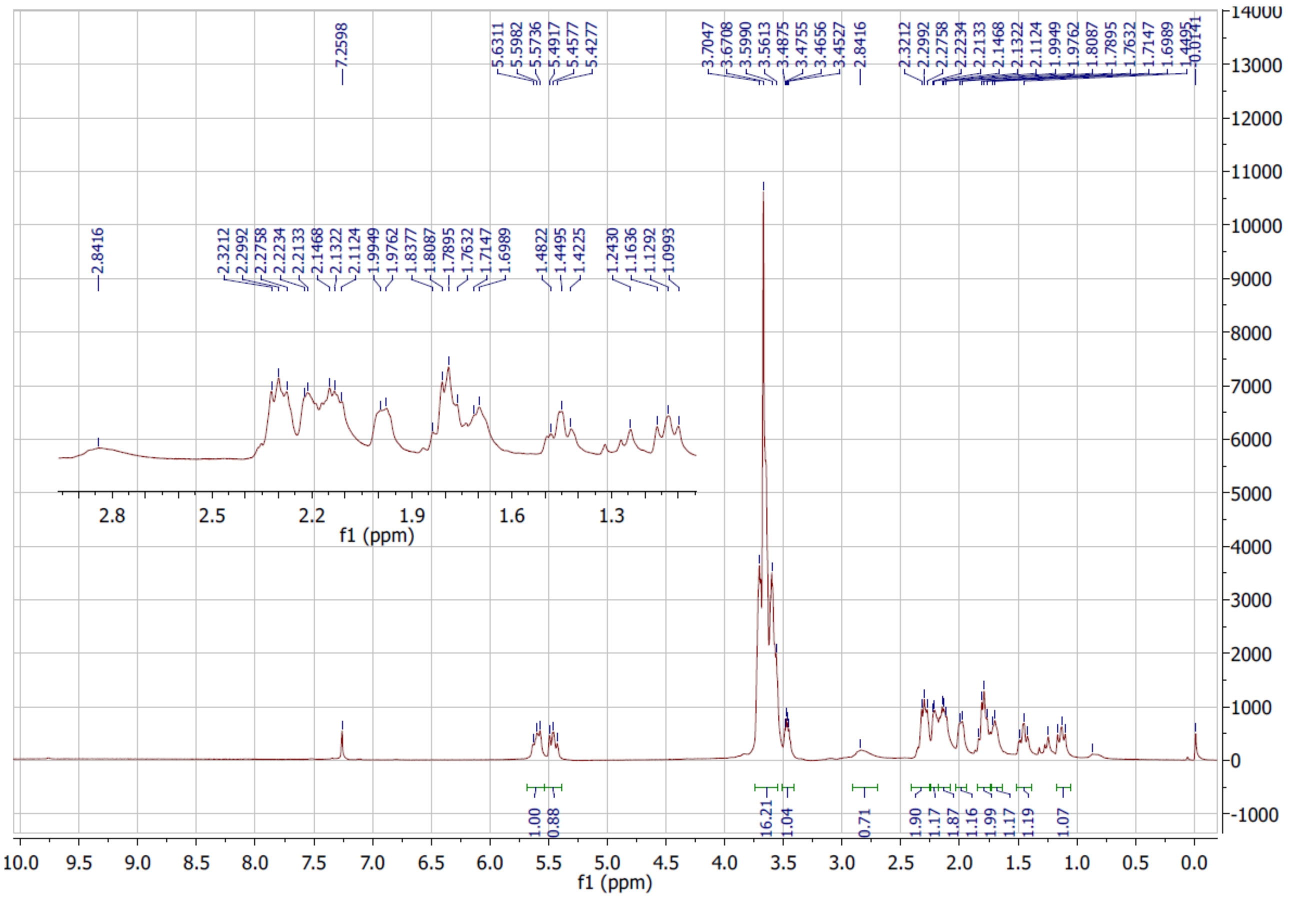

^{13}C NMR ($CDCl_3$):

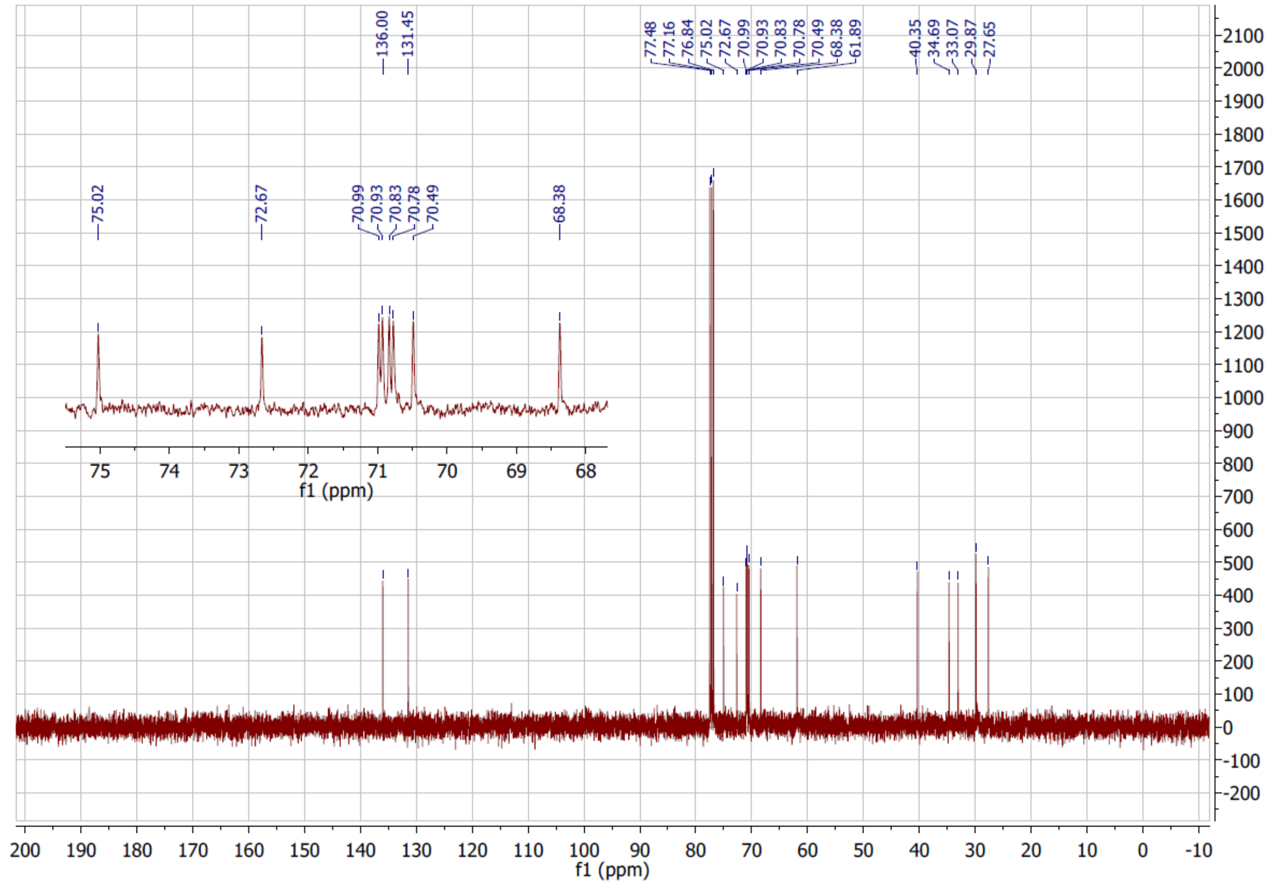

2-(2-(2-(((2*s*,3a*R*,9a*S*,*E*)-3a,4,5,8,9,9a-hexahydrocycloocta[*d*][1,3]dioxol-2-yl)methoxy)ethoxy)ethoxy)ethanol 19

^{1}H NMR (CDCl$_3$):

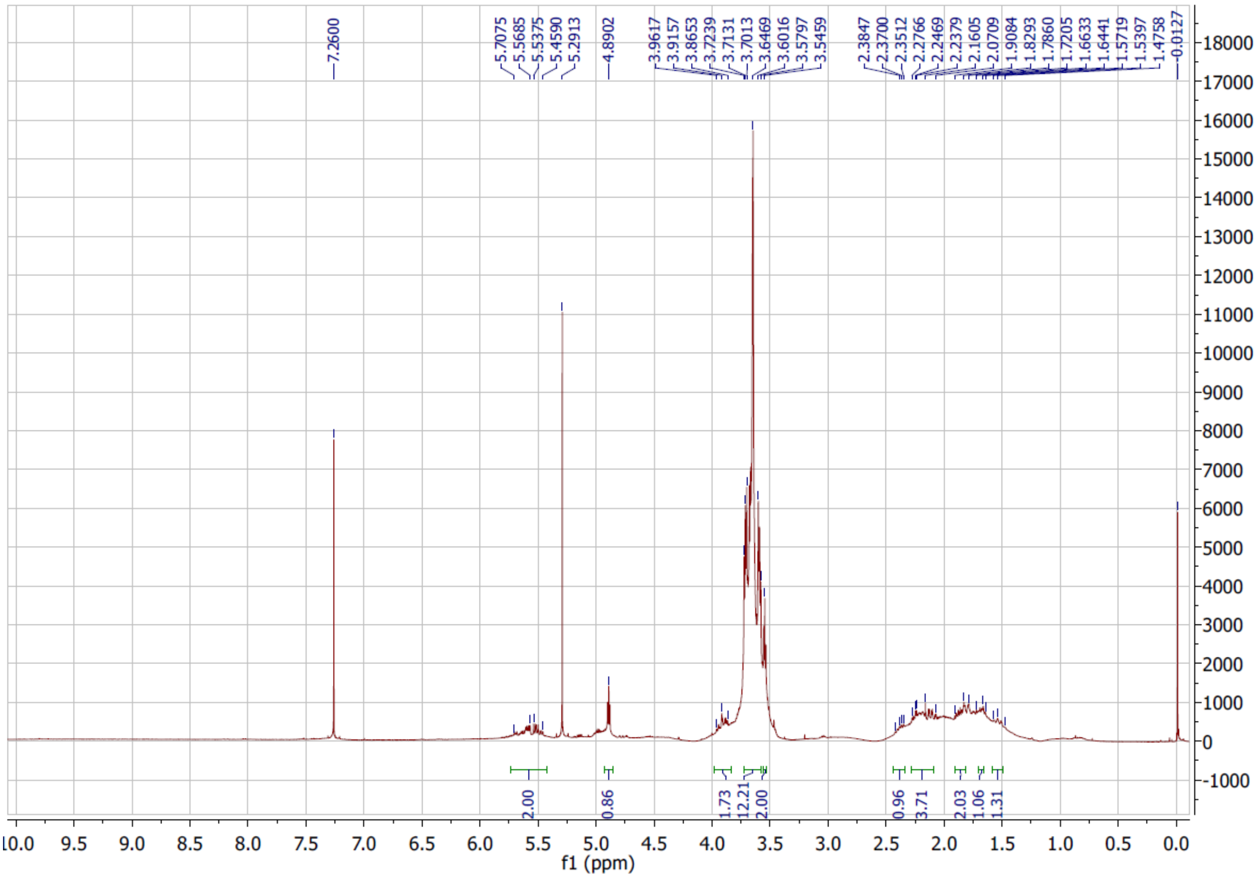

^{13}C NMR (CDCl$_3$):

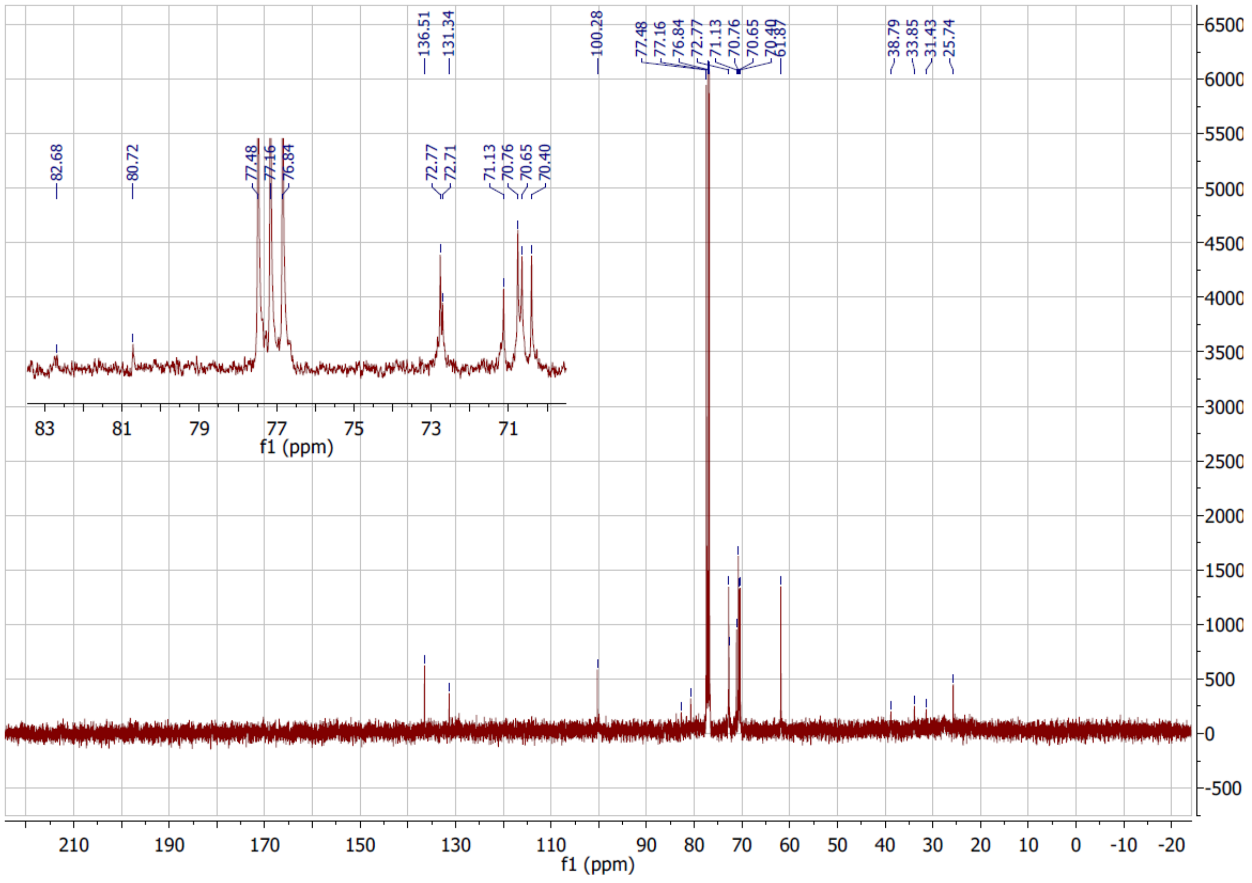

(*E*)-5-(2-(2-(2-(2-fluoroethoxy)ethoxy)ethoxy)ethoxy)cyclooct-1-ene 2a

^{1}H NMR ($CDCl_3$):

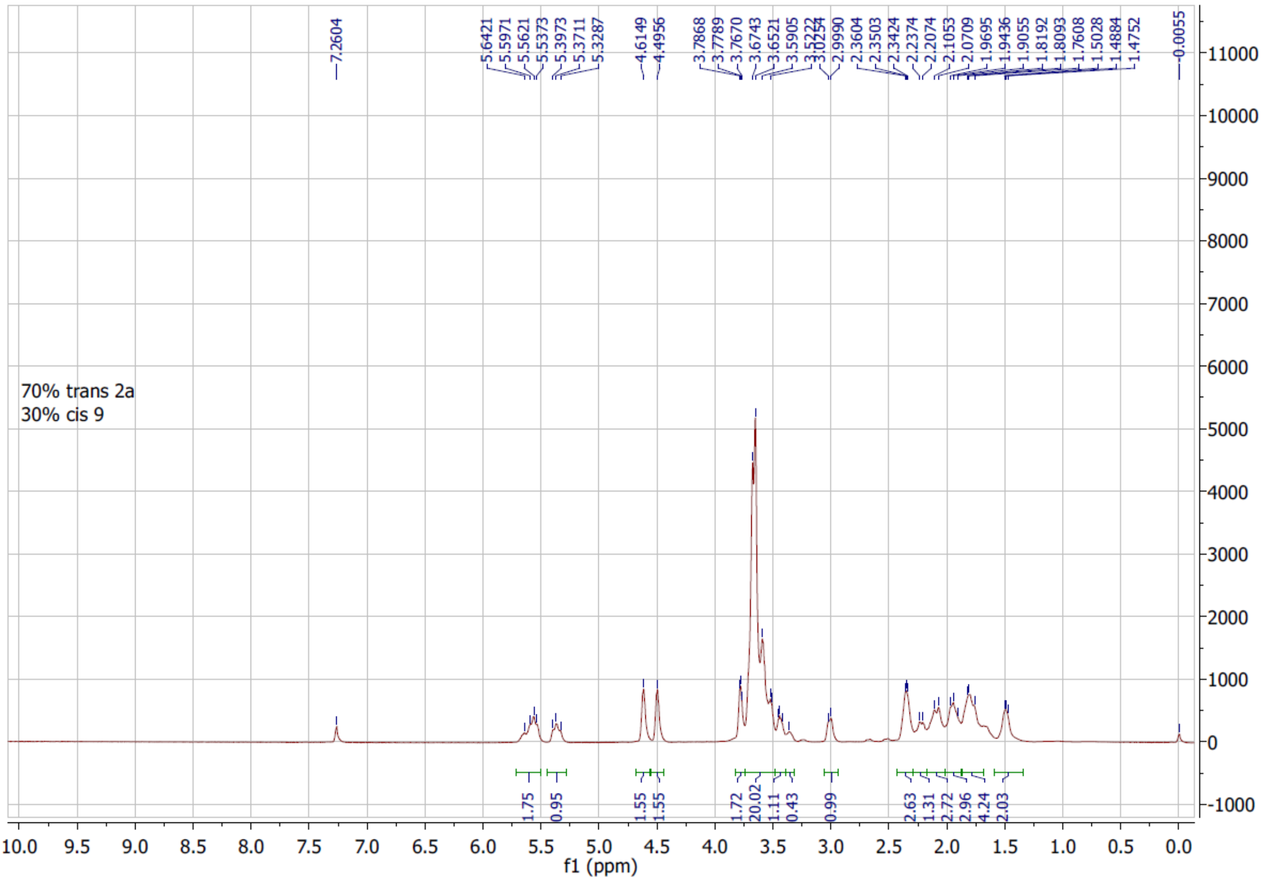

^{13}C NMR ($CDCl_3$):

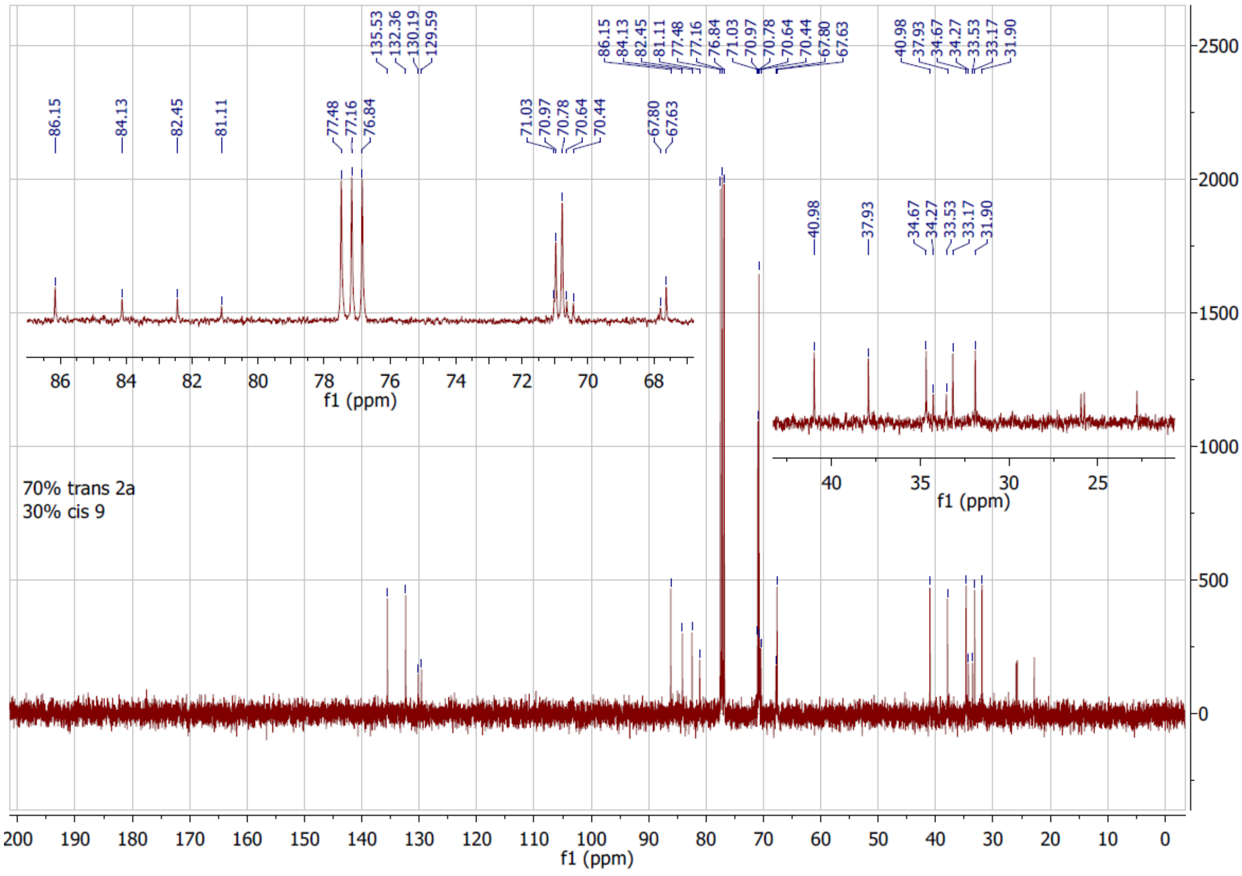

(*E*)-5-(2-(2-(2-(2-fluoroethoxy)ethoxy)ethoxy)ethoxy)cyclooct-1-ene 2b

^{1}H NMR ($CDCl_3$):

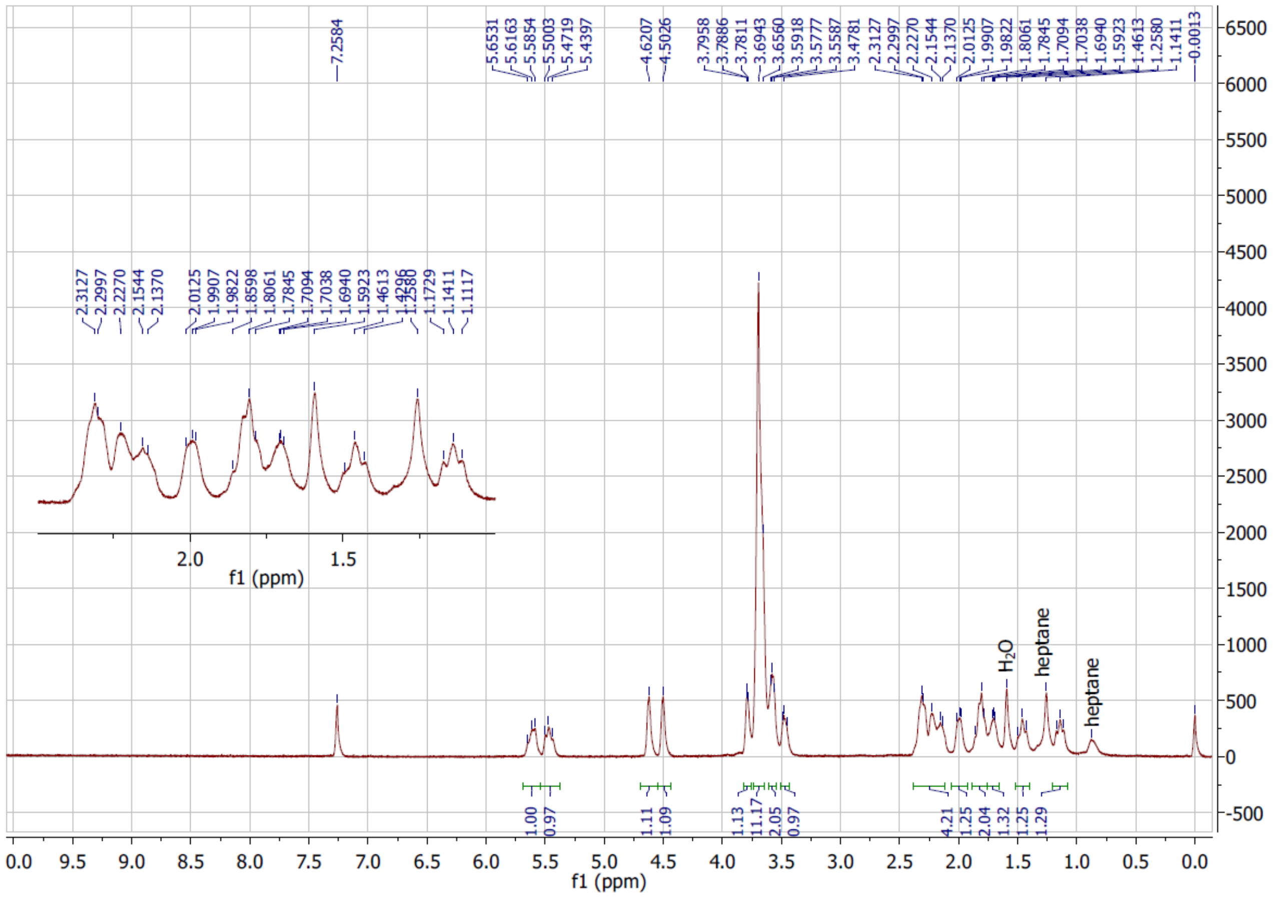

^{13}C NMR ($CDCl_3$):

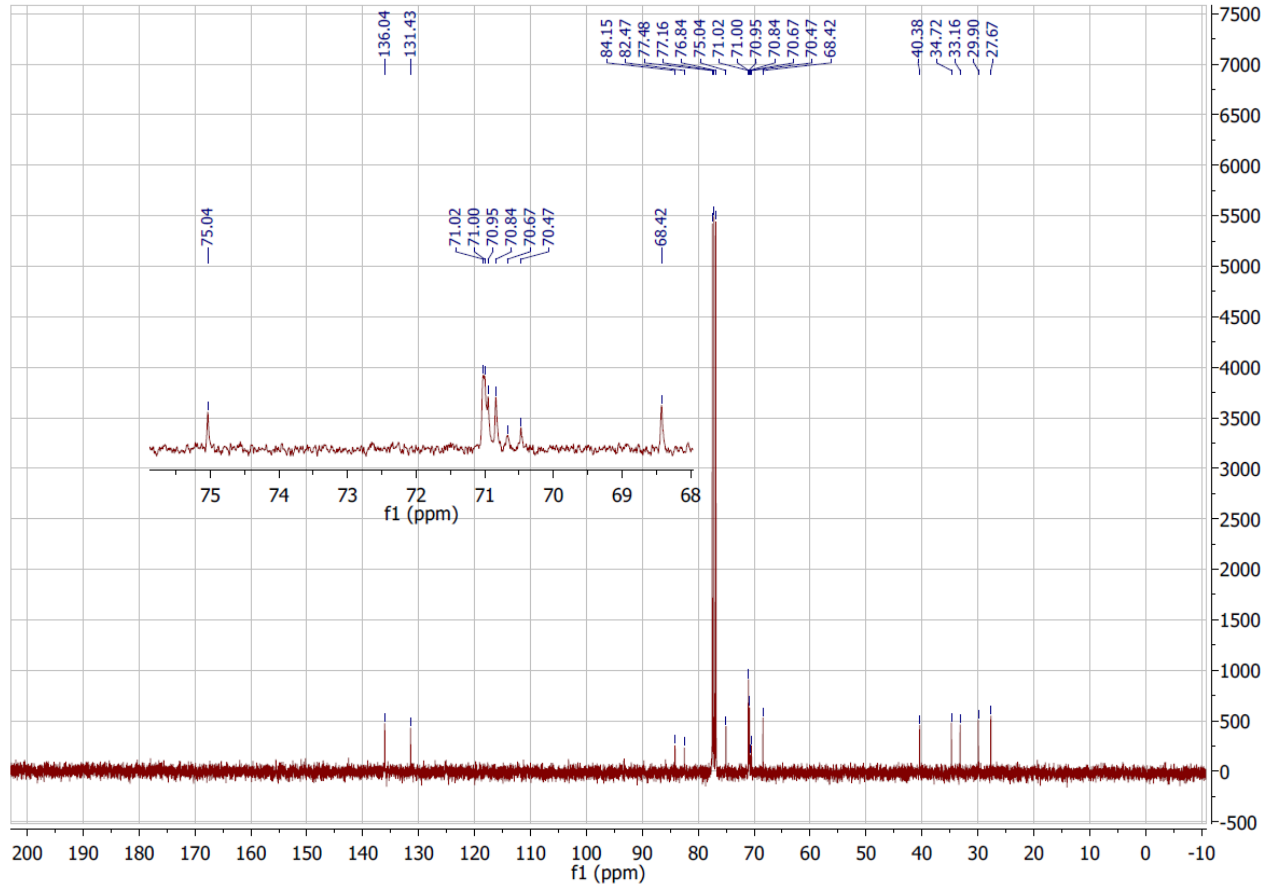

(2*s*,3a*R*,9a*S*,*E*)-2-((2-(2-(2-fluoroethoxy)ethoxy)ethoxy)methyl)-3a,4,5,8,9,9a-hexahydrocycloocta[*d*][1,3]dioxole 3

^{1}H NMR ($CDCl_3$):

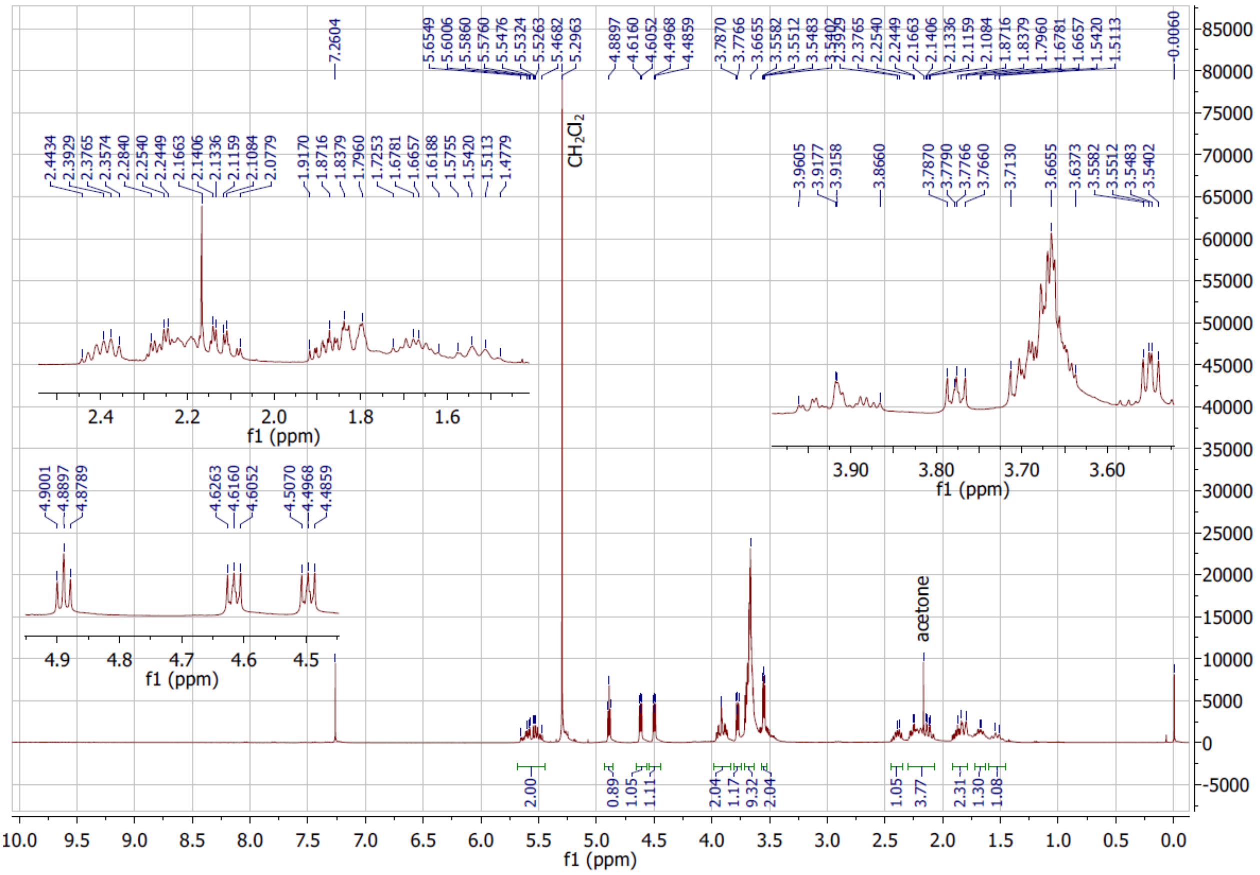

^{13}C NMR ($CDCl_3$):

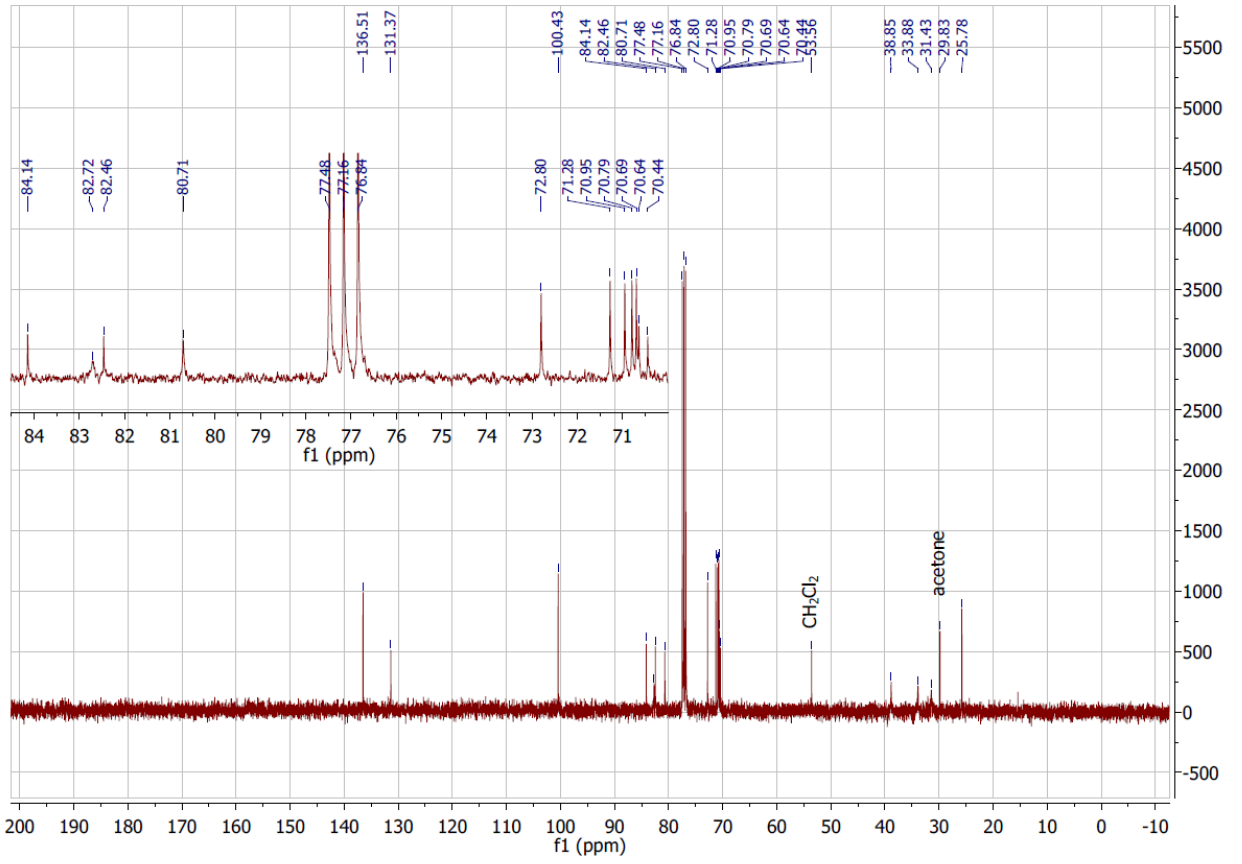

2-(2-(2-(((2*s*,3a*R*,9a*S*,*E*)-3a,4,5,8,9,9a-hexahydrocycloocta[*d*][1,3]dioxol-2-yl)methoxy)ethoxy)ethoxy)ethyl methanesulfonate 20

^{1}H NMR ($CDCl_3$):

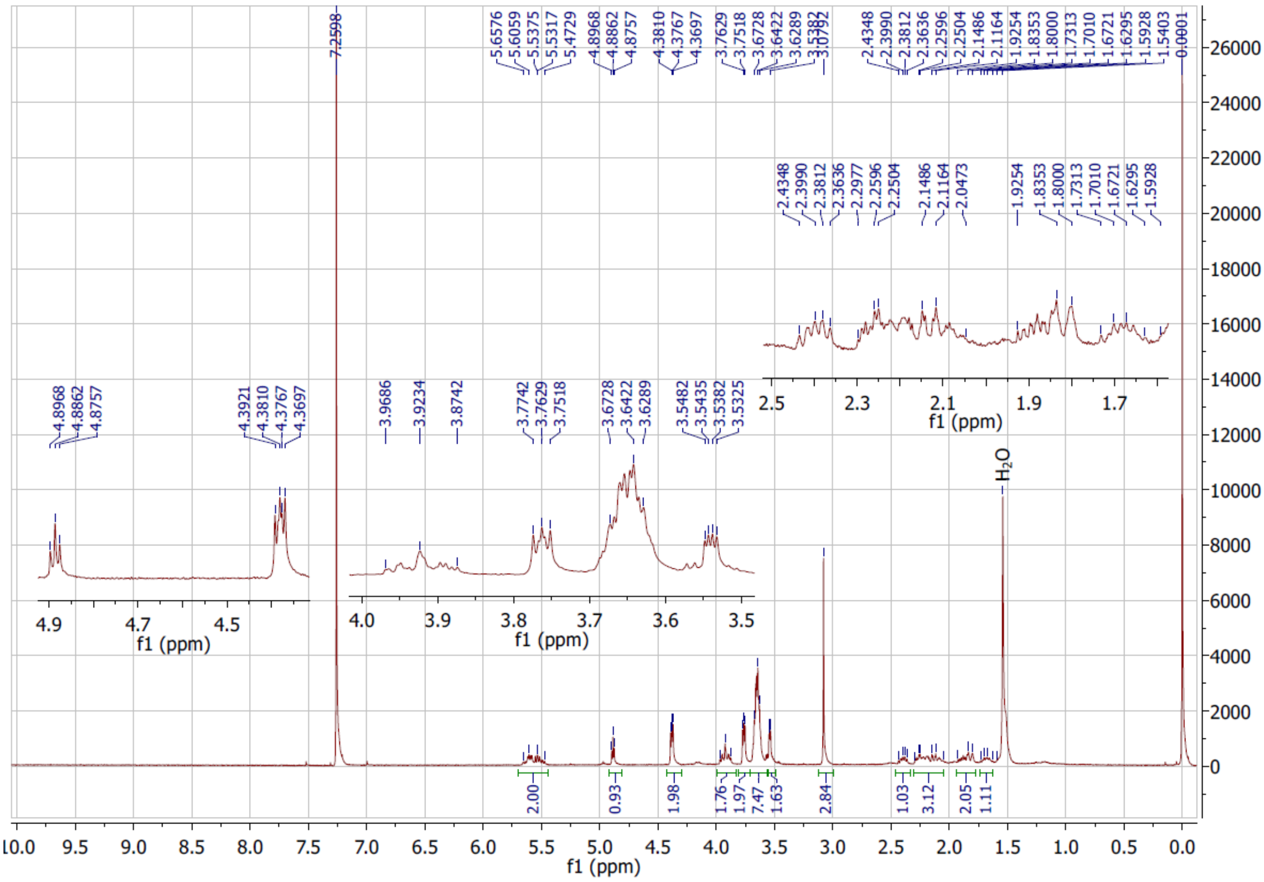

^{13}C NMR ($CDCl_3$):

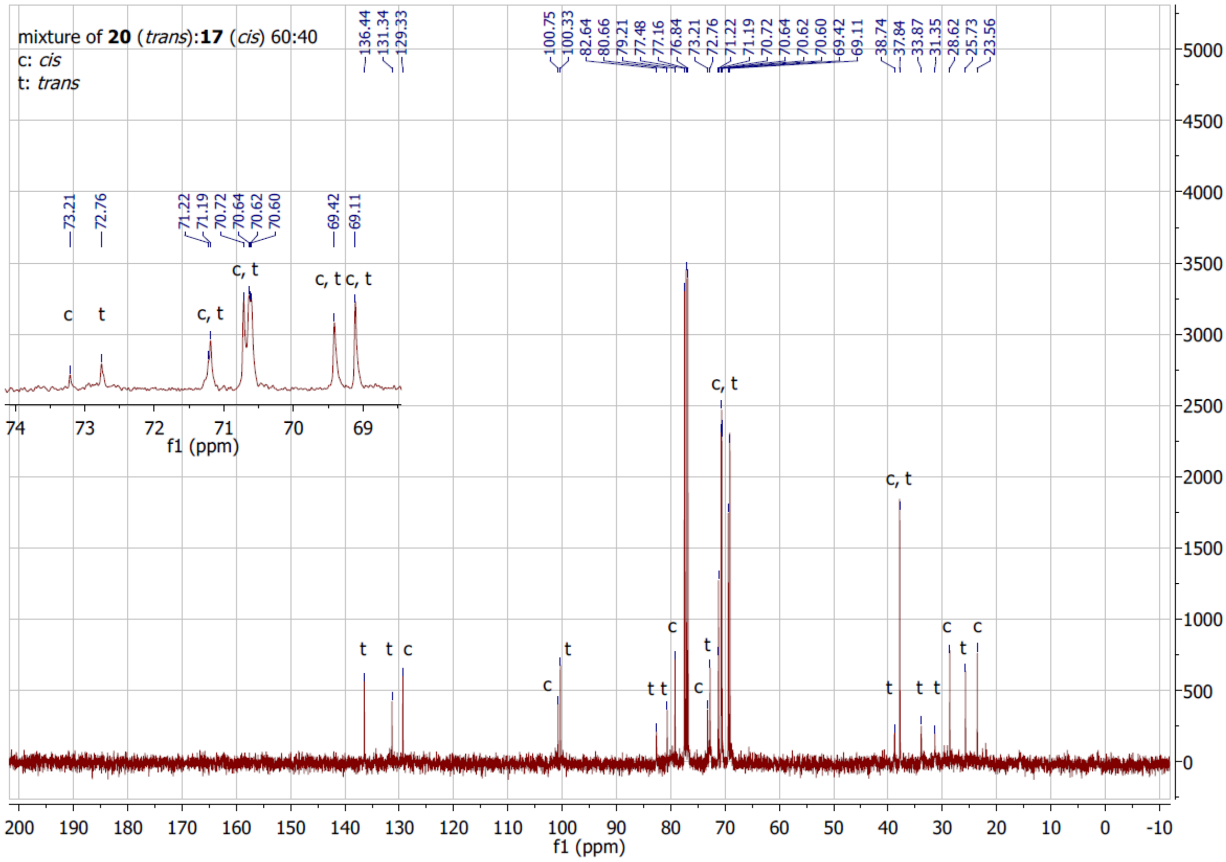

12. References

1 B. S. B. Gxoyiya, P. T. Kaye and C. Kenyon, *Synthetic Communications* 2010, **40**, 2578.

2 V. Bhushan, R. Rathore and S. Chandrasekaran, *Synthesis Communications* 1984, 431.

3 A. Darko, S. Wallace, O. Dmitrenko, M. M. Machovinac, R. A. Mehl, J. W. Chin and J. M. Fox, *Chem. Sci.* 2014, **5**, 3770.

4 R. Selvaraj, B. Giglio, S. Liu, H. Wang, M. Wang, H. Yuan, S. R. Chintala, L-P Yap, P. S. Conti, J. M. Fox and Z. Li, *Bioconjugate Chem.* 2015, **26**, 435.

5 K. P. Maresca, S. M. Hillier, F. J. Femia, D. Keith, C. Barone, J. L. Joyal, C. N. Zimmerman, A. P. Kozikowski, J. A. Barrett, W. C. Eckelman and J. W. Babich, *J. Med. Chem.* 2009, **52**, 347.

6 M. Royzen, G. P. A. Yap and J. M. Fox, *J. Am. Chem. Soc.* 2008, **130**, 3760.
